# Eosinophil Extracellular Traps in Health and Disease: From Host Defense to Chronic Inflammation

**DOI:** 10.3390/biomedicines14081740

**Published:** 2026-08-01

**Authors:** Bojan Stojanovic, Bojana Djokic, Bojana S. Stojanovic, Milica Dimitrijevic Stojanovic, Jovan Jovanovic, Aleksandar Matic

**Affiliations:** 1 Department of Surgery, Faculty of Medical Sciences, University of Kragujevac, 34000 Kragujevac, Serbia; bojan.stojanovic01@gmail.com (B.S.);; 2 Center for Molecular Medicine and Stem Cell Research, Faculty of Medical Sciences, University of Kragujevac, 34000 Kragujevac, Serbia; 3 Department of Internal Medicine, Clienia Littenheid AG, 9573 Littenheid, Switzerland; 4 Department of Pathophysiology, Faculty of Medical Sciences, University of Kragujevac, 34000 Kragujevac, Serbia; 5 Department of Pathology, Faculty of Medical Sciences, University of Kragujevac, 34000 Kragujevac, Serbia; 6 Department of Biomedical Sciences, State University of Novi Pazar, 36300 Novi Pazar, Serbia; 7 Clinic of Cardiology, University Clinical Centre Kragujevac, 34000 Kragujevac, Serbia

**Keywords:** eosinophils, eosinophil extracellular traps, EETosis, cytolysis, mitochondrial DNA, granule proteins, type 2 inflammation, host defense, tissue injury, cancer

## Abstract

Eosinophils are tissue-adapted granulocytes with important roles in host defense, immune regulation, barrier responses, and inflammatory disease. Beyond classical degranulation, activated eosinophils can release eosinophil extracellular traps (EETs), extracellular DNA and protein structures that arise through cytolytic nuclear EETosis or rapid mitochondrial DNA (mtDNA) release. These traps may contribute to host protection by immobilizing pathogens and concentrating eosinophil granule proteins at sites of mucosal or tissue inflammation. However, when EET formation is excessive, persistent, or insufficiently cleared, the same structures may promote epithelial injury, mucus viscosity, thromboinflammation, tissue remodeling, fibrosis, and chronic eosinophilic inflammation. This review summarizes the cellular mechanisms, structural organization, inducing stimuli, and disease relevance of EETs. Particular attention is given to infectious settings, eosinophilic airway and middle-ear disease, systemic vasculitic and hypereosinophilic disorders, inflammatory skin and ocular diseases, eosinophilic gastrointestinal disease, and cancer. Current evidence indicates that EETs should be interpreted as context-dependent eosinophil effector structures with dual biological potential. Although their mechanistic relevance is increasingly recognized, further histological, functional, and clinical validation is needed before EETs can be established as reliable biomarkers or therapeutic targets.

## 1. Introduction

Eosinophils have traditionally been associated with type 2 immunity, allergic inflammation, and host defense against extracellular parasites, but it is now clear that their biological role is broader and strongly shaped by the tissue environment. As tissue-active granulocytes, eosinophils can contribute to immune regulation, barrier defense, repair, and resolution of inflammation, while the same effector mechanisms may promote epithelial injury, mucus pathology, remodeling, fibrosis, and persistent inflammation when activation becomes excessive or poorly controlled. A major part of this functional duality is related to the ability of eosinophils to release cytotoxic granule proteins and extracellular DNA structures known as eosinophil extracellular traps (EETs). EET formation, or EETosis, does not represent a single uniform process, but includes cytolytic nuclear trap formation and rapid mitochondrial DNA (mtDNA) release without immediate cell lysis. These extracellular DNA and protein structures may help immobilize pathogens and concentrate eosinophil effector molecules at sites of mucosal or tissue inflammation. However, when EETs persist or are insufficiently cleared, they may also increase secretion viscosity, injure epithelial and stromal tissues, amplify local inflammation, and contribute to thrombotic, fibrotic, and tumor-related processes. This review summarizes the mechanisms, structural organization, inducing stimuli, protective roles, and pathogenic consequences of EETs, with particular emphasis on their relevance in infectious, allergic, airway, otologic, systemic, cutaneous, gastrointestinal, and malignant diseases.

## 2. Literature Search Strategy, Study Selection, and Evidence Assessment

A structured literature search was conducted in PubMed/MEDLINE, Scopus, and the Web of Science Core Collection from database inception to 6 July 2026. Google Scholar was used as a supplementary source to identify additional relevant publications and to perform forward citation tracking. The search was designed specifically to identify studies addressing extracellular traps, ETosis, and eosinophil extracellular trap formation (EETosis). Search terms were adapted to the syntax of each database and combined the following concepts: “eosinophil extracellular trap*”, “EET”, “EETs”, “EETosis”, “eosinophil ETosis”, “extracellular trap*”, and “ETosis”. These terms were combined, when appropriate, with keywords related to DNA origin and cellular mechanisms, including “nuclear DNA”, “mitochondrial DNA”, “mtDNA”, “cytolysis”, “degranulation”, “eosinophil granule*”, “reactive oxygen species”, “NADPH oxidase”, “PAD4”, “histone citrullination”, “galectin-10”, and “Charcot–Leyden crystal*”, as well as terms describing the infectious, inflammatory, thromboinflammatory, fibrotic, and neoplastic conditions discussed in this review. The reference lists of eligible original articles and relevant reviews were also screened manually to identify earlier or otherwise missed studies.

Eligible publications included original in vitro, ex vivo, animal, and human studies that directly investigated extracellular trap formation by eosinophils; characterized extracellular DNA structures using eosinophil-specific cellular or granule markers; examined nuclear or mitochondrial DNA release during EET formation; or evaluated the mechanistic, antimicrobial, inflammatory, clinicopathological, or prognostic relevance of EETs. Studies of extracellular traps released by other leukocyte populations were considered only when they provided a direct mechanistic comparison or a clearly relevant conceptual framework for EETosis. Review articles and systematic reviews were used to identify primary studies and to provide context, but they were not treated as primary evidence for disease-specific conclusions.

Duplicate records, conference abstracts without sufficient methodological or outcome data, editorials, opinion articles, and publications that did not provide an explicit link to extracellular traps, ETosis, or EETosis were excluded from the structured evidence set. Articles dealing only with general eosinophil development, tissue distribution, degranulation, or disease biology were not included in the trap-focused study selection unless extracellular DNA release or extracellular trap formation was directly addressed. Accordingly, the evidence synthesis and study-level appraisal were restricted to references directly related to extracellular traps, ETosis, or EETosis; broader eosinophil literature was used only where necessary to introduce biological or clinical context.

Titles and abstracts were screened for relevance, followed by full-text assessment against the predefined eligibility criteria. For each included study, the extracted information comprised the experimental or clinical setting, species and study population, EET-inducing stimulus and priming conditions, proposed nuclear or mitochondrial DNA origin, cytolytic or non-cytolytic phenotype, eosinophil-specific markers, analytical methods, implicated signaling pathways, functional outcomes, and disease relevance. Owing to substantial heterogeneity in study designs, biological samples, stimulation protocols, EET identification methods, and reported outcomes, the findings were synthesized narratively and no quantitative meta-analysis was performed.

The methodological quality and directness of the included evidence were assessed qualitatively using predefined criteria tailored to extracellular trap research. Particular weight was given to: demonstration of extracellular DNA together with eosinophil-specific cellular or granule markers; morphological or imaging confirmation of trap-like structures; adequate distinction of EETs from neutrophil extracellular traps, nonspecific necrosis, and extracellular debris; use of appropriate negative, stimulation, nuclease, or pathway-inhibition controls; clear characterization of the study population or experimental model; quantitative analysis and reproducibility; and direct support for the biological or clinical conclusion being drawn. Evidence based on multimodal identification and appropriate controls was given greater interpretative weight, whereas findings based only on circulating surrogate markers, small descriptive series, or extrapolation from other leukocyte extracellular traps were considered preliminary. This qualitative assessment was used throughout the review to distinguish established observations from mechanistic hypotheses and areas requiring further validation.

## 3. Eosinophils: Development, Tissue Distribution, and Functional Relevance in Health and Disease

Eosinophils are terminally differentiated granulocytes first recognized by Paul Ehrlich because their cytoplasmic granules showed a strong affinity for the acidic dye eosin [1,2]. These cells are morphologically defined by specific granules with a dense crystalloid core surrounded by a matrix, a structural feature that is closely related to their biological activity [3]. Eosinophils arise in the bone marrow from myeloid progenitor cells, pass through a committed eosinophil progenitor stage, and then enter the circulation as mature granulocytes [3,4]. Their differentiation, survival, and activation are controlled mainly by interleukin-3 (IL-3), interleukin-5 (IL-5), and granulocyte–macrophage colony-stimulating factor (GM-CSF) [5]. Among these signals, IL-5 has a particularly prominent role because it drives eosinophil maturation, prolongs their survival, and primes them for more intense responses to subsequent inflammatory stimulation [4,6].

Although eosinophils account for only a small proportion of circulating leukocytes, their biological importance is largely reflected in tissues rather than in peripheral blood [2]. They are present as resident or inducible immune cells in several anatomical sites, including the gastrointestinal tract, lungs, thymus, adipose tissue, endometrium, and secondary lymphoid organs [7]. This distribution indicates that eosinophils should not be viewed only as short-lived circulating effector cells, but as immune cells adapted to tissue-specific functions [7,8]. Functionally, they are strongly associated with type 2 immunity and may contribute to host defense against extracellular parasites through the release of cytotoxic granule proteins [9]. The same mechanisms, however, can become harmful in allergic inflammation and chronic eosinophilic diseases, where eosinophil-derived mediators may promote epithelial injury, mucus production, tissue remodeling, and fibrosis [10,11].

### 3.1. Physiological Roles of Eosinophils

Eosinophils have long been interpreted through the lens of cytotoxic host defense, particularly because they can release granule-derived proteins, antimicrobial peptides, reactive oxygen species, and other mediators capable of damaging extracellular pathogens and altered host tissues [9,12]. This classical view remains relevant, especially at mucosal and barrier surfaces where eosinophils are strategically positioned to respond to helminths, microbial products, and environmental stimuli [9,12]. Major basic protein (MBP), eosinophil peroxidase (EPO/EPX), eosinophil cationic protein (ECP), eosinophil-derived neurotoxin (EDN), and related effector molecules provide eosinophils with strong antimicrobial and tissue-reactive potential [13]. However, this same effector machinery illustrates the biological ambiguity of eosinophils: mechanisms that may restrict pathogens under controlled conditions can also contribute to epithelial injury, mucus hypersecretion, and local tissue damage when activation is excessive or poorly regulated [14].

The physiological role of eosinophils extends beyond direct cytotoxicity. These cells can shape local immune responses by releasing cytokines, lipid mediators, growth factors, and granule-associated molecules that influence T-cell activation and polarization, thereby connecting innate effector responses with adaptive immune regulation [15]. In tissues, eosinophils also participate in homeostatic processes, remodeling, repair, and resolution of inflammation [16]. Their behavior is influenced by extracellular matrix components, suggesting that they can sense and respond to structural changes within the tissue microenvironment. In parallel, eosinophils may support resolution through pro-resolving mediators, including resolvins, and by facilitating macrophage-dependent clearance of cellular debris and restoration of tissue integrity [17].

### 3.2. Pathological Roles of Eosinophils

Eosinophils become pathogenic when their recruitment, activation, or survival within tissues exceeds the needs of host defense or fails to resolve after the initial stimulus has disappeared. In this setting, mediators that normally participate in antimicrobial protection, barrier regulation, or repair may instead sustain inflammation and promote tissue injury [18]. This is particularly evident in allergic diseases such as asthma, allergic rhinitis, and atopic dermatitis, where eosinophilic infiltration is a consistent histological and immunological feature. In allergic tissues, sustained eosinophil activation leads to the release of cytotoxic granule proteins, oxidative mediators, cytokines, lipid mediators, and neuroimmune signals that amplify local inflammation and impair epithelial barrier integrity [19]. MBP, ECP, EPO/EPX, and EDN may directly injure epithelial cells, while interleukin-4 (IL-4), IL-5, interleukin-13 (IL-13), interleukin-31 (IL-31), nerve growth factor (NGF), and brain-derived neurotrophic factor (BDNF) may support type 2 inflammation, sensory nerve activation, mucus production, tissue remodeling, and persistence of symptoms [20]. Thus, in allergic lesions, eosinophils function as effector cells that connect type 2 inflammation with epithelial dysfunction, neuroimmune activation, mucus pathology, and remodeling.

Beyond classical allergic inflammation, eosinophils have also been implicated in non-allergic inflammatory disorders, including Crohn’s disease, chronic obstructive pulmonary disease, and non-atopic asthma [19,21]. Similar caution is needed when eosinophils are discussed in infection-related contexts [9]. Although they are most often associated with helminth-driven disease, eosinophils may also be recruited during selected bacterial, fungal, and viral infections, where antimicrobial effector programs can become harmful if they are excessive or poorly regulated [9].

### 3.3. Eosinophil Granules

Eosinophil granules are the dominant secretory organelles of mature human eosinophils and represent the main structural basis for many of their effector functions [22]. A typical eosinophil contains approximately 200 membrane-bound crystalloid granules, whose dense internal organization allows the storage of large quantities of preformed biologically active molecules [23]. This granule-rich morphology explains why eosinophils can respond rapidly once they reach inflamed or infected tissues, without depending exclusively on newly synthesized mediators [24]. The principal granule proteins include MBP, ECP, EDN, and EPX [25]. Because they also contain hydrolytic enzymes, cytokines, chemokines, and growth factors, eosinophil granules should be viewed not merely as cytotoxic storage compartments, but as immune organelles that combine antimicrobial activity with regulation of leukocyte recruitment, inflammatory signaling, remodeling, and repair [26]. Their biological effects are closely related to both charge and enzymatic activity: MBP and ECP can disturb cellular membranes and damage parasites, microorganisms, and host epithelial cells; EDN has ribonuclease activity and may support dendritic cell recruitment and antigen-specific immune responses; EPX/EPO participates in oxidative reactions that generate reactive oxidants and radical species [20,27,28]. Thus, the same properties that support antimicrobial defense can also promote collateral tissue injury when granule proteins are released excessively or retained within host tissues [14].

**Figure 1:**
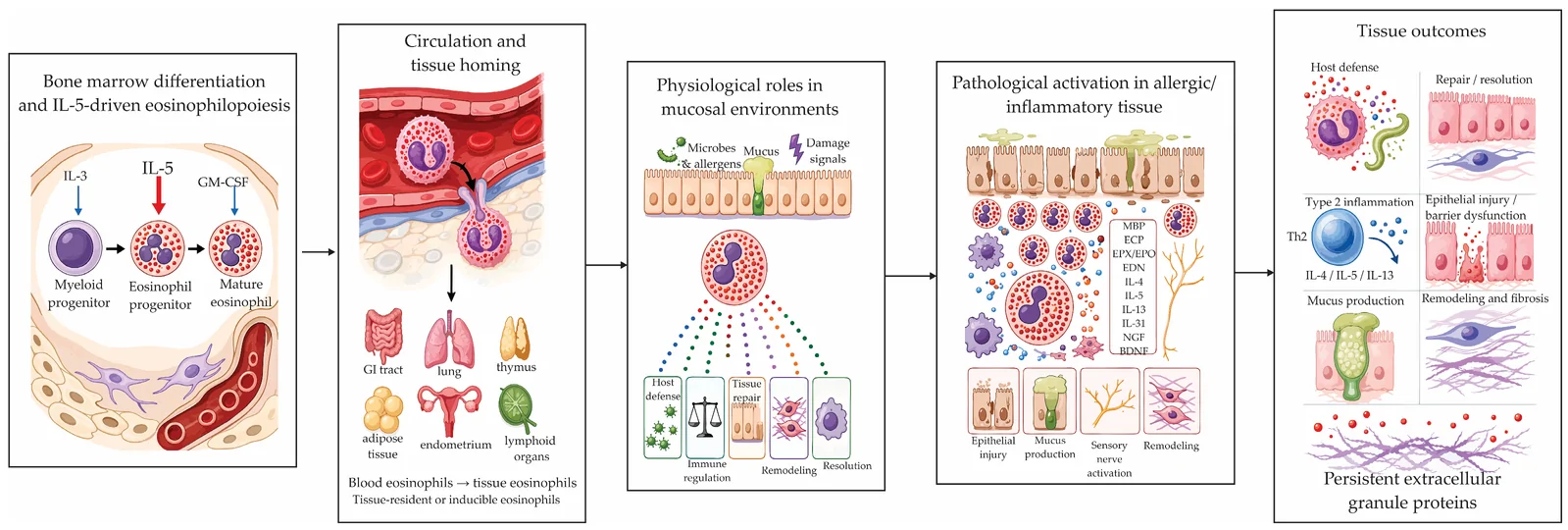
Developmental origin, tissue distribution, and granule-centered effector biology of eosinophils. This schematic summarizes the main biological transitions that define eosinophils. Eosinophils develop in the bone marrow under the influence of IL-3, IL-5, and GM-CSF, enter the circulation as mature granulocytes, and then home to several tissues, including mucosal, lymphoid, and metabolic organs. In physiological settings, eosinophils contribute to host defense, immune regulation, tissue repair, remodeling, and resolution of inflammation. However, when eosinophil recruitment or activation becomes excessive or sustained, the same granule-rich effector program may promote epithelial injury, mucus production, type 2 inflammation, sensory nerve activation, tissue remodeling, fibrosis, and persistence of extracellular granule proteins. The figure highlights eosinophil granules as the central structural and functional element linking protective and pathogenic eosinophil responses. Note: BDNF, brain-derived neurotrophic factor; ECP, eosinophil cationic protein; EDN, eosinophil-derived neurotoxin; EPO, eosinophil peroxidase; EPX, eosinophil peroxidase; GI, gastrointestinal; GM-CSF, granulocyte–macrophage colony-stimulating factor; IL, interleukin; MBP, major basic protein; NGF, nerve growth factor; Th2, T helper 2 cell.

The pathological importance of eosinophil granules is best illustrated by diseases in which eosinophil activation is accompanied by extracellular deposition of granule proteins [29]. In eosinophilic granulomatosis with polyangiitis (EGPA), granule proteins have been detected within lesional tissues, while increased serum concentrations of ECP, MBP, and EDN have been associated with active disease [30,31]. MBP is particularly relevant because it may increase epithelial permeability, induce smooth muscle contraction, and stimulate mediators involved in remodeling and fibrosis [32]. Since eosinophil granule proteins are highly cationic, they can bind to negatively charged tissue structures and remain locally retained, which may help explain why inflammation can persist even after the initial wave of eosinophil activation has subsided [33]. Free extracellular eosinophil granules have been described in asthma, including sputum and experimental models, as well as in atopic dermatitis, nasal allergy, and urticaria, supporting the idea that released granules may continue to act as independent inflammatory units outside the parent cell [34,35]. Together, these features place eosinophils on a functional continuum, from tissue homeostasis and host defense to persistent inflammation, epithelial injury, remodeling, and fibrosis, as summarized in Figure 1.

#### Release of Eosinophil Granules

Eosinophils do not release their granule-derived mediators through a single uniform mechanism. Instead, they use several secretory pathways that differ in speed, selectivity, cellular consequences, and pathological relevance [25]. These include classical exocytosis, compound exocytosis, piecemeal degranulation, and cytolysis, also referred to as lytic degranulation [2,25,36]. This distinction is important because extracellular granule proteins may result from controlled secretion by viable cells or from cytolytic disruption with release of intact granules [25,34,37].

Classical exocytosis involves the fusion of individual granules with the plasma membrane, followed by direct discharge of preformed mediators into the extracellular space [25,36]. This mechanism provides a rapid route for releasing granule proteins at sites of inflammation, although it appears to be less commonly documented in tissue specimens than more selective forms of eosinophil secretion [14]. Compound exocytosis represents a more extensive variant, in which granules first fuse with one another before connecting with the plasma membrane, thereby creating larger secretory channels and allowing a broader release of granule contents [14,36]. By contrast, piecemeal degranulation is generally considered the most frequent and physiologically adaptable form of eosinophil secretion [25,37]. During this process, selected granule components are mobilized in a controlled manner and transported toward the cell surface by eosinophil sombrero vesicles (EoSVs) [38,39]. This allows eosinophils to release specific mediators gradually, without complete granule emptying, wholesale granule fusion, or immediate loss of cell viability [37,39]. Piecemeal degranulation therefore provides a mechanism through which eosinophils can regulate local immune responses in a more selective manner while preserving cellular integrity.

**Figure 2:**
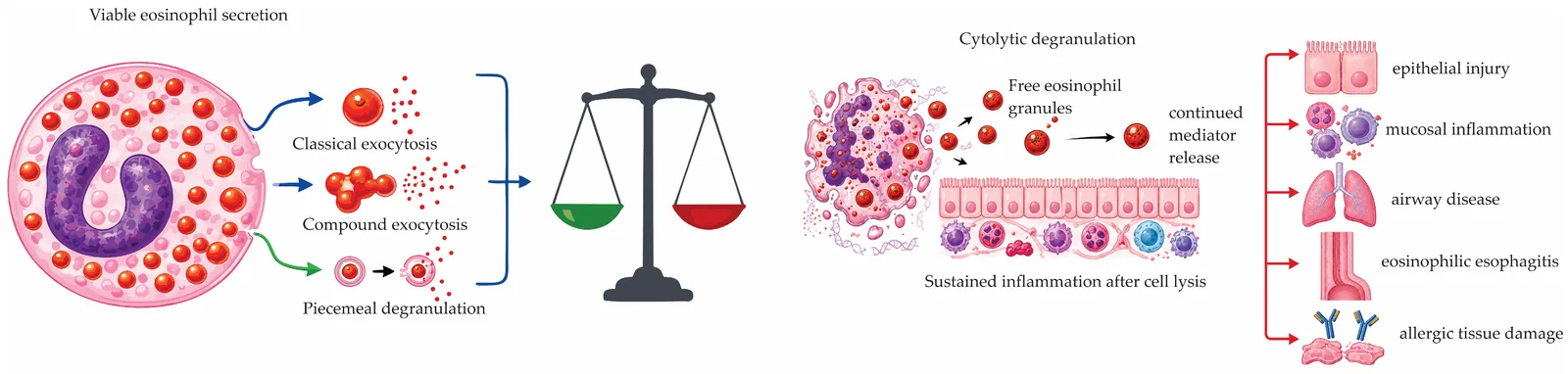
Secretory pathways and cytolytic release of eosinophil granules. Eosinophils release granule-derived mediators through four main pathways: classical exocytosis, compound exocytosis, piecemeal degranulation, and cytolysis. Classical and compound exocytosis enable rapid extracellular discharge of granule contents, whereas piecemeal degranulation supports gradual and selective mediator release while the cell remains viable. In contrast, cytolytic degranulation leads to loss of cell integrity and extracellular release of intact free eosinophil granules. Because these extracellular granules may remain functionally active, they can continue to release mediators after cell lysis and thereby sustain epithelial injury, mucosal inflammation, airway disease, eosinophilic esophagitis, and allergic tissue damage.

Cytolysis has a different biological meaning because it is associated with loss of plasma membrane integrity, nuclear disruption, and extracellular deposition of intact membrane-bound granules [25,40]. These structures, known as free eosinophil granules, may persist in tissues after eosinophil lysis [41]. They should not be regarded simply as inactive cellular debris, since free granules can retain mediator content and may remain capable of further secretion, thereby prolonging local inflammatory activity beyond the lifespan of the original cell [41]. This process has been documented across several eosinophil-rich mucosal and allergic disorders, supporting its broader pathological relevance [40]. Ultrastructural analyses of airway specimens have shown that eosinophils with chromatolysis and disrupted plasma membranes are often more prominent than apoptotic eosinophils, supporting the view that cytolytic degranulation is an active feature of airway inflammation rather than a nonspecific artifact [25,42,43]. Similar observations in eosinophilic esophagitis, where electron microscopy has demonstrated cytolysis in a substantial proportion of tissue eosinophils, indicate that intact extracellular granules may represent a persistent source of eosinophil-derived mediators in chronically inflamed mucosal tissues [44]. These different modes of granule release illustrate how eosinophils can either secrete mediators in a controlled manner while remaining viable or, after cytolysis, leave behind free granules that continue to sustain tissue inflammation, as shown in Figure 2.

Taken together, eosinophils should be understood as tissue-adapted immune cells whose biological impact depends on context, activation intensity, and the way in which their granule-derived mediators are released. In physiological settings, they contribute to host defense, immune regulation, tissue repair, and resolution of inflammation. However, when eosinophil recruitment or activation becomes excessive or persistent, the same granule-centered machinery may drive epithelial injury, mucus pathology, remodeling, fibrosis, and chronic inflammation. Importantly, eosinophil degranulation is not a single process: controlled secretion by viable cells and cytolytic release of intact extracellular granules have different pathological meanings. These key concepts are summarized in Table 1.

## 4. ETosis and Extracellular Traps as Innate Immune Mechanisms

ETosis describes a regulated immune process in which leukocytes release extracellular chromatin structures that bind antimicrobial molecules and physically restrict pathogens [45]. These extracellular traps consist mainly of decondensed chromatin, histones, and effector proteins released from the cell, and in granulocytes they are often enriched with granule-associated mediators [46]. Although extracellular trap formation was initially described in neutrophils, it is now understood as a broader innate immune mechanism involving several inflammatory cell types [47]. The morphology of ETosis differs from classical apoptosis and necrosis [48]. Apoptosis is characterized by nuclear condensation and DNA fragmentation, whereas ETosis involves chromatin relaxation, expansion, and eventual release of DNA fibers into the extracellular space. In lytic forms, rupture of the nuclear and plasma membranes enables chromatin to exit the cell and form extracellular networks [49]. Experimental triggers such as phorbol myristate acetate (PMA), microbial products, hydrogen peroxide, and respiratory burst-associated signals often link this process to reactive oxygen species generation and nicotinamide adenine dinucleotide phosphate (NADPH) oxidase activation, although the exact molecular requirements may vary between cell types and stimuli [50,51].

Extracellular trap formation serves to focus antimicrobial activity directly at sites of infection [52]. DNA fibers can immobilize extracellular microorganisms, while histones and associated effector proteins expose trapped pathogens to high local concentrations of antimicrobial or cytotoxic molecules active against bacteria, fungi, viruses, and parasites [53,54]. The presence of extracellular trap formation in both vertebrate and invertebrate immune cells suggests that this process is an evolutionarily conserved component of innate defense, rather than a specialized property of a single leukocyte lineage [55]. However, when traps are produced in excess, persist in tissues, or are not efficiently cleared, extracellular DNA and associated proteins can sustain inflammation, damage host tissues, and activate additional immune pathways [45]. Dysregulated trap formation has therefore been linked to thrombosis, appendicitis, preeclampsia, arthritis, mastitis, and cancer progression [56,57]. Thus, extracellular traps are primarily protective antimicrobial structures, but they can also contribute to inflammatory pathology when their formation or clearance is poorly controlled.

### 4.1. Eosinophil Extracellular Traps and EETosis

EETs are extracellular DNA structures released by activated eosinophils and contain eosinophil effector proteins, especially mediators stored within cytoplasmic granules [58]. The formation of EETs is often described as EETosis, but this term does not refer to a single uniform process [25]. It includes both cytolytic nuclear EETosis and the rapid release of mtDNA without cell lysis [34].

The concept of EETs was introduced by Yousefi and colleagues in 2008, several years after extracellular trap formation had been described in neutrophils [59]. Their work showed that eosinophils can release extracellular DNA fibers associated with eosinophil granule proteins, including MBP and ECP [59]. Importantly, these structures were not observed only under in vitro conditions, but were also detected in inflamed tissue samples. Early evidence was also found in colon biopsy specimens from patients with Crohn’s disease, schistosomiasis, and intestinal spirochetosis, where extracellular DNA structures associated with eosinophil granule proteins were detected in inflamed tissue [59,60]. These findings showed that extracellular trap formation is not limited to neutrophils and suggested that eosinophils can release extracellular DNA structures during inflammatory responses in tissues.

Cytolytic nuclear EETosis is characterized by a series of coordinated cellular changes. The eosinophil nucleus loses its typical lobulated shape, chromatin gradually decondenses, the nuclear membrane breaks down, and disruption of the plasma membrane allows nuclear DNA to move into the extracellular space [34,40]. The released chromatin remains associated with histones, eosinophil granule proteins, and eosinophil-derived particles, forming filamentous extracellular structures with antimicrobial and inflammatory activity [34,40]. In contrast to apoptosis, this process occurs without caspase-dependent nuclear condensation or internucleosomal DNA fragmentation [34]. It also differs from nonspecific necrosis, as these morphological changes are induced by specific stimuli and follow an organized pattern, rather than reflecting uncontrolled severe cellular injury [61].

Several stimuli have been shown to induce EETosis involving nuclear chromatin, including immunoglobulin G (IgG), immunoglobulin A (IgA), platelet-activating factor (PAF) in combination with IL-5 or GM-CSF, calcium ionophore A23187, phorbol 12-myristate 13-acetate (PMA), and autoimmune antibodies [61,62]. Experimental data show that this pathway is closely linked to NADPH oxidase activity and reactive oxygen species (ROS) generation, although its dependence on these mechanisms may vary according to the stimulus and experimental model [61]. Findings in human tissues show eosinophils with cytolysis, nuclear disruption, and extracellular granule release, supporting a close association between EETosis and cytolytic eosinophil activation in eosinophil-rich lesions [40].

Another form of trap formation involves the rapid, non-cytolytic release of mtDNA. Human eosinophils pretreated with cytokines can rapidly release mtDNA, rather than nuclear DNA, within seconds after stimulation with agonists such as lipopolysaccharide (LPS), complement component 5a (C5a), or C-C motif chemokine ligand 11 (CCL11) [59]. This response generates extracellular DNA nets without immediate rupture of the plasma membrane and without the full sequence of nuclear disintegration observed in cytolytic EETosis [63]. Mitochondrial DNA release should therefore be understood not as a milder form of terminal eosinophil death, but as a distinct mechanism of extracellular trap formation [63].

The link between cytolysis and EETosis should therefore be interpreted precisely [58]. Cytolysis provides the structural route through which nuclear chromatin, intact free granules, and granule-derived proteins can be released into the extracellular space [34]. However, EETosis should not be interpreted as passive leakage from a dying eosinophil. It is better understood as an organized effector pathway associated with cytolysis, in which nuclear and cytoplasmic remodeling culminate in the formation of extracellular DNA and protein structures [34,40]. This distinction is important because it separates EETosis from accidental cell breakdown and emphasizes that the extracellular structures formed during eosinophil activation arise through a regulated effector process [40].

In functional terms, EETs may contribute to host defense by immobilizing pathogens and locally concentrating antimicrobial proteins released from eosinophil granules within extracellular DNA networks [58]. This local accumulation of DNA, histones, and granule proteins may help limit microbial spread, especially at mucosal sites where eosinophils are frequently exposed to infectious and inflammatory stimuli [54,63,64]. However, the same structures can become harmful when they accumulate or are not efficiently cleared [58]. Stable EET aggregates may increase secretion viscosity and have been implicated in eosinophilic chronic rhinosinusitis, eosinophilic otitis, and allergic bronchopulmonary aspergillosis [54,65,66,67]. Their persistence may prolong epithelial injury, maintain local immune activation, and reinforce chronic eosinophilic inflammation [68,69]. Thus, EETs appear to contribute to pathology primarily through persistent DNA and granule protein aggregates that increase secretion viscosity and sustain local tissue inflammation.

### 4.2. Structural Organization of Eosinophil Extracellular Traps

Structurally, EETs should not be regarded as a uniform entity, because their composition depends on how the trap is formed [70]. Their basic architecture consists of an extracellular DNA network that can bind pathogens, allergens, inflammatory particles, and cell-derived proteins [71]. Nuclear EETs are rich in chromatin and contain histones, whereas mtDNA-based EETs are generally histone-negative [70]. ETs may contain MBP, ECP, EPX, EDN, and galectin-10, also known as Charcot–Leyden crystal protein [20,71,72]. The DNA network enables pathogen immobilization, while proteins released from eosinophil granules contribute cytotoxic, ribonuclease, oxidative, and immunomodulatory effects [20,71]. Extracellular histones, when present, may strengthen antimicrobial effects, but they can also damage host cells and activate inflammatory pathways [73]. In this context, EET-derived DNA is more than a structural component; when traps persist in chronically inflamed tissues, it may also act as a damage-associated molecular pattern capable of sustaining innate immune activation [58,74].

A distinctive feature of EETs, compared with many neutrophil extracellular traps (NETs), is their close association with intact free eosinophil granules, either as individual granules or in clusters [34]. This is biologically important because free eosinophil granules are not merely inert remnants of cell disruption. They can retain secretory capacity, express functional receptors for cytokines, chemokines, and eicosanoids, and, after stimulation, release selected mediators such as eosinophil cationic protein, EPX, IL-4, and interleukin-6 (IL-6) [41,75]. EETs are therefore best viewed as multicomponent extracellular structures in which DNA fibers, histones, intact granules, and granule-derived proteins together shape antimicrobial and inflammatory activity [54]. Their physical behavior also differs from the simple release of soluble mediators. In fluid environments, including blood, exudates, mucus, and airway secretions, eosinophil DNA traps can form adhesive filamentous networks that attach to cells, pathogens, debris, and nearby surfaces [54]. This behavior is influenced by shear forces, hydrophobic interactions, surface-active molecules, and enzymatic degradation by nucleases and proteases [54]. Compared with many NETs, EETs often contain thicker and more condensed chromatin fibers, approximately 25–35 nm in diameter, which may contribute to lower pliability, greater resistance to deoxyribonuclease I (DNase I), and longer extracellular persistence [62,65]. This stability may help explain why eosinophil-rich secretions in airway disease can become highly viscous, difficult to clear, and capable of sustaining epithelial injury and local inflammation even after the initial eosinophil activation has passed.

### 4.3. Induction of Eosinophil Extracellular Trap Formation

EETosis can be triggered by a wide range of stimuli, suggesting that eosinophils do not depend on a single activation pathway to form extracellular traps [70]. Many experimental findings fit a two-step model, in which cytokine priming by IL-5, interferon-γ (IFN-γ), or GM-CSF makes eosinophils more responsive to secondary receptor-mediated signals [34,59,76]. These secondary signals may involve Toll-like receptors (TLRs), complement receptors, chemokine receptors, Fc receptors, or adhesion-related pathways [59,62,76]. In vitro studies have shown that eosinophils pretreated with IL-5 or IFN-γ can release EETs after stimulation with C5a, LPS, or CCL11, suggesting that tissue eosinophilia may create conditions for rapid EET formation in response to microbial or inflammatory signals [59,76]. Microbial stimuli such as *Staphylococcus aureus*, *Aspergillus fumigatus*, and respiratory syncytial virus further indicate that bacterial, fungal, and viral signals can all contribute to EET induction [64,77,78]. Immune complex-mediated activation may also be relevant, as immobilized IgG and IgA can induce EETosis, probably through signaling mediated by Fc receptors [34]. This mechanism may be especially important at mucosal surfaces, where IgA-dependent recognition may promote eosinophil extracellular responses as part of barrier immunity [62]. Experimental activators such as PAF, PMA, and calcium ionophore A23187 remain useful for studying EETosis mechanisms, although strong pharmacological stimulation may not fully reflect the sequence of signals that occurs in inflamed human tissues [34,40].

ROS are among the best characterized upstream signals in EETosis and may contribute directly to extracellular trap formation, rather than merely reflecting secondary oxidative inflammation [79]. The role for NADPH oxidase is supported by experiments showing that diphenyleneiodonium (DPI) and antioxidant treatment with N-acetylcysteine (NAC) can reduce EET formation, including in models of asthma induced by ovalbumin (OVA) [62,79]. Many EET-inducing stimuli involve ROS signaling, but the importance of this pathway may vary depending on the stimulus, eosinophil priming, and the tissue environment [62]. Epithelial-derived signals may further influence this process. Thymic stromal lymphopoietin (TSLP) is released at barrier surfaces after epithelial injury or environmental stimulation and is increased in several type 2 inflammatory diseases. Since eosinophils express the TSLP receptor (TSLPR)/interleukin-7 receptor alpha (IL-7Rα) receptor complex, TSLP can directly activate eosinophils, favor their accumulation in tissues, and promote EET formation, thereby connecting epithelial barrier injury with extracellular eosinophilic inflammation [80].

EETosis may also be triggered by sterile and metabolic stimuli. Monosodium urate crystals (MSU) can induce eosinophil trap formation, suggesting a possible role in crystal-associated autoinflammation [81]. Long-chain fatty acids, both saturated and unsaturated, can serve as metabolic substrates for eosinophils and promote filamentous extracellular DNA release, suggesting a connection between lipid metabolism and eosinophil activation [82]. Lysophosphatidylserine (LysoPS) provides another example of a lipid mediator capable of inducing extensive EET formation, together with cytolysis and degranulation [83]. Unlike several classical ROS-dependent pathways, LysoPS-induced EETosis appears to occur independently of ROS production and instead involves peptidyl arginine deiminase 4 (PAD4)-dependent histone citrullination and signaling through the P2Y10 receptor [83]. The finding that eosinophils from patients with severe asthma may show higher surface expression of P2Y10 and stronger degranulation after LysoPS stimulation supports the possibility that lipid signals contribute to EET responses in specific disease settings. However, their relative importance in vivo remains to be clarified [83]. The principal experimental and disease-associated stimuli reported to induce EET formation, together with the species studied, priming conditions, DNA origin, ROS and PAD4 requirements, and main experimental readouts, are summarized in Table 2.

**Figure 3:**
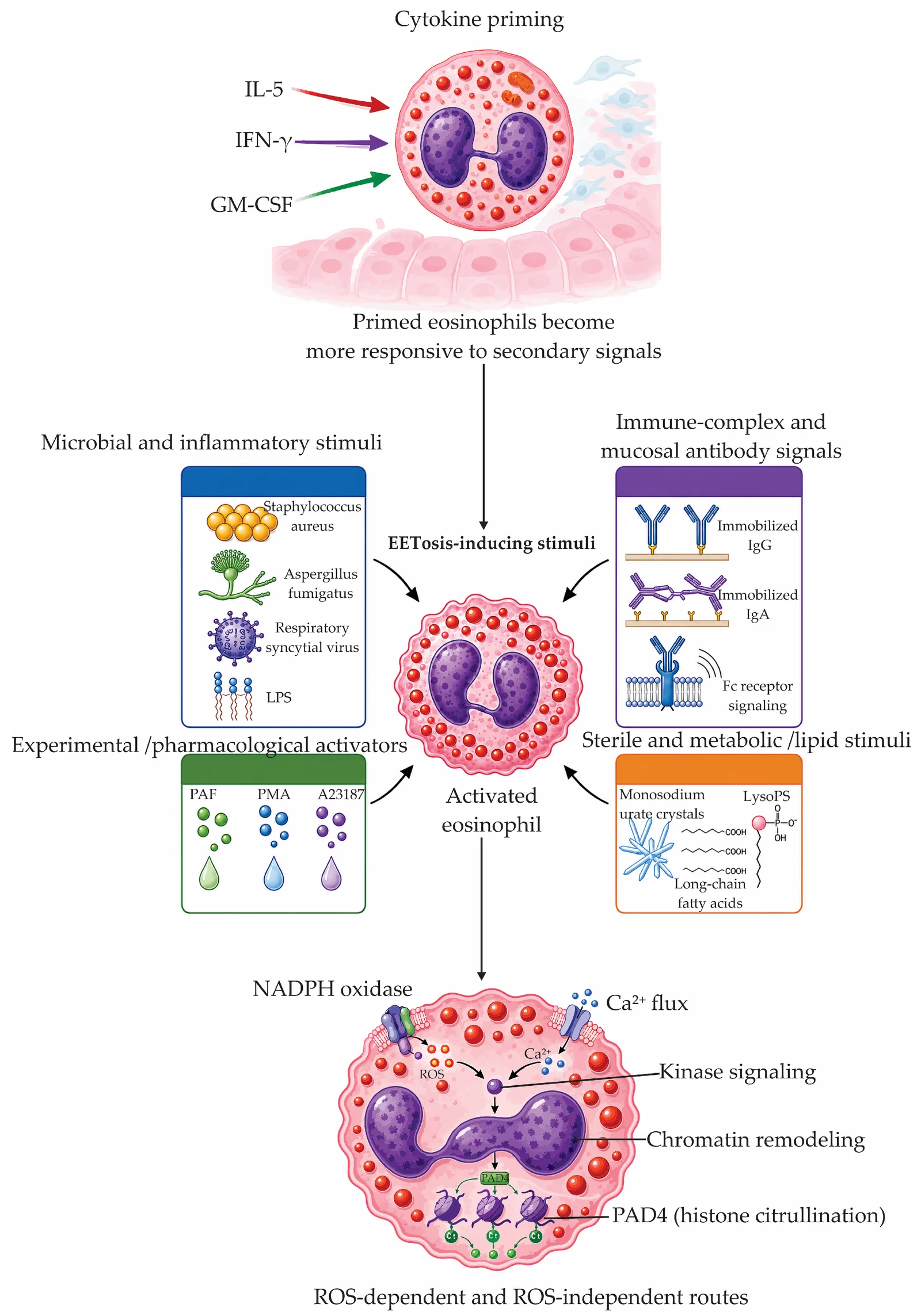
Stimuli and upstream signaling pathways involved in EETosis induction. EETosis may be initiated by a broad range of signals rather than by a single uniform trigger. Cytokine priming, particularly by IL-5, IFN-γ, and GM-CSF, prepares eosinophils to respond more strongly to secondary stimuli. These secondary inputs include microbial and inflammatory signals, immune-complex and mucosal antibody signals, sterile and metabolic or lipid stimuli, and experimental pharmacological activators. After activation, these pathways converge on intracellular programs that may involve NADPH oxidase, ROS generation, Ca^2+^ flux, kinase signaling, chromatin remodeling, and, in selected settings, PAD4-dependent histone citrullination. The figure emphasizes that EET induction can proceed through both ROS-dependent and ROS-independent routes, helping explain why eosinophil extracellular trap formation can occur in response to diverse inflammatory environments. Note: A23187, calcium ionophore A23187; Ca^2+^, calcium; EETosis, eosinophil extracellular trap formation; Fc, fragment crystallizable region; GM-CSF, granulocyte–macrophage colony-stimulating factor; IFN-γ, interferon-γ; IgA, immunoglobulin A; IgG, immunoglobulin G; IL-5, interleukin-5; LPS, lipopolysaccharide; LysoPS, lysophosphatidylserine; NADPH, nicotinamide adenine dinucleotide phosphate; PAD4, peptidyl arginine deiminase 4; PAF, platelet-activating factor; PMA, phorbol 12-myristate 13-acetate; ROS, reactive oxygen species.

Together, these findings indicate that EETosis is induced through a two-step process in which cytokine priming increases eosinophil responsiveness to diverse secondary stimuli that converge on intracellular signaling pathways leading to extracellular trap formation, as summarized in Figure 3.

### 4.4. Mechanistic Sequence of EETosis

The mechanisms of EETosis can be discussed in comparison with NETosis, but EETosis should not be viewed simply as the neutrophil process reproduced in eosinophils [62]. In neutrophils, extracellular trap formation is generally described as a regulated process initiated by receptor activation and downstream ROS-dependent or ROS-independent signaling. These signals may lead to calcium-dependent activation of chromatin-modifying enzymes such as PAD4, chromatin decondensation, disruption of the nuclear envelope, and, in lytic forms, rupture of the plasma membrane followed by extracellular DNA release [84]. Eosinophils share some of these general features, especially chromatin decondensation and extracellular DNA release, but they also have distinct characteristics related to their bilobed nucleus, abundant crystalloid granules, mtDNA release, and frequent extracellular deposition of intact free granules [34,40,59]. For this reason, EETosis is better described as a group of partly overlapping pathways rather than as one fixed linear sequence. After priming and receptor activation, eosinophils may follow either a cytolytic nuclear pathway or a mitochondrial pathway. The cytolytic pathway involves nuclear remodeling, chromatin decondensation, membrane rupture, and release of DNA associated with histones. In contrast, the mitochondrial pathway allows extracellular DNA nets to form without immediate loss of plasma membrane integrity [34,40,59].

The early signaling phase translates extracellular stimulation into intracellular programs that prepare eosinophils for DNA release [34]. Depending on the stimulus, this phase may involve NADPH oxidase activation, ROS generation, intracellular calcium flux, kinase signaling, and pathways that modify chromatin structure [34,62]. Several experimental models support a role for ROS signaling in EETosis, but ROS should not be considered a universal requirement for extracellular trap formation [79,85]. Some stimuli, including *Aspergillus fumigatus* and LysoPS, can induce EET formation through ROS-independent mechanisms, indicating that similar extracellular trap morphology may result from different intracellular routes [77,83]. This variability is important because it indicates that EETosis is regulated through multiple, partly overlapping activation pathways, which may be shaped by the local inflammatory environment.

Chromatin remodeling is a central intracellular step in nuclear-derived EETosis [86,87]. Human eosinophils express PAD4, a calcium-dependent enzyme that can mediate histone citrullination [88]. PAD4 appears to be required in at least some forms of EETosis, particularly LysoPS-induced trap formation, where histone citrullination has been linked to extracellular DNA release [83]. Its role in other settings, including EETosis induced by PMA, immune complexes, PAF, or MSU, remains less well defined [54,63,81]. PAD4 should therefore be described as an important enzyme involved in chromatin remodeling, but not as an obligatory mediator of all EETosis pathways.

In cytolytic nuclear EETosis, early morphological events are dominated by nuclear remodeling [40]. The lobulated eosinophil nucleus loses its segmented structure, chromatin gradually decondenses, the distinction between euchromatin and heterochromatin becomes less clear, the nucleus rounds and enlarges, and the nuclear envelope becomes unstable before chromatin moves into the cytoplasm [34,40]. The late cytolytic phase is characterized by plasma membrane rupture and the release of decondensed chromatin into the extracellular space, where it forms extracellular fibers containing DNA and histones [34,54]. This sequence should not be interpreted as passive release of cellular contents during cell death. Rather, cytolytic EETosis represents an organized form of eosinophil cytolysis in which nuclear remodeling, membrane disruption, granule release, and extracellular chromatin deposition lead to the formation of inflammatory DNA and protein networks [40].

A separate sequence of events seems to characterize noncytolytic mitochondrial EET release. In this pathway, eosinophils do not first undergo nuclear collapse [58,59]. Instead, eosinophils exposed to cytokines can rapidly release mtDNA after stimulation, while initially preserving plasma membrane integrity [59]. Recent kinetic observations suggest that mitochondrial EET formation may occur through a stepwise extracellular process: eosinophils first release granule proteins, particularly EPX, followed later by mtDNA release [89]. The extracellular DNA network and granule proteins may then associate outside the cell, rather than being fully assembled before release [89]. This raises unresolved questions about whether granule proteins bind mtDNA immediately after its release, whether they are deposited first and later incorporated into the DNA network, or whether different eosinophils provide separate components of the final extracellular trap [89].

The extracellular phase of EETosis is strongly shaped by eosinophil-specific organelles [34]. In cytolytic EETosis, extracellular chromatin deposition is accompanied by the release of intact or nearly intact free eosinophil granules [34]. Free EoSVs may also be found near extracellular chromatin and granules, suggesting that vesicular structures can accompany EETosis and may participate in mediator transport or local inflammatory signaling [39]. Charcot–Leyden crystals, composed mainly of galectin-10, are often located close to extracellular granules, externalized chromatin, and vesicular structures. Their presence supports the interpretation that EETosis represents more than extracellular DNA fiber formation and may be accompanied by eosinophil cytolysis, degranulation, vesicle release, and galectin-10 crystallization.

Current understanding of EETosis is based mainly on in vitro experiments using isolated eosinophils, supported by a smaller number of animal studies and descriptive analyses of human tissues. These levels of evidence should not be considered equivalent. In vitro studies allow detailed investigation of signaling pathways but often use cytokine priming, strong stimuli, and experimental conditions that may not reflect the tissue environment. Animal models provide evidence of EET formation in vivo, although species-specific differences may limit translation to human disease. Human studies confirm the presence of EET-associated structures in several eosinophilic disorders, but most are small, cross-sectional, and unable to establish causality.

Interpretation is further complicated by substantial methodological heterogeneity. Studies differ in stimuli, incubation periods, detection methods, definitions of EET formation, and approaches used to distinguish nuclear from mitochondrial DNA. In addition, extracellular DNA, histone citrullination, and colocalization with granule proteins are not interchangeable measures of EETosis. Consequently, apparently conflicting findings regarding ROS, PAD4, and cell viability may reflect both stimulus-dependent biology and methodological differences. Most available evidence therefore remains preclinical, and clinical validation is limited.

**Figure 4:**
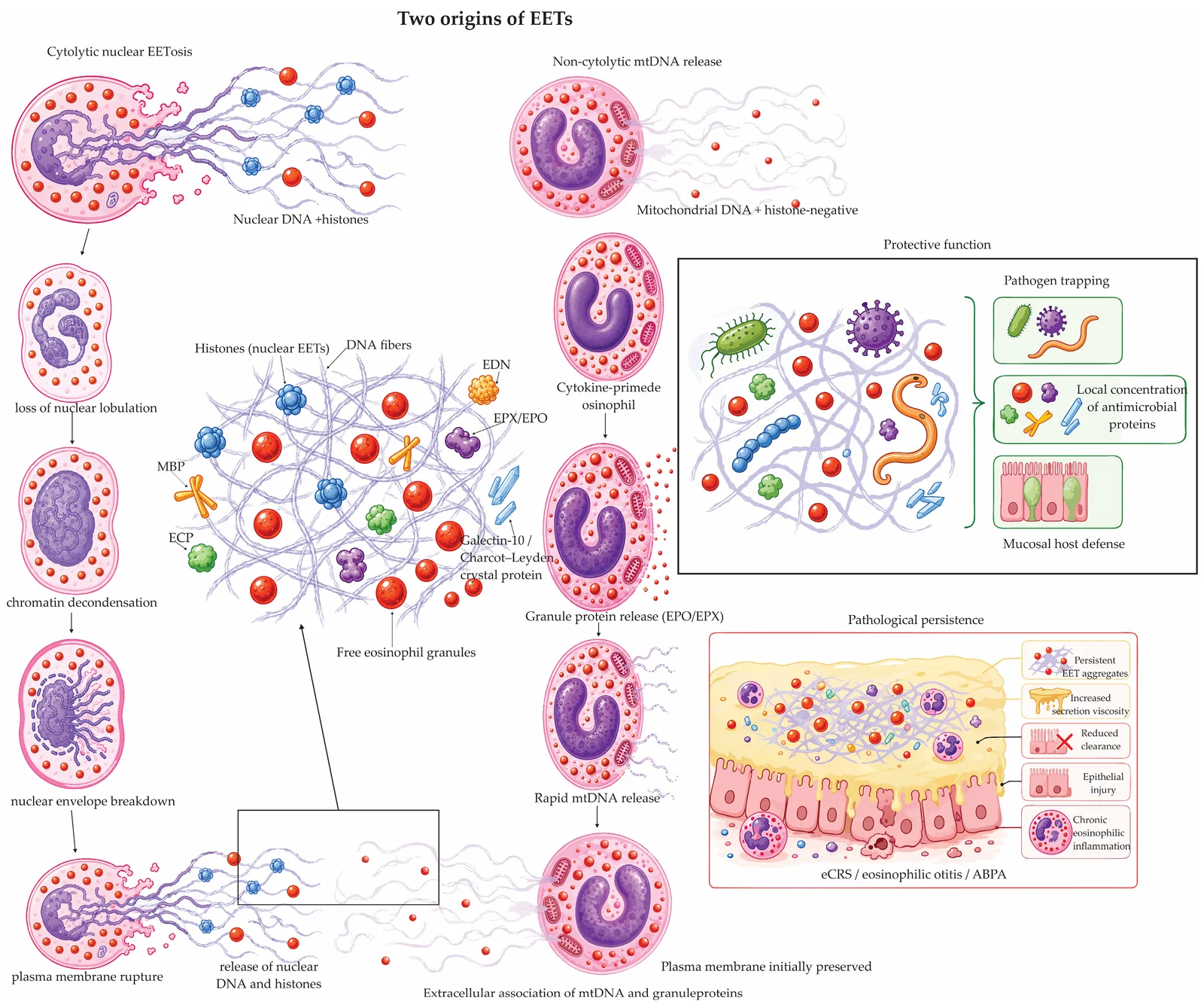
Nuclear and mitochondrial origins of eosinophil extracellular traps. Eosinophil extracellular traps may arise through two main routes. In cytolytic nuclear EETosis, the eosinophil nucleus loses its lobulated structure, chromatin decondenses, the nuclear envelope breaks down, and plasma membrane rupture allows nuclear DNA and histones to form extracellular fibers decorated with granule proteins and free eosinophil granules. In non-cytolytic mitochondrial DNA release, cytokine-primed eosinophils initially preserve plasma membrane integrity while rapidly releasing mtDNA, which can associate extracellularly with granule-derived proteins. These DNA–protein networks may support mucosal host defense by trapping pathogens and locally concentrating antimicrobial mediators. When they persist or are poorly cleared, however, EET aggregates may increase secretion viscosity, impair clearance, injure epithelium, and sustain chronic eosinophilic inflammation in diseases such as eCRS, eosinophilic otitis, and ABPA. Note: ABPA, allergic bronchopulmonary aspergillosis; CCL11, C-C motif chemokine ligand 11/eotaxin-1; ECP, eosinophil cationic protein; eCRS, eosinophilic chronic rhinosinusitis; EDN, eosinophil-derived neurotoxin; EETs, eosinophil extracellular traps; EPO, eosinophil peroxidase; EPX, eosinophil peroxidase; MBP, major basic protein; mtDNA, mitochondrial DNA.

Overall, EETosis is best understood as a set of related but distinct eosinophil effector pathways, in which nuclear chromatin or mtDNA can generate extracellular DNA–protein networks with both protective and pathological consequences, as summarized in Figure 4.

## 5. Protective Roles of Eosinophil Extracellular Traps

The protective role of EETs is better described as limiting pathogen spread in the extracellular space, rather than as a uniform mechanism of direct pathogen killing [58]. Through EET formation, eosinophils can extend their defensive activity beyond classical degranulation by releasing extracellular DNA networks that immobilize pathogens and concentrate eosinophil effector molecules at sites of mucosal or tissue inflammation [59]. This mechanism may be especially relevant when pathogens are too large, too numerous, attached to epithelial surfaces, or located in extracellular sites where phagocytic clearance is inefficient [9,63,77]. In this setting, EETs may contribute to local defense by using extracellular DNA to immobilize pathogens. Granule mediators, including MBP, ECP, EPX, and EDN, may further contribute cytotoxic, oxidative, ribonuclease-related, and immunomodulatory activity [20,87].

The biological consequences of EET formation are likely to vary with pathogen structure and the local microenvironment [70]. In bacterial infection, EETs may help restrict bacterial spread by trapping organisms and exposing them to concentrated granule-derived mediators [59]. Gram-positive bacteria, with their thick peptidoglycan-rich cell walls and surface adhesins, may be particularly relevant at damaged epithelial barriers, where direct bacterial contact, colonization, and local tissue retention can promote eosinophil activation [64]. Gram-negative bacteria provide different stimuli, including LPS and complement- or antibody-mediated recognition, and may induce EET-related antibacterial activity, especially when eosinophils are cytokine-primed or when bacteria are opsonized [34,59]. Differences between aerobic, facultative anaerobic, and anaerobic organisms are less clearly defined in the EET literature, but they remain conceptually important because oxygen availability, oxidative burst activity, biofilm formation, and tissue hypoxia may influence whether EETs mainly immobilize bacteria, increase local mediator concentration, or cooperate with other antimicrobial mechanisms [12].

Compared with bacteria, fungi and parasites present distinct structural and immunological features that may influence how eosinophils respond to them [63]. Fungal elements are often larger and structurally more complex, with cell-wall components such as β-glucans that can link pathogen recognition to eosinophil adhesion, degranulation, and extracellular trap formation [77,90]. In this setting, EETs may contribute more to limiting fungal spread than to direct fungal killing, particularly when fungal material is retained within mucus or inflamed airway secretions [77]. Parasites, especially helminths and their larval stages, represent even larger extracellular targets that cannot be cleared by conventional phagocytosis [91]. For these organisms, the likely protective value of EETosis lies in immobilization, local concentration of toxic eosinophil mediators, and amplification of antiparasitic inflammation, although direct parasite killing by EETs remains less firmly established [92]. Thus, across bacteria, fungi, and parasites, the common defensive principle of EETs is extracellular containment, whereas the final outcome depends on pathogen size, surface structure, oxygen availability, epithelial integrity, eosinophil priming, and cooperation with other innate immune cells.

### 5.1. EETs in Bacterial Infections

Bacterial stimulation provides one of the clearest experimental models for examining EETs as extracellular antibacterial structures [59,70]. Cytokine-primed eosinophils become more responsive to secondary bacterial signals and can release extracellular traps after contact with Gram-negative bacteria, including *Escherichia coli* (*E. coli*), or Gram-positive bacteria such as *Staphylococcus aureus* (*S. aureus*) [64,87]. This distinction is biologically relevant because Gram-negative organisms can stimulate eosinophils through LPS and through recognition involving complement or antibodies [59]. Gram-positive bacteria, by contrast, may promote eosinophil activation through direct surface contact, adhesins, toxins, or colonization of injured epithelial barriers [64,93]. In the model of vital EET formation, primed eosinophils rapidly release mtDNA in a ROS-dependent manner without immediate loss of cell viability. The extracellular DNA then associates with eosinophil granule proteins and forms antibacterial DNA–protein networks capable of binding bacteria and contributing to bacterial killing independently of classical phagocytosis [54,94]. This mechanism may be especially relevant when bacteria are extracellular, opsonized, adherent to epithelial surfaces, or positioned in sites where phagocytic clearance is limited [64]. In contrast, some bacteria, particularly S. aureus, may also induce cytolytic EET release, suggesting that bacterial virulence factors and pathogen-specific properties can influence whether eosinophils undergo vital mtDNA release or lytic nuclear trap formation [64]. The antibacterial effect of EETs should nevertheless be interpreted cautiously. Experimental studies support bacterial trapping and DNA-dependent antibacterial activity, but in infected tissues it is difficult to separate the contribution of EETs from degranulation, soluble granule proteins, ROS, complement, antibodies, epithelial responses, and cooperation with neutrophils or macrophages [34]. Therefore, bacterial EETosis is best presented as a mechanism that may restrict bacterial spread and increase local antimicrobial pressure, rather than as an isolated or universally dominant pathway of bacterial killing.

#### 5.1.1. EETs in *Staphylococcus aureus* Infection

*S. aureus* appears to be one of the strongest bacterial triggers of EET formation in eosinophil-rich airway disease, particularly in chronic rhinosinusitis with nasal polyps (CRSwNP) [60,64,95]. In ex vivo air–liquid interface (ALI) culture of nasal polyp tissue, *S. aureus* exposure induces rapid EET formation [64]. Within the first hour, extracellular traps can be detected, and eosinophils undergoing EETosis are found mainly in areas where bacteria are present [64]. The preferential appearance of EETs at bacteria-containing tissue sites suggests that EETosis is not merely a nonspecific consequence of eosinophil activation, but a localized response to bacterial contact within diseased sinonasal mucosa [64,67]. Eosinophils also appear capable of migrating toward *S. aureus*, especially when bacteria are present together with epithelial cells, indicating that epithelial–bacterial interactions may generate chemotactic signals that direct eosinophils toward damaged epithelial areas [64]. In CRSwNP, this mechanism is particularly relevant at epithelial defects, where bacterial colonization can bring *S. aureus* into direct contact with subepithelial eosinophils [64]. Mechanistically, *S. aureus*-induced EETosis is closely linked to oxidative activation. Bacterial exposure rapidly increases ROS production, while NADPH oxidase inhibition reduces both ROS generation and trap release, supporting the role of NADPH oxidase-derived ROS in this response [59,64]. Functionally, EETs may entrap *S. aureus* within extracellular DNA networks and concentrate eosinophil-derived antimicrobial mediators at damaged epithelial surfaces, providing a secondary defense mechanism when epithelial integrity is compromised [64]. However, persistent or excessive EET formation may also promote mucus thickening, epithelial injury, and chronic tissue inflammation because EETs contain extracellular DNA and cytotoxic granule proteins [64,67]. The relevance of this mechanism may extend beyond sinonasal disease, although the evidence is less direct. In atopic dermatitis, *S. aureus* colonization is common and is associated with greater disease severity. EETosis may therefore contribute to eosinophilic skin inflammation, possibly through IL-5 and other type 2 inflammatory signals that support eosinophil survival, activation, and EET formation [96,97]. In systemic *S. aureus* infection, emerging data suggest that type 2 immune components and eosinophil activity may influence host defense, but the direct contribution of EETs remains insufficiently defined [98]. Therefore, *S. aureus*-induced EETosis is best presented as a localized antibacterial response that may support mucosal defense at damaged epithelial surfaces, while also contributing to inflammatory persistence when bacterial colonization and eosinophil activation become chronic. The evidence for *S. aureus*-induced EETosis is strengthened by the use of human sinonasal tissue, in which bacterial exposure increased EET formation 4.2 ± 0.9-fold [64]. In a separate cross-sectional study of 43 patients with chronic rhinosinusitis, tissue EET counts correlated with the Lund–Mackay CT score (*r* = 0.51, *p* = 0.009) and inversely with olfactory function (*r* = −0.65, *p* < 0.001), but were also strongly related to the number of infiltrating eosinophils (*r* = 0.83, *p* < 0.001) [67]. These findings demonstrate an association with disease severity but do not establish that *S. aureus*-induced EETs independently cause tissue damage or predict exacerbations, postoperative recurrence, or treatment response.

#### 5.1.2. EETs in *Staphylococcus epidermidis*-Related Barrier Responses

*Staphylococcus epidermidis* is usually regarded as a commensal skin bacterium and an important component of the cutaneous microbiota; however, disruption of the skin barrier may allow it to interact with deeper tissue compartments and trigger local immune activation [99]. Unlike strongly pro-inflammatory bacteria such as Staphylococcus aureus, *S. epidermidis* may not consistently induce robust EET formation by itself. This suggests that additional signals released during epithelial barrier injury may be needed to enhance eosinophil antibacterial activity [80]. Thymic stromal lymphopoietin (TSLP) may provide such a signal, as barrier injury can induce TSLP release from keratinocytes [80]. TSLP may then activate eosinophils and enhance their antibacterial activity against *S. epidermidis*, possibly through eosinophil extracellular trap (EET) formation or granule mediator release [80]. This response may support barrier defense by limiting the spread of bacteria after tissue injury. However, it should be interpreted cautiously in atopic dermatitis and other type 2-dominated skin diseases, where the skin microbiota is already disturbed [80,100]. Excessive suppression of S. epidermidis by eosinophils could theoretically weaken the protective role of commensal bacteria and indirectly allow greater S. aureus colonization, which is common in atopic skin inflammation [100]. TSLP-induced eosinophil responses to *S. epidermidis* should therefore be presented as a barrier defense response with potential protective effects. However, when excessive or insufficiently regulated, this response may contribute to microbiome imbalance and persistent type 2 inflammation. Furthermore, the evidence for this pathway is limited mainly to in vitro experiments with isolated human eosinophils. Although TSLP-dependent EET formation and antibacterial activity against *S. epidermidis* were demonstrated experimentally, there is no direct clinical evidence that this response alters the skin microbiome, protects the epithelial barrier, or promotes *S. aureus* colonization in patients. The proposed effects on microbiome imbalance should therefore be regarded as a hypothesis rather than an established disease mechanism.

#### 5.1.3. EETs in *Escherichia coli* Infection

*Escherichia coli* is a Gram-negative bacterium that normally belongs to the intestinal microbiota, but pathogenic strains can cause intestinal, urinary, bloodstream, or intra-abdominal infections, especially when mucosal barriers are disrupted or host defenses are weakened [101]. In experimental settings, activated eosinophils can respond to opsonized *E. coli* by releasing EETs with measurable antibacterial activity [59]. This response is biologically relevant because it appears to rely on mechanisms distinct from classical phagocytosis. Activated eosinophils killed approximately 90% of opsonized *E. coli* within 45 min, and this effect was almost completely abolished by DNase but not by inhibition of phagocytosis with cytochalasin D [58,59]. These findings provide direct experimental evidence of DNA-dependent antibacterial activity. However, they were obtained using isolated eosinophils under controlled in vitro conditions, and no human clinical study has demonstrated that EET burden is associated with bacterial clearance, infection severity, organ dysfunction, or treatment outcome.

### 5.2. EETs in Parasitic Infections

Parasitic infections, particularly those caused by helminths, are a classical setting in which eosinophils have been viewed as important effector cells [102]. Because many helminths and larval stages are too large to be efficiently phagocytosed, eosinophil responses are usually based on mediator release at the parasite surface, local inflammation, and parasite immobilization [91]. The contribution of EETosis to antihelminth responses remains insufficiently defined. Earlier studies using *Haemonchus contortus* larvae suggested that eosinophils can release extracellular DNA structures capable of trapping larvae, providing early evidence that EET formation may contribute to parasite immobilization [103]. However, these findings should be interpreted cautiously, because limited eosinophil purity in some preparations makes it difficult to exclude contributions from other granulocyte populations. More recent studies have provided clearer mechanistic evidence that helminth-derived stimuli can induce EETosis. Microfilariae and infective L3 larvae of Litomosoides sigmodontis, as well as microfilariae of Dirofilaria immitis, can induce suicidal EETosis in murine and human eosinophils through a pathway dependent on Dectin-1 [92]. These findings indicate that pattern recognition receptor signaling, including Dectin-1 signaling, contributes to EET formation after helminth stimulation and may represent one component of eosinophil antihelminth responses [92]. Nevertheless, the functional outcome remains uncertain. Parasite-induced EETs may immobilize parasite forms and concentrate eosinophil mediators near their surface, but it is still unclear whether they directly contribute to parasite killing in vivo or mainly shape the inflammatory microenvironment around the parasite. The evidence for parasite-induced EETosis remains predominantly preclinical. The earlier *H. contortus* study used leukocyte preparations containing only approximately 30% eosinophils, limiting attribution of the observed traps specifically to eosinophils [103]. More recent experiments demonstrated Dectin-1-dependent EET formation, reduced microfilarial motility in vitro, and accelerated parasite clearance in mice [92], but these findings have not been validated in infected patients. No clinical study has yet linked EET burden with parasite load, tissue injury, symptom severity, or response to antiparasitic treatment.

### 5.3. EETs in Fungal Infections

Fungi are important in eosinophil biology because they can act both as invasive pathogens and as environmental allergens that promote type 2 inflammation [104]. Eosinophils can recognize fungal molecular patterns and respond through adhesion, degranulation, cytotoxic mediator release, and, in selected settings, extracellular trap formation [90]. β-glucans are among the fungal cell-wall components most clearly linked to eosinophil activation, acting at least partly through the β2-integrin macrophage-1 antigen (Mac-1; CD11b/CD18) [90]. This pathway may connect fungal recognition with eosinophil adhesion, degranulation, and possible EET formation [90,105]. Experimental evidence also supports eosinophil antifungal activity, including CD11b involvement in responses to *Alternaria alternata* and cytotoxic granule protein release with antifungal effects [90,106]. However, evidence that EETs themselves represent a major mechanism of antifungal killing remains limited. EETs have been described in allergic bronchopulmonary aspergillosis, while in vitro studies using *Candida albicans* have shown that EETs can capture fungal cells [54,65].

*Aspergillus fumigatus* deserves specific attention because it can act both as an opportunistic pathogen and as a potent aeroallergen, particularly in allergic bronchopulmonary aspergillosis, severe asthma, and selected forms of chronic rhinosinusitis [104,107]. In allergic bronchopulmonary aspergillosis (ABPA), repeated exposure to *Aspergillus* antigens, airway colonization, epithelial barrier dysfunction, mucus retention, and type 2 eosinophilic inflammation create conditions that favor EET formation [65,66]. Clinical bronchial mucus samples from patients with ABPA and microbiologically confirmed *A. fumigatus* contain abundant EETs within highly viscous secretions [65,66]. These traps appear to be predominantly of nuclear origin, as extracellular DNA colocalizes with histone H1 and citrullinated histone H3 [65]. In vitro exposure of human eosinophils to *A. fumigatus* conidia can induce cytolytic EETosis, as shown by increased lactate dehydrogenase (LDH) activity and loss of plasma membrane integrity [66,77]. At the signaling level, *A. fumigatus*-induced EETosis appears to follow a pathway distinct from several classical ROS-dependent forms of extracellular trap formation. Available evidence suggests that this response does not primarily require nicotinamide adenine dinucleotide phosphate oxidase, more commonly referred to as NADPH oxidase (NOX), or PAD4. Instead, fungal sensing appears to be initiated through Mac-1/CD11b and then transmitted through spleen tyrosine kinase (Syk), Src-family kinases (SFKs), Akt/protein kinase B (Akt/PKB), calcium flux, and p38 mitogen-activated protein kinase (p38 MAPK), thereby linking fungal cell-wall recognition with cytolytic EET release [66,108]. Regarding their biological role, *A. fumigatus*-induced EETs can bind fungal conidia and immobilize them within mucus containing extracellular DNA structures. At present, the evidence is stronger for fungal immobilization, increased mucus viscosity, and amplification of local inflammation than for direct fungicidal activity [77]. Therefore, in ABPA and related eosinophilic airway disorders, EETosis may help contain fungal material at epithelial surfaces, whereas persistent EET-rich mucus may worsen obstruction, epithelial irritation, and chronic eosinophilic inflammation. Current evidence distinguishes descriptive human observations from mechanistic in vitro findings. EET-containing mucus has been demonstrated in patients with ABPA, but these studies do not quantify associations between EET burden and lung function, exacerbation frequency, radiological mucus plugging, or treatment response [65,66]. By contrast, the proposed roles of CD11b, Syk, PI3K/Akt, calcium, and p38 MAPK were established using purified eosinophils from healthy donors, generally in only three to seven independent experiments and with pharmacological inhibitors [77,108]. These studies define plausible signaling pathways but do not establish their importance in vivo. Moreover, the evidence supports fungal entrapment more strongly than direct fungal killing, and the relative protective and pathological effects of EETs in ABPA remain unresolved.

Overall, the protective role of EETs is best understood as a context-dependent mechanism that limits pathogen spread, rather than as a uniform form of direct pathogen killing. By releasing extracellular DNA networks associated with eosinophil granule proteins, eosinophils may help immobilize bacteria, fungi, and parasite-derived material at sites where classical phagocytosis is inefficient. This response may be beneficial when it restricts pathogens at damaged mucosal or epithelial barriers, but it may become harmful when excessive or persistent EET formation increases mucus viscosity, epithelial injury, and chronic eosinophilic inflammation. These pathogen-related aspects of EET activity are summarized in Table 3.

## 6. Pathological Roles of Eosinophil Extracellular Traps

Eosinophil extracellular traps are increasingly recognized in eosinophil-associated disorders, including allergic diseases, gastrointestinal and skin inflammation, hypereosinophilic syndromes, infections, and selected neoplastic conditions [58,109,110]. Although these disorders differ in etiology and tissue distribution, they often share prominent eosinophil infiltration, extracellular deposition of eosinophil products, epithelial or stromal injury, and persistent local inflammation. In this context, EETs should be viewed as active extracellular structures that can amplify tissue pathology when their formation is excessive, prolonged, or inefficiently cleared [58,87]. Double-stranded DNA within EETs may be sensed by pattern recognition receptors on immune and resident tissue cells, promoting cytokine release and sterile inflammation [74,111]. At the same time, EET-associated granule proteins can damage epithelial cells, increase tissue permeability, and impair barrier repair [20]. Together, these effects may create a sustained inflammatory cycle in which barrier damage promotes further eosinophil recruitment and activation, while newly formed EETs maintain local tissue injury and immune activation [110]. Because extracellular DNA can contribute to the structure of bacterial biofilms, persistent DNA released through EETs may participate in inflammation associated with biofilm formation [112]. However, its precise role in human biofilms remains insufficiently defined.

The pathogenic effects of EETs may be particularly important in tissues with eosinophilic and type 2 inflammation, where epithelial activation, eosinophil survival signals, mucus abnormalities, and impaired clearance can sustain one another [87]. EETs may directly stimulate eosinophils by promoting morphological activation, degranulation, and reactive oxygen species production, thereby creating an autocrine amplification loop [85]. EETs may also activate epithelial cells. In airway and nasal epithelial cell cultures, EET exposure has been associated with epithelial detachment, increased permeability, IL-8 production, and eotaxin-3 expression. These epithelial changes may then promote further recruitment of inflammatory cells, including eosinophils [68,85]. EETs may contribute to mucus pathology by changing the physical properties of airway secretions. Extracellular DNA can increase secretion density and viscosity, while galectin-10 released in eosinophilic inflammation may crystallize as Charcot–Leyden crystals, further impairing mucus clearance and prolonging local inflammatory activity [54,68,113]. Thus, EETs appear to contribute to pathology through a combination of persistent extracellular DNA, granule protein toxicity, epithelial barrier injury, mucus thickening, biofilm support, and sustained eosinophilic type 2 inflammation. However, it remains difficult to determine which mechanism is dominant, because EETs often coexist with degranulation, cytolysis, cytokine release, microbial stimulation, and broader tissue damage.

### 6.1. EETosis in Eosinophilic Upper Airway and Otologic Mucosal Inflammation

Eosinophilic inflammation of the upper airway and middle ear mucosa is relevant to EETosis because these sites often combine barrier dysfunction, type 2 immune activation, microbial exposure, and impaired secretion clearance [64,114]. Under these conditions, EET formation may promote the persistence of extracellular DNA and granule proteins within mucosal secretions, thereby sustaining local inflammation [54,67]. This concept is particularly relevant to eosinophilic chronic rhinosinusitis with nasal polyps (CRSwNP) and otitis media with effusion (OME), where extracellular DNA and eosinophil products may contribute to the persistence of viscous secretions, refractory inflammation, and long-term disease persistence [67,114,115,116].

#### 6.1.1. EETs in Eosinophilic Chronic Rhinosinusitis and Nasal Polyps

Chronic rhinosinusitis (CRS) is a persistent inflammatory disorder of the nasal and paranasal sinus mucosa, commonly associated with nasal obstruction, rhinorrhea, facial pressure, and impaired olfaction [117]. It is traditionally classified according to the presence or absence of nasal polyps as CRS with nasal polyps (CRSwNP) or CRS without nasal polyps (CRSsNP) [114,118]. Patients may differ substantially in their underlying inflammatory profile [118]. Increasing attention has therefore focused on eosinophilic CRS, a disease form characterized by dominant type 2 inflammation, in which epithelial alarmins, activated eosinophils, and eosinophil products influence the local mucosal response [119]. In eosinophilic CRS, EETs may provide a mechanism through which activated eosinophils contribute to epithelial injury, microbial persistence, mucus abnormalities, and refractory polypoid airway inflammation [67].

CRSwNP is generally considered one of the more severe CRS phenotypes and is often associated with type 2 inflammation and eosinophil predominance [120]. In this eosinophilic mucosal environment, eosinophils are exposed to survival, recruitment, and activation signals, including IL-5, IL-4, IL-13, interleukin-33 (IL-33), TSLP, eotaxins, and related inflammatory mediators [120,121]. This inflammatory environment supports eosinophil persistence, degranulation, and extracellular trap formation through abundant cytokines and epithelial alarmins. Eosinophils in nasal polyps also show a more activated phenotype than circulating eosinophils, as indicated by increased expression of activation markers such as cluster of differentiation 69 (CD69) [122,123]. Once activated, these cells release MBP, EPX/EPO, ECP, EDN, extracellular DNA (eDNA), and galectin-10-containing material. These products can damage the epithelium, alter mucus properties, promote EET formation, and interact with microbial and epithelial signals in the inflamed mucosa [123,124]. EETs have been detected in nasal polyp tissue, inflamed sinonasal mucosa, and nasal secretions, supporting their relevance in eosinophilic CRS [67,114]. Their presence is not limited to polypoid tissue, suggesting that EETosis is associated more with eosinophilic type 2 inflammation than with nasal polyps alone [67]. In line with this, EET-releasing eosinophils are enriched in the subepithelial region of sinonasal tissue, where epithelial injury signals, microbial products, alarmins, and chemotactic mediators may support local EET formation [64,67]. The association between EET formation and higher tissue IL-5 levels further supports the role of type 2 cytokine signaling in local eosinophil activation and trap release [114].

Findings from eosinophilic nasal polyps also suggest that metabolic features of the inflammatory microenvironment may promote EETosis [82]. Lipidomic profiling has shown selective accumulation of long-chain fatty acids in eosinophilic nasal polyps, while transcriptomic data provide complementary evidence of upregulated genes related to long-chain unsaturated fatty acid metabolism [82,125]. In this lipid-rich tissue environment, long-chain fatty acids, particularly arachidonic acid, may activate eosinophils and induce filamentous extracellular DNA release [82,125]. Arachidonic acid may also promote galectin-10 crystallization and Charcot–Leyden crystal formation, thereby connecting altered lipid metabolism with EET formation and mucus pathology in eosinophilic inflammation [82,125,126]. At the signaling level, this lipid-related form of EETosis appears to differ from classical trap formation driven by the oxidative burst. Arachidonic acid can promote extracellular DNA release through inositol-requiring enzyme 1α (IRE1α)/spliced X-box binding protein 1 (XBP1s) signaling and PAD4 activation [82]. These findings suggest that EETosis in nasal polyps is not driven only by cytokines or microbes, but may also be reinforced by local lipid mediators that sustain eosinophil activation. The lipidomic and transcriptomic findings provide human tissue support for altered fatty-acid metabolism, whereas the proposed IRE1α/XBP1s/PAD4 pathway and resistance of EET formation to glucocorticoids were demonstrated mainly in isolated peripheral-blood eosinophils [82]. Several functional experiments included only four independent samples. Therefore, these results define a plausible mechanism but do not demonstrate that arachidonic acid-driven EETosis causes corticosteroid resistance or predicts treatment failure in patients with CRSwNP.

The relationship between EETs, galectin-10, Charcot–Leyden crystals, and mucus properties is important for understanding the clinical features of eosinophilic CRS. Mucus from patients with eosinophilic CRS contains increased levels of eosinophil-associated proteins, particularly galectin-10 and EDN, and shows higher viscosity, dry weight, hydrophobicity, and radiological density than mucus from non-eosinophilic disease [113]. Extracellular DNA released during EETosis can make mucus thicker by retaining eosinophil granule proteins, cellular debris, bacteria, and other inflammatory components. As a result, mucus clearance becomes more difficult and secretion stasis may develop [54,67]. Galectin-10 can crystallize into Charcot–Leyden crystals, which may further impair secretion clearance and contribute to persistent local inflammation [68]. These findings suggest that mucus obstruction in eosinophilic CRS reflects not only edema or glandular secretion, but also the structural and biochemical consequences of eosinophil activation, extracellular trap release, galectin-10 crystallization, and persistence of eosinophil-derived material within the sinonasal lumen. Barrier dysfunction and bacterial colonization may further amplify this process. In nasal polyps, impaired epithelial tight junctions, altered macrophage responses, reduced β-defensin activity, and weakened innate immune sensing may compromise mucosal defense. This may allow bacteria, particularly *Staphylococcus aureus*, to come into closer contact with subepithelial eosinophils at sites of epithelial damage [64]. This contact may promote additional local EET formation, while the resulting extracellular DNA, bacterial structures, cytotoxic granule proteins, and inflammatory mediators become retained within thick mucus, thereby worsening epithelial injury and perpetuating local inflammation [54].

Human studies support an association between EET burden and CRS severity, but they do not establish causality. In a study of 43 patients, the number of EET-releasing eosinophils correlated with tissue eosinophilia (*r* = 0.83), the Lund–Mackay CT score (*r* = 0.51), blood eosinophilia (*r* = 0.80), and impaired olfactory function (*r* = −0.65) [67]. In another study of 117 patients with CRSwNP, median EET counts were higher in refractory than in non-refractory disease (19.50 versus 8.67 per high-power field, *p* = 0.029), whereas the proportion of EET-forming eosinophils did not differ significantly (*p* = 0.210) [114]. Refractoriness increased from 30.77% in the low-ETosis group to 83.33% in the high-ETosis group [114]. However, EET burden is closely related to tissue eosinophil abundance, and its independent prognostic value remains uncertain. These findings should also be interpreted cautiously because the studies differed in patient selection, EET quantification, follow-up, and definitions of disease severity and refractoriness.

#### 6.1.2. EETs in Otitis Media with Effusion

Otitis media with effusion (OME) is a chronic inflammatory condition of the middle ear characterized by fluid accumulation behind an intact tympanic membrane, often in the setting of Eustachian tube dysfunction [127]. Although OME is usually discussed in relation to ventilation failure, bacterial persistence, and mucosal inflammation, extracellular trap formation may provide an additional mechanism contributing to the viscosity and persistence of middle ear effusion [128]. Eosinophils may contribute to OME through type 2 cytokine activity and the release of eosinophil products, which can promote chronic mucosal inflammation and exudate formation [129]. In middle-ear effusion from patients with OME, increased levels of double-stranded DNA (dsDNA) and citrullinated histone H3–DNA complexes indicate extracellular trap-associated inflammation, while detection of ECP–DNA complexes points more specifically to an eosinophil-related trap component [130]. This distinction is important because dsDNA and citrullinated histone H3–DNA complexes may reflect mixed extracellular trap activity, whereas ECP–DNA complexes indicate that extracellular DNA is associated with eosinophil cationic protein [130]. The detection of additional granule proteins from eosinophils, including MBP, further supports eosinophil involvement in the extracellular inflammatory material present in middle ear effusions [129].

The inflammatory conditions in OME may favor the persistence of extracellular traps and continued mucosal activation [130]. Elevated IL-5 and IL-13 suggest type 2 inflammation with eosinophil involvement, whereas increased IL-6 and IL-1β indicate broader inflammatory activity within the middle ear cavity [130,131]. In this setting, EETs may contribute to disease persistence by forming extracellular DNA–protein networks that retain inflammatory mediators, increase effusion viscosity, activate danger-sensing pathways, and prolong mucosal irritation [54,130]. These DNA-rich networks may also activate danger-sensing pathways, particularly Toll-like receptor 9 (TLR9) and nuclear factor kappa B (NF-κB) signaling, because extracellular DNA can function as an immunologically active signal capable of sustaining cytokine production rather than merely as a structural component of traps [45,130]. Experimental results showing that dsDNA binding can reduce trap-related inflammatory signaling support the possibility that extracellular DNA contributes to mucus-like exudation and middle ear inflammation [128,130]. However, clinical translation still needs to be validated, and it remains unclear how much of the inflammatory burden in human OME is driven specifically by EETs rather than by NETs, bacterial products, epithelial injury, or mixed leukocyte activation.

Evidence from eosinophilic otitis media should be distinguished from evidence obtained in conventional OME. The initial human study detected histone-positive extracellular DNA structures in all eight examined patients with eosinophilic otitis media, but all patients also had asthma and CRSwNP, and the study lacked a control group [5]. More recent evidence in OME includes analysis of 31 human middle-ear effusions, whereas the proposed effects of extracellular DNA targeting on TLR9/NF-κB signaling, mucous exudation, inflammation, and hearing were demonstrated mainly in an ovalbumin-induced rat model [22]. Moreover, dsDNA and citrullinated histone–DNA complexes may originate from multiple leukocyte populations, limiting their specificity for EETs. Thus, current findings support the presence of mixed extracellular-trap activity but do not establish that EET burden independently determines effusion viscosity, hearing loss, disease persistence, or treatment response in human OME.

### 6.2. EETosis in Airway and Pulmonary Eosinophilic Inflammation

Airway and pulmonary diseases provide a particularly relevant setting for EETosis because eosinophils accumulate at mucosal and parenchymal sites exposed to allergens, pollutants, microbial products, epithelial injury, and persistent inflammatory signaling [19]. Under these conditions, EETs may contribute to local host defense and immune activation, but sustained trap formation can increase the extracellular DNA burden, impair mucus clearance, damage the epithelium, and reinforce granulocytic inflammation [87]. This mechanism is especially relevant in asthma, selected eosinophilic phenotypes of chronic obstructive pulmonary disease, and chronic eosinophilic pneumonia, where EETosis may link eosinophil activation to airway obstruction, tissue injury, and disease persistence [85,87,132,133].

#### 6.2.1. EETs in Asthma

Asthma is a heterogeneous inflammatory airway disease characterized by bronchial hyperresponsiveness, variable airflow limitation, mucus hypersecretion, and recurrent respiratory symptoms [134]. EETosis is particularly relevant in severe eosinophilic asthma, where persistent airway and systemic eosinophilia, enhanced type 2 inflammation, mucus dysfunction, and structural airway remodeling often coexist [85,87]. In this setting, activated eosinophils in the airway mucosa and lumen can release cytotoxic granule proteins, lipid mediators, cytokines, reactive oxygen species, and extracellular DNA structures, thereby linking eosinophil activation to epithelial injury, mucus abnormalities, and amplification of airway inflammation [20,85,87].

The type 2 inflammatory microenvironment in eosinophilic asthma provides several signals that favor EET formation [87,135]. IL-5 supports eosinophil survival, activation, and responsiveness to secondary stimuli, whereas IL-13 promotes mucus production, epithelial remodeling, and airway hyperresponsiveness [136,137]. At the same time, epithelial barrier stress leads to the release of alarmins such as IL-33 and TSLP, which further amplify eosinophil activation and innate type 2 immunity. These type 2 signals may be further strengthened by additional inflammatory stimuli, including TNF-α, IL-1β, IFN-γ, microbial products, endotoxins, immune complexes, and oxidative stress [76]. Taken together, the airway environment, with abundant cytokines, epithelial alarmins, and danger signals, may prime eosinophils and provide the secondary stimulation required for extracellular trap formation.

Evidence for EETs in asthma comes from airway samples, experimental allergic airway models, and studies of severe eosinophilic asthma. EETs have been detected in the lungs and airway samples of patients with asthma, while ovalbumin-induced allergic airway inflammation has been associated with increased EET release in bronchoalveolar lavage fluid, greater cellular infiltration, airway inflammation, and mucus production [76,138,139]. Immunofluorescent staining demonstrated colocalization of extracellular DNA with EPX, supporting the eosinophilic origin of these traps and indicating that DNA fibers were associated with granule proteins involved in tissue injury [138]. In severe asthma, peripheral blood eosinophils show a greater capacity to form EETs than eosinophils from patients with non-severe asthma or from healthy individuals, particularly after stimulation with IL-5 and lipopolysaccharide [85]. This increased capacity for EET formation has been associated with lower forced expiratory volume in 1 s (FEV1) and higher serum EDN levels, suggesting that EETosis may be related to airflow limitation and systemic eosinophil activation [85,87]. In a cohort of 40 patients, including 20 patients with severe eosinophilic asthma and 20 with non-severe asthma, the percentage of IL-5/LPS-induced EET-forming peripheral-blood eosinophils was significantly higher in severe eosinophilic asthma (*p* = 0.009). Across the combined asthma cohort, the percentage of EET-forming eosinophils correlated inversely with baseline FEV1% predicted (r = −0.350, *p* = 0.027) and directly with serum EDN concentration (r = 0.437, *p* = 0.018) [85]. In an independent study including BALF samples from 46 patients with asthma, the proportion of BALF area occupied by EETs was inversely associated with FEV1% predicted (R^2^ = 0.321, *p* < 0.0001) and positively associated with asthma classification level (R^2^ = 0.345, *p* < 0.0001). In a subset of 20 patients, BALF EET area was positively associated with BALF concentrations of IL-4 (R^2^ = 0.400, *p* = 0.0028), IL-5 (R^2^ = 0.509, *p* = 0.0004), and IL-13 (R^2^ = 0.305, *p* = 0.0116) [140]. By contrast, in the COBRA cohort of 217 patients with asthma, circulating DNA–eosinophil major basic protein complexes were not associated with asthma severity. EETs were mostly undetectable in the available BALF samples, and circulating EET levels showed only a mild association with asthma control, for which an effect estimate was not reported [141]. None of these human studies reported an EET-specific association with annual exacerbation frequency or a longitudinal change in EET burden following corticosteroid or biological treatment.

From a signaling perspective, ROS-dependent signaling appears to play an important role in EET formation [79]. In ovalbumin-induced asthma models, treatment with N-acetylcysteine or diphenyleneiodonium reduces EET formation in bronchoalveolar lavage eosinophils, indicating that oxidative pathways, particularly NADPH oxidase-derived oxidants, contribute to trap release [79,142]. However, ROS involvement does not by itself define the cellular source of the extracellular DNA. In experimental asthma models, EETs from bronchoalveolar lavage eosinophils do not consistently colocalize with histone H2B, suggesting that at least part of the released DNA may be mitochondrial rather than nuclear [76,79]. This interpretation is further supported by the lack of annexin V and propidium iodide positivity and by preserved cell viability [79]. Together, these findings suggest vital EETosis, with mitochondrial DNA release occurring without immediate eosinophil death. These findings do not exclude cytolytic nuclear EETosis in patients with severe asthma, but they suggest that asthma may involve multiple forms of eosinophil trap formation, depending on the stimulus, disease stage, and local airway environment.

The pathogenic consequences of EETs in asthma are closely related to their effects on mucus and epithelium. Extracellular DNA fibers can increase mucus viscosity, while eosinophil granule proteins such as EPX/EPO, MBP, ECP, and EDN disrupt barrier integrity and impair ciliary function, thereby further reducing mucociliary clearance [20,54]. In severe asthma, this combination of thicker secretions, epithelial injury, and impaired clearance may favor mucus plugging, airway obstruction, and reduced airflow [143]. EETs may also directly affect airway epithelial cells because extracellular DNA fibers carrying eosinophil granule proteins can interact with the epithelial surface, induce cellular stress and injury, disrupt epithelial adhesion and barrier integrity, and thereby promote epithelial detachment, increased permeability, and IL-6 and IL-8 release [85,87]. Epithelial activation may then reinforce type 2 inflammation through IL-33 and TSLP, which activate group 2 innate lymphoid cells (ILC2s) and promote IL-5 and IL-13 production [69,87]. This creates a feed-forward loop in which eosinophils release EETs, EETs activate epithelial inflammatory programs, epithelial alarmins stimulate ILC2s, and ILC2-derived cytokines maintain eosinophil-dominant inflammation.

EETs may further amplify asthma inflammation through autocrine eosinophil activation. Material released within extracellular traps may in turn activate eosinophils, promoting cellular activation, degranulation, and ROS production. This suggests a feedback loop in which eosinophils can both release and respond to extracellular traps [85]. A related mechanism has been proposed involving cytokeratin 18, an epithelial autoantigen increased in severe asthma and associated with eosinophil counts and impaired lung function [144]. In this proposed axis, epithelial injury may increase CK18 exposure or release, whereas the presence of CK18-directed IgG provides a potential link between epithelial autoantigen availability and eosinophil activation. CK18-specific IgG can promote eosinophil degranulation and EET release, while EET-derived material may further stimulate airway epithelial cells to release CK18, sustaining an epithelial injury–eosinophil activation loop [144].

EETs have also been proposed as biomarkers of severe asthma and acute inflammatory activity [141]. Several studies suggest that circulating EET markers or ex vivo inducible EET formation may indicate a more activated eosinophilic phenotype, with associations with impaired lung function and increased EDN levels [85,87,141]. However, this interpretation has not been fully established. Some studies have not found a clear association between serum EETs and asthma severity, and EET detection in bronchoalveolar lavage fluid may vary between cohorts [87,141]. These discrepancies may reflect differences in sample type, timing of collection, disease phenotype, treatment exposure, and methods used to define EETs. They may also be influenced by the presence of neutrophil extracellular traps in mixed granulocytic asthma [87]. At present, EETs should be considered promising but still unvalidated biomarkers. Their strongest relevance remains mechanistic, as they may connect eosinophil activation and oxidative stress with epithelial injury, mucus plugging, and reinforcement of type 2 inflammation in severe asthma.

Although asthma currently provides the strongest human evidence for the clinical relevance of EETs, the available findings remain inconsistent. The reported associations with FEV_1_, asthma severity, and type 2 cytokines were derived from cross-sectional cohorts using different samples and EET measurements [85,140,141]. Ex vivo EET formation by circulating eosinophils should also be distinguished from EET burden measured directly in the airways. Moreover, the proposed neuroimmune, epithelial, and ILC2-related mechanisms were established predominantly in cell-culture experiments and animal models [69,140]. No study has yet demonstrated that EET burden independently predicts exacerbations, longitudinal lung-function decline, or response to corticosteroids or biological therapies. Therefore, EETs remain promising but unvalidated biomarkers and therapeutic targets in asthma.

#### 6.2.2. EETs in Chronic Obstructive Pulmonary Disease

Chronic obstructive pulmonary disease (COPD) is a heterogeneous inflammatory airway disorder characterized by persistent airflow limitation, recurrent exacerbations, epithelial injury, and mucus dysfunction [145]. Although cigarette smoke and other inhaled irritants are major drivers of COPD, disease susceptibility and progression cannot be explained by exposure alone, suggesting that distinct inflammatory pathways contribute to individual disease phenotypes [132,146]. Eosinophils are not dominant in all patients with COPD, but blood and sputum eosinophilia have been described in a clinically relevant subset, particularly among patients with frequent exacerbations or steroid-responsive inflammatory features [146,147]. Available evidence suggests that exacerbation-prone eosinophilic COPD may involve numerous eosinophils undergoing cytolytic EETosis, together with abundant extracellular cellular debris and chromatin-rich material in the airway lumen [58,132]. In this eosinophil-rich phenotype, cytolytic EETosis may contribute to airway obstruction by depositing extracellular DNA, decondensed chromatin, and granule-derived proteins directly into mucus-rich airway secretions. The DNA-rich material can increase secretion viscosity and retain cellular debris, bacterial products, and inflammatory mediators, thereby impairing mucociliary clearance and promoting luminal obstruction. At the same time, eosinophil-derived granule proteins and extracellular DNA may injure the epithelium and activate danger-sensing inflammatory pathways, sustaining persistent airway inflammation [132]. Another possible mechanism involves interaction between EETosis and NETosis in mixed granulocytic COPD inflammation [132]. During severe exacerbations, eosinophil cytolysis may release membrane debris, extracellular DNA, chromatin-rich material, and granule-derived proteins into the airway lumen. This eosinophil-derived material may be taken up by neutrophils or act as a local danger signal, promoting neutrophil activation at the same time that early NETosis-like changes are observed [132,148]. Such crosstalk could couple eosinophil cytolysis with neutrophil extracellular trap formation (NETosis): EET-related material would amplify neutrophil activation, while NETosis would further increase extracellular DNA, protease-rich material, and inflammatory mediators within airway secretions [132,148]. The result may be a higher total extracellular DNA burden, greater mucus viscosity, and more persistent airway inflammation. Evidence for EETosis in COPD remains considerably weaker than that in asthma. It is based mainly on morphological analysis of induced sputum from a single cross-sectional study, which identified EETosis predominantly in eosinophilic or mixed inflammatory phenotypes [132]. Although the inclusion of several COPD severity groups was a strength, EET burden was not evaluated as a standardized quantitative biomarker and was not related prospectively to FEV_1_ decline, exacerbation frequency, mucus plugging, or treatment response. The proposed interaction between EETosis and NETosis is therefore based primarily on spatial and morphological observations rather than direct functional evidence. Further clinical validation is required before EETosis can be considered an independent mechanism or biomarker of COPD progression.

#### 6.2.3. EETs in Chronic Eosinophilic Pneumonia

Chronic eosinophilic pneumonia is an eosinophil-dominant inflammatory disease of the lower respiratory tract, characterized by eosinophilic infiltration of the lung parenchyma and airspaces [149]. This makes it a relevant setting for examining whether extracellular trap formation also occurs in eosinophil-rich pulmonary inflammation. Direct evidence remains limited, but one case report described EET-like structures in bronchoalveolar lavage fluid (BALF) from a patient with chronic eosinophilic pneumonia [133]. The lavage fluid contained abundant eosinophils, and immunostaining for major basic protein showed eosinophil-rich aggregates, supporting local eosinophil activation and granule protein deposition [133]. In parallel, citrullinated histone H3 staining revealed network-like extracellular structures consistent with extracellular trap formation [133]. Taken together, eosinophil-rich BALF, MBP-positive aggregates, and CitH3-positive extracellular networks suggest that EETosis may occur within the lower airways in chronic eosinophilic pneumonia. Current evidence in chronic eosinophilic pneumonia is limited to a single case report [133]. Although MBP-positive eosinophil aggregates and citrullinated histone H3-positive extracellular structures support possible local EET formation, citrullinated histones are not eosinophil-specific and the DNA origin was not established. The absence of controls, quantitative assessment, and longitudinal EET measurements prevents conclusions regarding disease severity, treatment response, or causality. EETosis in chronic eosinophilic pneumonia should therefore be presented as a preliminary observation requiring confirmation in larger patient cohorts.

### 6.3. EETosis in Eosinophil-Rich Systemic Vasculitic and Hypereosinophilic Disorders

Eosinophil-rich systemic diseases provide an important framework for understanding EETosis beyond mucosal inflammation [72]. In these conditions, persistent eosinophil activation may involve blood vessels, parenchymal organs, thromboinflammatory lesions, and fibrotic remodeling [72,150]. In this context, IL-5 may prolong eosinophil survival and enhance their activation, thereby favoring cytolytic EETosis and extracellular accumulation of DNA, chromatin material, and eosinophil granule protein [72]. These structures may then contribute to endothelial injury, platelet recruitment, fibroblast activation, and local tissue damage, thereby linking eosinophil activation to vascular inflammation and organ dysfunction [151]. This concept is particularly relevant to eosinophilic granulomatosis with polyangiitis (EGPA), hypereosinophilic syndrome (HES) with cardiac involvement, and eosinophil-dominant forms of antineutrophil cytoplasmic antibody (ANCA)-associated vasculitis (AAV). In these diseases, intense eosinophil activation and EETosis may contribute to vascular inflammation, thrombotic complications, and fibroinflammatory remodeling [72,152].

#### 6.3.1. EETs in Eosinophilic Granulomatosis with Polyangiitis

Eosinophilic granulomatosis with polyangiitis, previously known as Churg–Strauss syndrome, is a rare systemic vasculitis within the spectrum of anti-neutrophil cytoplasmic antibody-associated vasculitides [150]. It is characterized by eosinophil-rich granulomatous inflammation and necrotizing vasculitis of small- to medium-sized vessels [150]. Its clinical presentation is heterogeneous and may include asthma, pulmonary infiltrates, ear, nose, and throat involvement, peripheral neuropathy, skin, gastrointestinal and renal manifestations, and potentially severe cardiac disease [153]. Eosinophils have a central role in EGPA pathology, since tissue eosinophilia represents a defining histological feature of the disease. The clinical efficacy of eosinophil-targeted therapy, particularly anti-IL-5 treatment, further supports the functional importance of IL-5-driven eosinophil activation in selected patients [154,155,156]. In this context, EETosis has emerged as a possible mechanism linking eosinophil activation to cytolytic tissue injury, extracellular granule protein deposition, and systemic inflammation. Experimental studies using eosinophils isolated from patients with EGPA have shown that these cells are prone to undergo EETosis after stimulation with PMA or autoimmune antibodies [72,157]. At the molecular level, this response involves ROS generation and NOX activation, followed by PAD4-dependent histone citrullination and cytolytic release of CitH3-positive extracellular traps [72,86]. Eosinophils undergoing EETosis may also release cell-free granules, allowing extracellular chromatin and eosinophil granule mediators to accumulate together in affected tissues [72]. Tissue analyses further support this interpretation by showing eosinophils with features compatible with cytolytic activation, extracellular trap formation, and deposition of eosinophil-derived material in involved organs [72,151]. Pathophysiologically, this is important because EETosis may provide a mechanism by which activated eosinophils deposit DNA, chromatin-rich material, and cytotoxic granule proteins directly within vascular and perivascular lesions. These extracellular structures may amplify endothelial injury, sustain innate immune activation, promote thromboinflammatory reactions, and contribute to granulomatous tissue damage and organ dysfunction. Because EGPA is associated with intense eosinophil activation and cytolysis, galectin-10 is also relevant to this process [158]. As one of the most abundant eosinophil proteins, galectin-10 may be released during EETosis or other forms of eosinophil cytolytic activation and can crystallize into Charcot–Leyden crystals [158]. In this way, galectin-10 may reflect the extracellular accumulation of eosinophil-derived material and may also contribute to the persistence of eosinophil-associated inflammatory deposits within affected tissues. Increased serum galectin-10 in active EGPA, compared with more stable asthma, suggests that widespread eosinophil cytolysis and possibly systemic EETosis may contribute to circulating galectin-10 levels [72]. However, galectin-10 should be interpreted cautiously as a biomarker because it reflects eosinophil burden and activation more broadly and is not specific for EETosis alone. Overall, available evidence supports a model in which IL-5-dependent eosinophil activation, oxidative signaling, PAD4-mediated chromatin modification, cytolytic EET release, cell-free granule deposition, and galectin-10/Charcot–Leyden crystal biology converge to amplify eosinophil-driven vascular, granulomatous, and systemic tissue injury in EGPA. Although EGPA provides direct human tissue evidence of EETosis, the available clinical studies remain small and largely observational. In one study, circulating nuclear and mitochondrial cell-free DNA correlated with disease activity and decreased after immunosuppressive treatment, but only 10 patients with EGPA were examined [151]. Moreover, circulating cell-free DNA cannot currently be attributed specifically to eosinophils or EETosis. Associations with D-dimer and the presence of EET-like structures in thrombi therefore support a possible link with immunothrombosis but do not demonstrate that EETs independently cause thrombosis, organ damage, relapse, or treatment failure. Galectin-10 should likewise be regarded as a marker of eosinophil activation and cytolysis rather than a specific biomarker of EETosis.

#### 6.3.2. EETs in Hypereosinophilic Syndrome: Myocardial Fibrosis and Fibroblast Activation

Hypereosinophilic syndrome with cardiac involvement represents an important clinical example of eosinophil-driven tissue injury, in which persistent eosinophil activation may progress from myocardial inflammation to endomyocardial damage, fibrosis, and functional impairment [159]. In this setting, markers of eosinophil activation may be more informative than the absolute eosinophil count, especially after tissue remodeling has begun [160]. Recent imaging-based data suggest a possible link between circulating EET levels and fibroblast activation in HES-related cardiac disease [160]. Al^18^F-FAPI-04 PET/CT enables noninvasive assessment of fibroblast activation protein expression and may therefore detect active myocardial remodeling before advanced fibrosis becomes evident on conventional cardiac evaluation [160,161]. Myocardial fibroblast activation protein inhibitor (FAPI) uptake appears heterogeneous: extensive uptake may reflect diffuse fibroblast activation and broader remodeling, whereas patchy uptake may indicate more focal or less advanced myocardial involvement [160]. Importantly, myocardial FAPI uptake correlates more strongly with circulating EET levels than with the absolute eosinophil count, suggesting that EETs may better reflect tissue-directed pathogenic eosinophil activation associated with cardiac remodeling [160]. Within injured myocardial tissue, cytolytic EETosis may promote extracellular deposition of DNA, chromatin material, and eosinophil granule proteins. This material may sustain local inflammation and cellular stress, while also promoting profibrotic signaling, fibroblast activation, and subsequent fibrosis [34,40]. However, this interpretation remains associative. It is still unclear whether EETs directly activate cardiac fibroblasts, mainly mark more intense eosinophil-mediated myocardial injury, or both. Therefore, in hypereosinophilic cardiac disease, EETs should be viewed as potential links between eosinophil cytolysis and myocardial remodeling, while their causal role in fibroblast activation and fibrotic progression remains to be confirmed in larger clinical and experimental studies. The relationship between EETs and cardiac remodeling in HES is supported by a single-center, cross-sectional study of only 20 patients [160]. Although myocardial FAPI uptake was associated with circulating EET levels but not with the absolute eosinophil count, the study did not include longitudinal outcome assessment or direct experimental evidence that EETs activate cardiac fibroblasts. The findings therefore identify an association between EET burden and fibroblast activation but do not establish causality or demonstrate that EET levels predict progression, heart failure, or treatment response.

#### 6.3.3. EETs in Sepsis

Sepsis provides a systemic inflammatory setting in which EETosis may influence both bacterial control and inflammatory tissue injury [58,59]. Experimental studies using IL-5 transgenic mice subjected to cecal ligation and puncture have shown that enhanced eosinophil expansion and intestinal tissue infiltration are associated with detectable EET formation and improved protection against sepsis [58,59,162]. These findings suggest that, when eosinophils are sufficiently expanded and activated, they may contribute to antibacterial defense by releasing extracellular DNA traps at infected tissue sites [59]. This interpretation is supported by in vitro observations showing that activated eosinophils can release EETs in response to opsonized *Escherichia coli* and mediate antibacterial activity through a largely phagocytosis-independent mechanism [58]. However, the same extracellular material may become harmful during systemic inflammation if trap formation is excessive or inefficiently cleared. Persistent extracellular DNA, histones, and eosinophil granule proteins may activate innate immune sensing pathways, amplify cytokine release, damage endothelial or epithelial barriers, and contribute to immune dysregulation [163,164]. Therefore, EETosis in sepsis should be interpreted cautiously: available data support a potential role in local bacterial containment and improved survival under selected experimental conditions, but uncontrolled EET accumulation may also increase tissue injury and the systemic inflammatory burden. Evidence for EET involvement in sepsis remains entirely preclinical and partly conflicting. The beneficial findings derive from genetically modified mice and isolated eosinophils, whereas the proposed harmful effects of extracellular DNA and histones are extrapolated mainly from NET and circulating-histone studies rather than demonstrated specifically for EETs. No human study has quantified EET burden in sepsis or related it to bacterial clearance, organ failure, mortality, or treatment response. EETosis should therefore be presented as a possible, but unconfirmed, contributor to host defense and inflammatory injury in sepsis.

### 6.4. EETs in Inflammatory Skin Diseases

The skin provides a relevant tissue context for eosinophil extracellular trap formation because it is an immunologically active barrier and a frequent site of eosinophil-rich inflammation [165]. In inflammatory dermatoses, eosinophils may participate in host defense, allergic inflammation, tissue injury, and barrier dysfunction [165]. EETs have been detected in several types of skin disease. These include infectious and parasite-associated disorders, such as ectoparasitosis and cutaneous larva migrans; allergic and reactive dermatoses, including atopy patch test reactions, allergic contact dermatitis, and drug hypersensitivity reactions; autoimmune bullous diseases, such as bullous pemphigoid, pemphigus foliaceus, and dermatitis herpetiformis; and systemic eosinophilic disorders with cutaneous involvement [58,109]. This distribution suggests that cutaneous EETosis is not restricted to antimicrobial defense, but may also occur during sterile immune activation, when eosinophils accumulate in inflamed skin and are exposed to local activating signals [109]. Within this environment, eotaxins support eosinophil recruitment, tissue retention, and local accumulation, while IL-5 and IFN-γ may prime eosinophils for stronger effector responses and increase their readiness for extracellular trap formation [71]. Once activated, eosinophils may release extracellular DNA structures associated with granule-derived proteins, thereby contributing to epidermal barrier injury, local cytokine amplification, and persistent inflammation [109]. Available observations also suggest that EETs are more closely associated with acute or exacerbated lesions than with stable chronic disease. EETs have been detected in acute infectious dermatoses, allergic contact dermatitis, drug reactions, and positive atopy patch test reactions, but appear less consistently in some chronic lesions. This suggests that EETosis may reflect an early or more intense phase of eosinophil activation, rather than a constant feature of eosinophilic skin inflammation [109,166]. Therefore, in inflammatory skin disease, EETs may contribute to barrier defense against exogenous triggers, but persistent or excessive trap formation may also amplify local tissue injury, barrier dysfunction, and inflammatory activity.

#### 6.4.1. EETs in Wells Syndrome

Wells syndrome provides a distinctive cutaneous setting in which eosinophil-rich tissue injury may be structurally linked to extracellular trap formation [167]. Its histological hallmark is the presence of flame figures, dermal lesions traditionally interpreted as the result of eosinophil disruption, extracellular deposition of granule material, and accumulation of nuclear fragments [167]. Recent observations suggest that flame figures may contain organized extracellular DNA networks, rather than only nonspecific eosinophil debris [167]. Extracellular DNA structures that stain with propidium iodide have been detected within flame figures, supporting the presence of material consistent with EETs [167,168]. The nuclear origin of this DNA is further supported by colocalization with histone H2A and by proteomic findings showing abundant histone H2 within these lesions [169,170]. The presence of histones within the extracellular DNA material is important because it supports a nuclear origin of the DNA and makes cytolytic nuclear EETosis the more likely mechanism [34]. In Wells syndrome, cytolytically activated eosinophils may release nuclear chromatin together with eosinophil granule proteins. This material may contribute to flame figure formation and persist in the dermis as extracellular inflammatory deposits containing DNA, histones, granule material, and cellular debris [171]. However, this interpretation should remain cautious because flame figures are complex histological structures, and not all eosinophil-derived extracellular material within them necessarily reflects organized EETosis. Evidence linking EETosis to Wells syndrome remains based predominantly on small, descriptive human tissue studies. In the largest survey of eosinophilic skin diseases, EET-releasing cells generally represented less than 10% of tissue eosinophils, although proportions of up to 30% were observed in Wells syndrome [109]. A separate proteomic analysis identified extracellular DNA, histone H2A, and eosinophil proteins within flame figures, but included only two biopsies, only one of which was obtained from a patient with Wells syndrome [167]. These findings support compositional similarities between flame figures and EETs but do not demonstrate that all flame figures originate through EETosis. No study has quantitatively related cutaneous EET burden to lesion severity, recurrence, healing, or treatment response. Additional studies using eosinophil-specific trap markers, spatial colocalization of extracellular DNA with granule proteins, and ultrastructural confirmation are needed to distinguish true EET formation from nonspecific eosinophil breakdown.

#### 6.4.2. EETs in Bullous Pemphigoid

Bullous pemphigoid is the most common autoimmune subepidermal blistering disease and predominantly affects elderly individuals, in whom it may cause considerable morbidity and impaired quality of life [172]. The disease is classically driven by IgG autoantibodies against the hemidesmosomal proteins BP180 and BP230, which destabilize the dermal–epidermal junction and trigger inflammatory events leading to subepidermal blister formation [172,173]. Although autoantibody binding is the initiating immunological event, eosinophils are prominent effector cells within bullous pemphigoid (BP) lesions and blister fluid, indicating that tissue injury is not mediated by antibodies alone [174]. The blister microenvironment contains eosinophil-active mediators, including IL-5, eotaxin, and related cytokines and chemokines that support eosinophil recruitment, survival, priming, and local activation [175]. These eosinophil-directed signals occur within a broader pro-inflammatory cytokine milieu, as increased IL-1β, IL-6, IL-8, and TNF-α levels correlate with disease activity and may further amplify leukocyte recruitment, keratinocyte activation, and tissue injury at the dermal–epidermal junction [176]. In this setting, EETosis may represent an additional mechanism through which activated eosinophils sustain blister inflammation. EETs have been detected in skin biopsies from patients with bullous pemphigoid, supporting the presence of extracellular trap formation in autoimmune blistering skin disease [109,177]. Pathophysiologically, extracellular DNA networks may retain eosinophil granule proteins at the dermal–epidermal interface, amplify local cytokine signaling, and prolong inflammatory injury within the blister microenvironment [178]. A possible amplification pathway may involve blister fluid-derived exosomes and keratinocyte–eosinophil communication [179]. Exosomes from BP blister fluid can stimulate keratinocytes to release TNF-α, which may then promote eosinophil secretory activation, including eosinophil sombrero vesicle release, granule protein mobilization, and possibly EET-related activity [179]. Human tissue observations support the presence of EET-associated DNA and eosinophil proteins in bullous pemphigoid lesions, but their independent contribution to blister formation remains uncertain. Experimental models have shown that IL-5-activated eosinophils can promote dermal–epidermal separation in the presence of BP autoantibodies, but these studies did not establish that EET formation is required for this effect [178]. Similarly, blister fluid-derived exosomes activate keratinocytes and promote inflammatory signaling, but a direct effect on EETosis has not been demonstrated [179]. No available clinical study has quantitatively related EET burden to disease-activity scores, relapse, blister number, or treatment response. EETs should therefore be considered possible amplifiers of eosinophilic inflammation rather than established determinants of bullous pemphigoid severity. Thus, EETosis in BP is likely embedded in a broader interaction between autoantibodies, keratinocytes, cytokines, eosinophil vesicular transport, granule protein release, and extracellular DNA formation.

### 6.5. EETs in Allergic Ocular Surface Diseases

Allergic ocular surface diseases, particularly vernal keratoconjunctivitis (VKC) and atopic keratoconjunctivitis (AKC), are eosinophil-rich inflammatory disorders in which persistent conjunctival and corneal inflammation can lead to tissue damage and visual impairment [180]. In these conditions, eotaxins, including CCL11, CCL24/eotaxin-2, and CCL26/eotaxin-3, promote eosinophil recruitment into conjunctival tissue, while IL-5 supports eosinophil survival, activation, and readiness for degranulation and possible EET formation [181,182,183]. The classical pathogenic role of eosinophils in VKC and AKC is linked to the release of cationic granule proteins, which can damage corneal epithelial and stromal cells and contribute to epithelial erosions, shield ulcers, and persistent ocular surface irritation [184]. Additional eosinophil-derived mediators, including transforming growth factor-β (TGF-β), vascular endothelial growth factor (VEGF), lipid mediators, and cytokines, may further promote remodeling, fibrosis, angiogenesis, and local inflammatory amplification [184]. Within this eosinophil-activated ocular microenvironment, EETs may provide an additional mechanism by organizing extracellular DNA, histones, and eosinophil granule proteins directly on the ocular surface [166,185]. These DNA and protein structures may localize antimicrobial and cytotoxic mediators at a vulnerable barrier site. If EET formation persists or becomes excessive, the same structures may concentrate histones and toxic granule proteins near epithelial and stromal tissues, contributing to barrier injury, inflammation, and possibly remodeling [166,185]. At present, the precise clinical relevance of EETs in VKC and AKC remains insufficiently defined, and further studies are needed to distinguish the contribution of EETosis from classical eosinophil degranulation and broader type 2 ocular surface inflammation. Direct human evidence for ocular EETosis is limited to a small descriptive study involving seven patients with VKC or AKC [185]. Mucus samples were examined from three patients and giant papilla tissue from five patients, with overlap between the groups. Although colocalization of extracellular DNA with MBP and citrullinated histone H3, together with ultrastructural changes, supports local EET formation, the study lacked healthy or disease controls and did not quantify EET burden. No associations with corneal injury, visual function, disease severity, recurrence, or treatment response were assessed. Therefore, the proposed role of EETs in shield ulcers, epithelial damage, and ocular remodeling remains biologically plausible but clinically unconfirmed.

### 6.6. EETosis in Gastrointestinal Mucosal Inflammation

Gastrointestinal mucosal diseases provide a biologically relevant setting for EETosis because the intestinal and esophageal mucosa are continuously exposed to dietary antigens, microbiota-derived signals, epithelial barrier stress, and persistent immune activation [186]. Under these conditions, eosinophil recruitment and activation may lead to extracellular DNA release at inflamed mucosal surfaces [59,110]. EETs may help retain microbial or inflammatory material locally, but sustained trap formation can also increase the extracellular DNA burden, concentrate cytotoxic eosinophil granule proteins, aggravate epithelial injury, and prolong mucosal inflammation [59]. This mechanism is particularly relevant in eosinophilic esophagitis (EoE) and Crohn’s disease, where eosinophil-rich inflammation overlaps with impaired barrier integrity, microbial stimulation, and chronic mucosal immune activation [21,110,187].

#### 6.6.1. EETs in Eosinophilic Esophagitis

Eosinophilic esophagitis is an eosinophil-predominant inflammatory disease of the esophageal mucosa, clinically associated with esophageal dysfunction and histologically defined by dense eosinophilic infiltration [110,188]. In active EoE, mitochondrial DNA-containing EETs are frequently detected, and their abundance correlates with the number of infiltrating tissue eosinophils, suggesting that EET formation reflects the intensity of local eosinophil-driven inflammation [58,110]. This relationship is closely linked to epithelial barrier dysfunction. Reduced expression of barrier-associated proteins, including filaggrin and lymphoepithelial Kazal-type-related inhibitor (LEKTI), indicates impaired mucosal protection. The negative correlation between LEKTI expression and EET formation further suggests that trap release becomes more prominent when epithelial integrity is weakened [110,189]. A compromised epithelial barrier may allow microbial products or pathogens to penetrate more deeply into the esophageal mucosa, providing additional local activating signals for eosinophils [110,190]. In parallel, epithelial antimicrobial programs are upregulated, including human beta-defensin-2, human beta-defensin-3, cathelicidin LL-37, and psoriasin, with psoriasin showing a positive association with EET formation [110]. These findings suggest that EETosis in EoE occurs as part of a broader epithelial barrier-stress response, rather than as an isolated eosinophil event. The local cytokine environment also appears permissive for EET formation, as increased TSLP, interleukin-25 (IL-25), interleukin-32 (IL-32), and IL-33 reflect epithelial stress, type 2 immune amplification, and ongoing mucosal inflammation [110,191]. Among these mediators, TSLP is especially relevant because its expression correlates with EET formation, supporting the possibility that epithelial-derived TSLP may directly or indirectly promote eosinophil activation and extracellular trap release in diseased esophageal tissue [80,110]. Under these conditions, EETs may help retain microbial or inflammatory material locally within extracellular DNA networks when the epithelial barrier is compromised [59,110]. However, persistent EET formation may also retain eosinophil granule proteins, extracellular DNA, and inflammatory mediators within the mucosa, thereby sustaining epithelial irritation, amplifying type 2 inflammation, and contributing to chronic tissue dysfunction. Direct evidence for EETosis in EoE is based mainly on a cross-sectional analysis of esophageal biopsies from 18 patients with active disease. Although EET formation correlated with tissue eosinophilia and inversely with LEKTI expression, these associations do not establish whether EETs cause barrier dysfunction or merely reflect more intense eosinophilic inflammation. EET burden has not been related quantitatively to dysphagia, endoscopic severity, fibrostenosis, treatment response, or recurrence. Therefore, the clinical and prognostic relevance of EETosis in EoE remains unvalidated. Furthermore, in EoE, EETs are best interpreted as part of the interaction between epithelial barrier injury, antimicrobial epithelial responses, type 2 cytokine signaling, and eosinophil activation, with potential protective value in barrier defense but pathogenic consequences when trap formation persists.

#### 6.6.2. EETs in Crohn’s Disease

Crohn’s disease is a chronic inflammatory disorder of the gastrointestinal tract in which mucosal barrier dysfunction, altered host–microbiota interactions, and persistent immune activation create conditions that may support eosinophil recruitment and activation [192]. Although Crohn’s disease is not classically defined as an eosinophilic disorder, eosinophils can accumulate in inflamed intestinal mucosa and may contribute to local injury through granule protein release, cytokine production, and possibly extracellular trap formation [187]. Early tissue observations support this possibility: in colon biopsies from patients with Crohn’s disease, as well as in bacterial gastrointestinal infection and schistosomiasis, extracellular DNA extrusions were detected in close association with eosinophil cationic protein-positive cells, suggesting that eosinophils can participate in DNA-based extracellular responses during intestinal inflammation [59]. In damaged mucosa, where epithelial disruption increases contact between eosinophils, microbial products, and invading organisms, EETs may help retain microbial or inflammatory material locally within extracellular DNA networks associated with eosinophil-derived proteins [58,59]. However, the functional significance of these structures remains uncertain. Persistent EET formation could also retain cytotoxic granule proteins, extracellular DNA, and inflammatory mediators within the intestinal wall, thereby amplifying epithelial injury, innate immune activation, and chronic tissue inflammation. Evidence for EETosis in Crohn’s disease remains limited to descriptive observations of eosinophil-associated extracellular DNA in human intestinal biopsies. These findings were not obtained from a dedicated quantitative Crohn’s disease cohort and were not related to disease activity, mucosal healing, complications, or treatment response. It also remains unclear whether the observed structures represented mitochondrial EET release, cytolytic nuclear EETosis, or mixed extracellular-trap activity. EETs should therefore be regarded as a preliminary histological observation rather than an established mechanism of Crohn’s disease progression.

### 6.7. EETosis in Sterile Vascular Inflammation and Thromboinflammatory Disease

Sterile vascular inflammation provides a relevant setting for EETosis because eosinophils, although less studied than monocytes, macrophages, neutrophils, and platelets, may contribute to endothelial activation, leukocyte recruitment, platelet responses, and vascular remodeling [193,194]. In this environment, activated eosinophils may undergo EETosis and deposit extracellular DNA, chromatin-rich material, and granule-derived proteins within the vascular wall or thrombotic lesions [194]. These extracellular structures could act as local pro-inflammatory and pro-thrombotic material by promoting endothelial dysfunction, retaining inflammatory cells, supporting platelet adhesion, and amplifying vascular injury. EET-mediated vascular deposition may be particularly relevant in atherosclerosis and arterial thrombosis, where persistent inflammatory activation and platelet–leukocyte interactions shape lesion progression, thrombus formation, and thrombus stability [194].

#### EETs in Atherosclerosis

Atherosclerosis is a chronic sterile inflammatory disease of the arterial wall in which endothelial activation, lipid accumulation, immune-cell recruitment, and vascular remodeling gradually generate unstable vascular lesions [195]. Although monocytes/macrophages, T cells, neutrophils, vascular cells, and platelets are usually considered the main cellular participants, eosinophils are increasingly recognized as additional vascular effector cells in selected inflammatory settings [194,196]. Their potential relevance is supported by clinical observations showing increased markers of eosinophil activation in cardiovascular disease, as well as experimental data suggesting that eosinophils can influence endothelial behavior and leukocyte recruitment [194,197,198,199]. Activated eosinophils may promote a more adhesive endothelial phenotype through eosinophil-derived mediators and extracellular trap-associated components [194]. For example, eosinophil-derived cationic proteins can upregulate endothelial intercellular adhesion molecule-1 (ICAM-1/CD54) expression in epithelial inflammatory models, while endothelial ICAM-1 is a key mediator of leukocyte adhesion to the vascular wall [197]. Eosinophil activity has also been linked to increased endothelial von Willebrand factor exposure and enhanced platelet adhesion, suggesting that eosinophils may help create a proadhesive vascular surface where endothelial activation, leukocyte recruitment, and platelet attachment reinforce one another [194].

Within this vascular inflammatory environment, platelet–eosinophil interaction may serve as an important trigger for EETosis [194,196]. Activated platelets can stimulate eosinophils more effectively through direct cell–cell contact than through soluble mediators alone, and this interaction appears to involve P-selectin-dependent adhesion [194,200]. Once activated by platelets, eosinophils may release extracellular DNA structures decorated with MBP, generating MBP-positive EETs within vascular lesions or intravascular inflammatory material [194]. The presence of eosinophil granule proteins on extracellular DNA helps distinguish these structures from neutrophil-derived extracellular traps [71,194]. This distinction is biologically important because MBP indicates eosinophil origin and may also enhance platelet activation and aggregation. In this way, EETosis may contribute to a reciprocal thromboinflammatory loop: activated platelets stimulate eosinophils, eosinophils release MBP-positive extracellular DNA networks, and these EET-derived structures further promote platelet activation, extracellular DNA accumulation, and inflammatory persistence within the vascular lesion. However, the relevance of this mechanism in human atherosclerosis still requires careful interpretation. The major strength of the available evidence is the combined use of isolated human eosinophils, eosinophil-deficient mice, experimental arterial injury, and examination of human coronary thrombi. However, the causal evidence for EETs is derived predominantly from mouse thrombosis and atherosclerosis models. Detection of MBP-positive extracellular traps in human thrombi confirms their presence but does not establish that they initiate atherosclerosis or independently determine thrombus size, stability, or cardiovascular outcome. No prospective human study has related vascular EET burden to plaque progression, myocardial infarction, stroke, recurrent thrombosis, or response to antithrombotic treatment.

### 6.8. EETosis in Immune-Mediated Diseases

In immune-mediated diseases, activated eosinophils may contribute to tissue injury by releasing cytokines, undergoing degranulation, and depositing extracellular DNA–protein structures through EETosis [70]. These EET-derived structures contain extracellular DNA, eosinophil granule proteins, and inflammatory mediators that may sustain local immune activation, prolong tissue inflammation, and support fibroinflammatory remodeling [34]. This mechanism may be particularly relevant in chronic immune-mediated disorders characterized by eosinophil-rich infiltrates, fibrosis, and an incompletely defined role of eosinophils, such as IgG4-related disease [201].

#### EETs in IgG4-Related Disease

IgG4-related disease is a systemic fibroinflammatory disorder characterized by tumefactive lesions or diffuse enlargement of affected organs, dense lymphoplasmacytic inflammation, fibrosis, and variable multiorgan involvement [202]. Although the disease is usually interpreted through IgG4-positive plasma cells, T-cell activation, and fibroinflammatory remodeling, eosinophils are also clinically relevant [201]. Peripheral eosinophilia is present in a substantial proportion of patients, while eosinophilic infiltration within affected tissues appears even more frequent [201,203]. Increased eosinophil counts have been associated with disease activity, multiorgan involvement, relapse risk, and fibrotic manifestations, suggesting that eosinophils may contribute to inflammatory persistence and tissue remodeling in selected patients [203,204]. In this setting, EETosis provides a plausible mechanism through which activated eosinophils could deposit persistent extracellular inflammatory material within affected organs [34,202]. Cytolytic EETosis can release extracellular DNA, intact free eosinophil granules, and granule-derived proteins into tissues, where these structures may persist after eosinophil death and continue to provide cytotoxic, immunomodulatory, and potentially profibrotic signals. This may be particularly relevant in IgG4-related disease, where chronic fibroinflammatory lesions are shaped by long-lasting interactions between immune cells, stromal cells, extracellular matrix, and tissue-resident inflammatory mediators [34,202]. Because IgG4-related lesions may contain eosinophil-rich infiltrates and cytolytic eosinophil material, galectin-10 and Charcot–Leyden crystals are also relevant to this interpretation [158,203]. Galectin-10 is abundant in eosinophils and may be released during EETosis or other forms of eosinophil cytolytic activation; after release, it can crystallize into Charcot–Leyden crystals [158]. The presence of these crystals in IgG4-related lesions supports ongoing eosinophil cytolysis and extracellular deposition of eosinophil-derived material [203]. In this way, galectin-10 and Charcot–Leyden crystals may represent persistent eosinophil-derived inflammatory deposits that could contribute to local immune activation and fibroinflammatory remodeling. This suggests a possible feed-forward process in which eosinophil accumulation promotes cytolysis, EETosis, and crystal formation, while extracellular eosinophil-derived material sustains tissue inflammation and remodeling. However, this model remains partly inferential. Eosinophilia and Charcot–Leyden crystals support active eosinophil involvement, but they do not prove that EETosis is a primary driver of IgG4-related disease. Further tissue-based studies using extracellular DNA, eosinophil granule proteins, galectin-10, eosinophil-specific trap markers, and spatial colocalization analyses are needed to clarify whether EETs are mechanistic contributors, markers of disease activity, or secondary consequences of eosinophil-rich fibroinflammation.

The distinction between evidence for eosinophilia and evidence for EETosis is particularly important in IgG4-related disease. Clinical associations between eosinophilia, greater disease activity, and multiorgan involvement were obtained from a cohort of 425 patients, but EETs were not measured in that study. In contrast, direct tissue evidence of EETosis and Charcot–Leyden crystals is currently limited to a single case report. These findings demonstrate that EETosis can occur in eosinophil-rich IgG4-related lesions but do not establish its prevalence, causal role in fibrosis, association with relapse, or response to treatment.

Taken together, pathological EET formation should be viewed as more than a bystander feature of eosinophil-rich inflammation. When EETs are excessive, persistent, or inefficiently cleared, their extracellular DNA scaffold, associated histones, granule proteins, galectin-10-related material, and cell-free granules may sustain epithelial injury, mucus retention, vascular inflammation, thrombosis, and fibroinflammatory remodeling. This concept is particularly relevant across mucosal, pulmonary, cutaneous, gastrointestinal, vascular, and systemic eosinophilic disorders, where EETs may help explain why inflammation persists even after the initial trigger has weakened. The major disease-specific implications of pathogenic EETosis are summarized in Table 4.

### 6.9. EETs in Cancer

Eosinophils are increasingly recognized as functionally plastic immune cells within the tumor microenvironment, although their role in cancer remains more difficult to define than in allergic or eosinophilic inflammatory diseases [205]. Tumor-associated tissue eosinophilia refers to the accumulation of eosinophils within intratumoral or peritumoral inflammatory infiltrates, and its prognostic meaning varies considerably across tumor types [206,207]. In some malignancies, increased eosinophil infiltration or higher peripheral eosinophil counts have been associated with improved survival, supporting the possibility that eosinophils may contribute to antitumor immunity [206,208,209]. In other settings, however, eosinophils appear to correlate with tumor progression, immune suppression, stromal remodeling, or poorer clinical outcome [210,211]. This variability suggests that eosinophils should not be interpreted as uniformly protective or harmful in cancer [205]. Their function depends on tumor type, viral status, cytokine context, local immune composition, and the activation state of eosinophils themselves [205]. Tumor cells and stromal elements may promote eosinophil recruitment through mediators such as IL-33, high-mobility group box 1 (HMGB1), CCL11, CCL24, C-C motif chemokine ligand 5 (CCL5), and C-C motif chemokine ligand 3 (CCL3). Recruited eosinophils may then modulate the tumor microenvironment (TME) by releasing granule proteins, cytokines, lipid mediators, and potentially EETs [211,212]. In this context, EETosis may represent an additional effector pathway through which eosinophils communicate with tumor cells, stromal cells, and other immune populations [211].

#### 6.9.1. Potential Mechanisms Linking EETs to Tumor Progression, Immune Modulation, Metastasis, and Thrombosis

Current evidence supports several potential mechanisms through which EETs could influence tumor behavior, although the strength of evidence differs substantially among individual processes. Direct evidence consists mainly of histological or ultrastructural identification of EET-like structures in selected human tumors and, more recently, experimental demonstration of EET formation in an eosinophil-rich murine lung carcinoma model. In contrast, several proposed effects on metastatic dissemination and cancer-associated thrombosis are currently supported predominantly by transcriptomic associations, established functions of eosinophils and their granule proteins, or mechanistic parallels with NETs. This distinction is important because extracellular DNA detected in tumor tissue cannot be attributed to eosinophils without its colocalization with eosinophil-specific proteins and appropriate exclusion of NETs, cellular necrosis, and other sources of extracellular DNA [211,213,214].

The antitumor potential of eosinophils is biologically plausible because these cells can release cytotoxic granule proteins, membrane-active mediators, cytokines, and reactive oxygen species that may directly damage malignant cells or promote local immune activation [205]. EETs could theoretically reinforce these effects by forming extracellular DNA networks associated with eosinophil granule proteins, thereby retaining and concentrating cytotoxic mediators in close proximity to tumor cells [206,207]. Such an effect may be particularly relevant in non-viral tumors, in which tumor-associated eosinophilia has more frequently been associated with favorable clinical outcomes [206,207]. Close spatial contact between eosinophils, extracellular DNA, eosinophil granules, and injured tumor cells has been observed in selected ultrastructural studies, providing morphological support for the possibility of locally directed eosinophil activity. However, these observations do not yet establish that EET formation improves tumor-cell killing or enhances antitumor immunity in vivo.

Once eosinophils have entered the tumor microenvironment, EET formation may retain extracellular DNA together with MBP, ECP, EDN, EPX, and other eosinophil-derived products within spatially restricted tumor regions. This arrangement may have opposing consequences. High local concentrations of membrane-active and cytotoxic proteins could contribute to tumor-cell injury, particularly when eosinophils establish close contact with malignant cells. Conversely, persistent DNA–protein aggregates could prolong inflammatory signaling, damage surrounding non-malignant cells, activate endothelial and stromal populations, and maintain a tissue environment favorable to chronic inflammation and remodeling. Therefore, the biological effects of EETs are likely to depend not only on their abundance but also on their anatomical localization, persistence, protein composition, and clearance by extracellular nucleases.

The same extracellular trap biology may therefore support tumor-promoting mechanisms. EET-like structures could contribute to extracellular matrix remodeling, chronic inflammatory signaling, angiogenesis, macrophage polarization, regulatory T-cell recruitment, and immune suppression [211]. Eosinophil granule proteins may also exert context-dependent effects on tumor cells [215,216]. For example, EPX has been reported to interact with human epidermal growth factor receptor 2 (HER2) in melanoma cells and promote their proliferation, indicating that eosinophil-derived products do not invariably act as antitumor effectors [217]. Thus, EETs should be viewed as extracellular DNA–protein structures with dual potential: under some conditions, they may support tumor-cell injury and immune activation, whereas in others they may promote inflammation, adhesion, tissue remodeling, immune suppression, and tumor progression.

Pan-cancer transcriptomic analyses have added a broader, although indirect, layer to this interpretation. EET-related gene signatures vary substantially among tumor types and appear to be higher in several malignancies that generally have unfavorable outcomes, including pancreatic adenocarcinoma, head and neck squamous cell carcinoma, lung squamous cell carcinoma, esophageal carcinoma, and glioblastoma [211]. In contrast, tumors with generally longer expected survival, including prostate adenocarcinoma, thyroid carcinoma, and kidney chromophobe, tend to have lower EET-related scores [211]. The marked difference between glioblastoma and lower-grade glioma further suggests that EET-associated transcriptional activity may increase with tumor aggressiveness, inflammatory activation, or extensive remodeling of the tumor microenvironment.

High EET-related signatures have been associated with coordinated immune and stromal programs, including interleukin-17 (IL-17) signaling, IL-6–Janus kinase (JAK)–signal transducer and activator of transcription 3 (STAT3) signaling, interleukin-2 (IL-2)–STAT5 signaling, and interferon-γ (IFN-γ) responses [211]. Additional associations include extracellular matrix (ECM) remodeling, M2-like macrophage enrichment, regulatory T-cell (Treg) differentiation, and increased expression of multiple immune checkpoint molecules [211]. At the same time, tumors with higher EET-related activity may contain fewer cytotoxic CD8-positive T cells and activated natural killer (NK) cells, supporting a possible relationship between EET-associated programs and an immunosuppressive tumor microenvironment [211].

More detailed quantitative analysis has shown particularly strong positive correlations between the EET-related score and extracellular matrix disassembly (R = 0.802), macrophage differentiation (R = 0.818), IL-17 signaling (R = 0.801), and Treg-related differentiation programs (R = 0.794) [211]. By comparison, correlations with angiogenesis (R = 0.304) and tumor-cell-cycle activity (R = 0.446) were weaker [211]. This pattern suggests that EET-related molecular activity may be more closely linked to stromal remodeling and immune regulation than to direct stimulation of malignant-cell proliferation. Nevertheless, these correlations do not demonstrate that EETs themselves initiate these biological processes.

Immune-cell deconvolution in the same pan-cancer analysis showed a reduced M1/M2 macrophage ratio in tumors with high EET-related scores, together with lower abundance of activated NK cells and CD8-positive T cells [211]. Experimental observations provided partial biological support for this association. Marked EET formation was detected in Lewis lung carcinomas established in IL-5-transgenic hypereosinophilic mice, in which CD206-positive, inducible nitric oxide synthase-negative M2-like macrophages were also prominent [211]. These results support an association among eosinophilia, EET formation, and M2-like macrophage accumulation. However, they do not prove that EETs directly induce macrophage polarization, because sustained IL-5 expression and eosinophilia may also influence macrophages through soluble cytokines, lipid mediators, granule proteins, or other EET-independent mechanisms.

Granule-associated genes may also contribute to the prognostic associations observed in pan-cancer datasets. Higher expression of *RNASE2* and *RNASE3*, encoding EDN and ECP, respectively, has been linked with increased event risk and shorter survival in some analyses [211]. Nevertheless, increased expression of these genes may indicate eosinophil infiltration, eosinophil activation, degranulation, or broader granulocytic inflammation rather than actual extracellular trap formation. EET-related scores should therefore be interpreted as molecular indicators of eosinophil- and EET-associated inflammatory activity, rather than direct measurements of EET burden in tumor tissue.

EETs could also influence metastatic dissemination by providing extracellular surfaces that retain tumor cells or modify their interactions with the surrounding matrix. A well-defined mechanism has been established for NET-derived DNA: coiled-coil domain-containing protein 25 (CCDC25) on tumor cells recognizes extracellular NET DNA and activates the integrin-linked kinase–β-parvin pathway, thereby promoting cytoskeletal remodeling, adhesion, and cancer-cell motility [215]. Because EETs also contain extracellular DNA, engagement of CCDC25 or other extracellular DNA-sensing receptors by EET-derived DNA is biologically plausible. However, direct binding of EET-derived DNA to CCDC25 and subsequent induction of tumor-cell migration have not yet been experimentally demonstrated. CCDC25 should therefore be presented as a potential mechanistic analogy rather than an established receptor for EETs.

Similarly, the association between EET-related scores and extracellular matrix disassembly suggests a possible role in local invasion. EET-associated proteases and granule proteins could potentially alter matrix proteins, expose cryptic adhesion sites, or modify interactions among malignant cells, stromal cells, and the vascular endothelium. Persistent extracellular DNA networks might also retain chemokines, cytokines, and matrix-modifying enzymes within invasive tumor regions. Nevertheless, there is currently no direct evidence that EETs capture circulating tumor cells, promote their extravasation, establish premetastatic niches, or reactivate dormant malignant cells in the manner described for NETs.

A potential contribution of EETs to cancer-associated thrombosis should also be considered. Outside the cancer setting, interactions between platelets and eosinophils can induce EET formation, and EETs have been identified in both human and experimental thrombi. MBP deposited on EETs enhances platelet activation and thrombus stability, whereas inhibition of eosinophils or EET formation reduces experimental thrombosis [194]. Eosinophils may additionally contribute to coagulation through tissue factor expression, endothelial activation or injury, and interference with thrombomodulin-dependent anticoagulant mechanisms by released granule proteins. These observations provide a plausible biological basis through which EETs might amplify the hypercoagulable state of eosinophil-rich malignancies.

In Hodgkin lymphoma, extracellular traps have been detected in association with fibrosis and tissue factor-positive endothelium, suggesting the coexistence of granulocyte trap formation, inflammatory remodeling, and a local procoagulant phenotype [213]. However, these findings do not demonstrate that EETs independently initiate coagulation or cause clinically apparent venous thromboembolism. At present, most experimental and clinical evidence connecting extracellular traps with cancer-associated thrombosis concerns NETs rather than EETs. Direct correlations between histologically validated EET burden and thrombotic outcomes in patients with cancer have not yet been reported.

Taken together, EET biology may represent a context-dependent interface between eosinophilic inflammation and cancer progression. In selected tumors, EET-associated localization of cytotoxic eosinophil products may contribute to malignant-cell injury and immune activation. In other settings, persistent or inadequately cleared EETs may support stromal remodeling, M2-like macrophage accumulation, regulatory immune programs, tumor-cell adhesion and migration, platelet activation, and vascular complications. Future studies should quantify histologically validated EETs rather than relying solely on eosinophil-related gene signatures and should correlate EET burden with tumor molecular subtype, immune-cell composition, metastatic recurrence, venous thromboembolism, treatment response, and patient survival.

#### 6.9.2. EETs in Head and Neck Squamous Cell Carcinoma

Head and neck squamous cell carcinoma (HNSCC) is an aggressive epithelial malignancy arising from the mucosal surfaces of the upper aerodigestive tract, including the oral cavity, pharynx, and larynx. Because these tumors develop at barrier sites exposed to microorganisms, irritants, epithelial injury, and local immune activation, their tumor microenvironment is strongly shaped by inflammation [218]. This makes HNSCC a relevant setting for considering eosinophils and EET-related biology, although direct evidence of organized EETosis in HNSCC tissue remains limited [219]. Tumor-associated eosinophils have been identified within HNSCC lesions, but their prognostic significance varies across molecular and immunological subgroups [210]. In some settings, eosinophil infiltration has been associated with more favorable outcomes, whereas in others this association is absent or less clear [210,220]. Once recruited into the tumor microenvironment, eosinophils may influence tumor behavior through granule proteins, cytokines, chemokines, lipid mediators, and potentially extracellular DNA-based structures [221]. If EETs are formed in HNSCC, they could localize eosinophil-derived cytotoxic mediators near malignant cells, but they could also contribute to persistent inflammation, extracellular matrix remodeling, immune regulation, and tumor-cell migration [219].

A potentially relevant molecular link involves *SPINK5*, which encodes lympho-epithelial Kazal-type-related inhibitor (LEKTI) [222]. LEKTI contributes to epithelial protease–antiprotease balance, barrier homeostasis, inflammation, and tissue remodeling, while *SPINK5* downregulation in HNSCC has been associated with increased tumor-cell proliferation and invasion [223]. Its association with EET-associated molecular patterns may therefore reflect overlapping processes involving epithelial barrier dysregulation, altered protease activity, tumor invasion, and eosinophil-associated inflammatory activity [219]. In HNSCC, EET-related molecular patterns should not be taken as direct proof of extracellular trap formation. Instead, they may represent indirect markers of eosinophil activation, epithelial remodeling, and immune–stromal changes within the tumor microenvironment [219]. These molecular findings suggest a connection not only with immune surveillance, but also with proliferative, migratory, and tumor-promoting remodeling processes. Evidence for EET-related biology in HNSCC is currently based mainly on retrospective bioinformatic analysis of public transcriptomic datasets. The identification of SPINK5 as a candidate regulator was derived from an EET-related gene set and prognostic modeling, without direct visualization of EETs in HNSCC tissue or functional manipulation of SPINK5 in eosinophils. In addition, studies linking tumor-associated eosinophilia to angiogenesis and metastasis measured eosinophil infiltration rather than EET formation. SPINK5 should therefore be described as a candidate EET-associated gene, not as a confirmed regulator of EETosis in HNSCC [211].

#### 6.9.3. EETs in Endometrial Cancer

Endometrial cancer is one of the most common gynecological malignancies and shows particularly unfavorable clinical behavior in advanced, recurrent, or treatment-resistant disease [224]. Because tumor progression is influenced by inflammatory signaling, immune-cell infiltration, and remodeling of the tumor microenvironment, EET-related molecular programs may be relevant in this malignancy [211,225]. Recent analyses have shown that genes linked to EET-related regulation are differentially expressed in endometrial cancer tissue compared with normal endometrium, with 108 EET-related regulators showing altered expression [226]. This finding does not prove that EETosis occurs within endometrial tumors, but it suggests that transcriptional programs associated with eosinophil activation, extracellular trap-related inflammation, and immune remodeling may be altered in the tumor setting. Among these genes, *S100A9* has emerged as a potentially important candidate [226]. *S100A9* is not specific for EETosis, but its increased expression in endometrial cancer tissue and its association with tumor progression, immune infiltration, and poorer clinical outcome suggest a link with tumor-promoting inflammation [226,227]. At the signaling level, *S100A9* may contribute to NF-κB activation, a key inflammatory pathway involved in cytokine production, tumor-cell survival, immune regulation, and cancer progression [226,228]. In this tumor context, *S100A9* may represent one component of a broader inflammatory profile associated with EET-related activity, immune remodeling, and stromal changes within the tumor microenvironment [226]. However, current evidence remains indirect and mainly supports an association between EET-related gene expression, *S100A9* activity, immune infiltration, and unfavorable disease biology, rather than direct visualization or functional confirmation of EETs in endometrial cancer tissue. The endometrial cancer findings should also be distinguished from direct evidence of EETosis. The study identified 108 differentially expressed EET-related regulators and developed a prognostic model with a reported C-index of 0.864, but it did not visualize or quantify EETs in tumor tissue. Functional experiments examined *S100A9* signaling in endometrial cancer cell lines rather than EET formation by eosinophils. Because *S100A9* is expressed by several myeloid and tumor-associated cell populations, its prognostic value cannot be considered specific evidence of EET activity. These results identify an EET-associated molecular signature but do not demonstrate that EETs cause tumor progression or treatment resistance.

#### 6.9.4. EETs in Gastric

Gastric cancer provides a tumor setting in which eosinophil extracellular trap formation has been described within the local tumor microenvironment, although the available evidence remains limited [207,229]. In one reported case, tumor-associated eosinophils were observed at different morphological stages of EETosis, ranging from early and intermediate changes to advanced cytolytic forms [214]. This staged pattern suggests that EET formation in gastric tumor tissue may occur as a progressive process involving nuclear remodeling, cytoplasmic alteration, and eventual extracellular trap release, rather than as a single abrupt event [214]. The predominance of early and intermediate EETotic eosinophils over fully advanced cytolytic cells further supports the possibility of ongoing eosinophil activation within the tumor microenvironment [214]. Ultrastructural findings, including chromatin decondensation, plasma membrane disruption, granule discharge, and extracellular trap formation, provide additional morphological support for organized EETosis in this setting [214]. These observations suggest that local EETosis may represent one way in which tumor-associated eosinophils interact with malignant cells, stromal elements, and inflammatory components of gastric cancer tissue. However, the functional significance of this process remains uncertain. Based on case-level evidence alone, it is not possible to determine whether EETosis contributes to antitumor cytotoxicity, inflammatory remodeling, tumor progression, or simply reflects intense eosinophil activation within the tumor microenvironment. The principal strength of the gastric cancer studies is the ultrastructural demonstration of sequential nuclear and membrane changes compatible with EETosis. However, the available evidence is limited to four selected gastric cancer cases, and clear stages of EETosis were identified in only one case. The remaining three cases predominantly showed non-ETotic eosinophil cytolysis. These observations establish that EETosis can occur in gastric cancer tissue but do not indicate its prevalence or demonstrate an antitumor effect. No quantitative association with tumor stage, recurrence, treatment response, or survival has been reported.

#### 6.9.5. EETs in Hodgkin Lymphoma

Classic Hodgkin lymphoma (cHL) is a malignant lymphoid disease in which Hodgkin and Reed–Sternberg cells are embedded within a highly reactive inflammatory microenvironment composed of diverse immune and stromal cells [230]. This architecture is biologically important because malignant cells represent only a fraction of the tumor mass, while the surrounding infiltrate strongly influences tumor behavior, immune escape, stromal remodeling, and clinical heterogeneity. Eosinophils may be prominent in selected cases of cHL, suggesting the presence of tumor-derived or microenvironmental signals that support eosinophil recruitment, survival, and activation [231]. In one eosinophil-rich lymphoma case, immunohistochemical analysis showed extracellular trap-like structures in areas densely infiltrated by eosinophils, supporting the possibility that EET formation can occur within eosinophil-dominant tumor tissue [213]. These EET-like structures may reflect local eosinophil activation, with extracellular deposition of DNA and eosinophil-derived products within the inflammatory tumor stroma [213]. Pathophysiologically, this material could contribute to local immune activation, retention of inflammatory mediators, stromal remodeling, and communication between eosinophils and other components of the Hodgkin lymphoma microenvironment [213]. However, the functional significance of these findings remains uncertain. Because current evidence is mainly histological and case-based, EET-like material in eosinophil-rich cHL should be interpreted as suggestive evidence of local eosinophil extracellular trap formation rather than proof of an active tumor-modifying EET pathway. Evidence for EETosis in classic Hodgkin lymphoma remains particularly limited. Although extracellular traps were frequently detected in fibrotic nodular-sclerosis lesions, most were classified as NETs, whereas abundant EETs were demonstrated in only one eosinophil-rich case. The coexistence of EETs, fibrosis, and tissue factor-positive endothelium does not establish that EETs cause stromal remodeling or thrombosis. EET burden has not been quantitatively related to lymphoma stage, venous thromboembolism, treatment response, relapse, or survival.

Collectively, EETs in cancer should be interpreted as a context-dependent manifestation of eosinophil activity within the tumor microenvironment. In selected settings, extracellular DNA structures associated with eosinophil granule proteins may concentrate cytotoxic mediators near malignant cells and contribute to antitumor immune activity. Conversely, persistent or excessive EET material may support chronic inflammation, matrix remodeling, immune suppression, angiogenesis, tumor-cell adhesion, and migration. Thus, current evidence points to a dual and tumor-specific role of EET biology, while direct histological and functional confirmation of organized EETosis remains limited in most cancers. The main tumor-related implications are summarized in Table 5.

## 7. Future Therapeutic Perspectives and Translational Strategies Targeting EETosis

The identification of eosinophil extracellular traps in human airways and inflammatory secretions has provided a rationale for therapeutic strategies directed either at dismantling established EETs or preventing their excessive formation [76,142]. However, selective targeting of pathological EETosis remains challenging because extracellular traps may also contribute to microbial immobilization and host defense, while currently available interventions have limited cellular specificity [54]. Additional barriers to clinical translation include nuclease instability, treatment cost, incomplete tissue penetration, off-target effects, and the potential toxicity of agents that interfere with oxidative signaling, histone modification, or extracellular DNA sensing.

Recombinant human deoxyribonuclease I, also known as rhDNase or dornase alfa, is the most clinically advanced intervention aimed at degrading the extracellular DNA backbone of inflammatory traps [142,232,233,234]. In an ovalbumin-induced murine asthma model, intranasal rhDNase reduced extracellular DNA traps and improved airway resistance, supporting a direct contribution of extracellular DNA to airway obstruction [142]. Nevertheless, a randomized placebo-controlled trial involving 121 children with moderate-to-severe acute asthma found that a single 5 mg nebulized dose of rhDNase did not significantly improve the clinical asthma score, oxygen requirement, or duration of bronchodilator treatment [232]. A subsequent pilot trial involving 50 adults with severe acute asthma also found no significant overall improvement in FEV_1_ or clinical status following nebulized rhDNase doses of 2.5–7.5 mg [233]. In a more recent biomarker-enriched crossover trial, 10 days of treatment with 2.5 mg rhDNase substantially reduced sputum extracellular DNA in patients with asthma but did not significantly improve respiratory symptoms, lung function, or most inflammatory outcomes [234]. Importantly, these trials did not specifically enroll patients with eosinophilic asthma, directly demonstrate EET-rich mucus plugs, or use eosinophil-specific DNA–granule protein complexes for patient selection.

The discrepancy between biochemical target engagement and limited clinical benefit may reflect the complex structure of airway mucus plugs [234,235]. In addition to extracellular DNA, asthma plugs may contain MUC5AC, MUC5B, epithelial material, eosinophil granule proteins, galectin-10, Charcot–Leyden crystals, and variable numbers of eosinophils and neutrophils [235]. DNase-mediated cleavage of extracellular DNA may therefore be insufficient when mucin hyperconcentration and disulfide-dependent mucin cross-linking continue to maintain secretion viscosity and luminal obstruction [236,237,238]. This provides a rationale for combining rhDNase with mucin-reducing agents that target a structurally distinct component of pathological secretions [236,237,238]. In cystic fibrosis sputum, combined treatment with rhDNase and the thiol-based mucolytic Nacystelyn reduced viscoelasticity more effectively than either agent alone in selected rheological measurements [236]. An additional study demonstrated additive effects of dornase alfa and Nacystelyn on both sputum viscoelasticity and mucociliary transportability [237]. The thiol-modified saccharide MUC-031 also rapidly reduced sputum viscoelasticity ex vivo and decreased mucus plugging, airway inflammation, and mortality in βENaC-overexpressing mice [238]. However, these studies were performed in cystic fibrosis sputum or experimental muco-obstructive disease, and combined DNase–mucolytic treatment has not yet been evaluated in patients with EET-rich eosinophilic asthma.

Preventing continuing EET formation may complement the removal of extracellular traps that have already accumulated [79]. Reactive oxygen species participate in several experimentally defined forms of EETosis, making oxidative signaling a potential upstream therapeutic target [79]. In an ovalbumin-induced asthma model, both N-acetylcysteine and diphenyleneiodonium reduced airway EET formation, eosinophil peroxidase activity, goblet-cell hyperplasia, inflammatory cytokine production, NF-κB activation, and pulmonary oxidative stress [79]. N-acetylcysteine additionally improved indices of mitochondrial energy metabolism, suggesting that antioxidant treatment may simultaneously influence oxidative signaling, eosinophil activation, and mucus properties [79]. However, these effects were demonstrated in an experimental asthma model and cannot be attributed exclusively to the inhibition of EET formation [79]. Broad inhibition of ROS or NADPH oxidase may also impair physiological redox signaling and antimicrobial defense, limiting the feasibility of indiscriminate systemic treatment [59,79]. Furthermore, EETosis is mechanistically heterogeneous, as selected stimuli such as *Aspergillus fumigatus* can induce EET release through pathways that are not primarily dependent on NADPH oxidase or PAD4 [108].

PAD4-mediated histone citrullination and chromatin decondensation represent additional targets, particularly in cytolytic nuclear EETosis [82,86]. In isolated human eosinophils, inhibition of the IRE1α–XBP1s pathway or PAD4 reduced arachidonic acid-induced EET formation, histone H3 citrullination, degranulation, and Charcot–Leyden crystal formation [82]. Dexamethasone and hydrocortisone did not prevent arachidonic acid-induced EET release or galectin-10 crystallization in the same experimental system, suggesting that selected forms of EETosis may persist despite glucocorticoid exposure [82]. The monoclonal antibody CIT-013, which recognizes citrullinated histones H2A and H4, inhibited EET release from human eosinophils with a reported half-maximal inhibitory concentration of approximately 2.5 nM [86]. CIT-013 did not measurably suppress eosinophil adhesion, degranulation, superoxide production, or chemokine expression, suggesting that inhibition of chromatin expulsion may be separated from selected physiological eosinophil responses [86]. Nevertheless, CIT-013 remains a preclinical intervention, and inhibition of EET release did not prevent galectin-10 secretion, indicating that other potentially pathogenic eosinophil products may persist despite suppression of chromatin release [86].

Nanomedicine-based approaches may improve extracellular-trap targeting by increasing tissue retention, protecting nucleases from degradation, and combining DNA removal with antioxidant or anti-inflammatory activity [130,239,240,241]. Nanoparticles can be engineered to recognize extracellular DNA, histones, or other molecular components of NETs and EETs, while DNase-conjugated particles may permit localized degradation of extracellular DNA at sites of excessive trap formation [130,239,240,241]. A linear polyglycerol-amine-coated TiS_2_ nanoplatform reduced cell-free DNA in nasal secretions from patients with eosinophilic chronic rhinosinusitis, inhibited cell-free DNA-induced TLR9 activation and EET formation in vitro, and attenuated eosinophilic inflammation in a mouse model [241]. These findings suggest that extracellular DNA scavenging may interrupt a self-amplifying cell-free DNA–TLR9–EET inflammatory pathway without requiring systemic eosinophil depletion [241].

A related strategy was developed for recalcitrant otitis media with effusion using an ultrathin two-dimensional nanoscavenger designated C-TA_H [130]. The platform was produced by modifying copper indium thiophosphate nanosheets with tannic acid and histone-binding aptamers, thereby combining DNA binding, histone recognition, antioxidant activity, and extracellular-trap sequestration [130]. Tannic acid can form stable complexes with DNA and suppress dsDNA-induced inflammatory signaling, while tannic acid-based nanoformulations may additionally scavenge ROS and enhance endogenous antioxidant activity [13]. C-TA_H bound the double-stranded DNA of NETs and EETs, reduced ROS levels, and inhibited bacterial growth in experimental assays [130]. In an ovalbumin-induced rat model of otitis media with effusion, C-TA_H reduced CitH3–DNA and ECP–DNA complexes, type 2 cytokines, TLR9 and NF-κB activation, mucous exudation, and local inflammatory changes [130]. Treatment also improved acoustic immittance and auditory brainstem response measurements, supporting the feasibility of locally targeting mixed NET/EET-associated inflammation [130]. However, the platform was evaluated in an experimental model rather than in patients, and its long-term retention, degradation, ototoxicity, systemic absorption, and repeated-dose safety remain unknown.

Other nanoplatforms combine the prevention of new trap formation with the degradation of traps that are already present. Genetically engineered cellular nanovesicles have been developed to deliver DNase I selectively to NET-rich sites in experimental acute lung injury, improving pulmonary nuclease delivery and extracellular-trap clearance [239]. A dual-function platform consisting of DNase I conjugated to cerium oxide nanoparticles combined extracellular DNA degradation with antioxidant enzyme-mimetic activity [240]. In human neutrophil experiments, the cerium oxide component reduced intracellular ROS and inhibited new extracellular-trap formation, while surface-associated DNase I retained the capacity to degrade pre-existing traps [240]. This combined upstream and downstream strategy is conceptually relevant to EET-associated airway inflammation, but these platforms were developed primarily for NETs and have not been validated in primary human eosinophils or EET-rich asthma mucus. Their translation will require evaluation of aerosol stability, regional airway deposition, mucus penetration, preservation of DNase activity, nanoparticle clearance, immunogenicity, and repeated-dose pulmonary toxicity.

Improving extracellular-trap clearance will probably require simultaneous targeting of extracellular DNA and DNA-associated inflammatory components. DNase can disrupt the DNA backbone but may not neutralize extracellular histones, major basic protein, eosinophil peroxidase, eosinophil cationic protein, eosinophil-derived neurotoxin, galectin-10, or secretion-competent extracellular granules. Moreover, DNA fragmentation could release biologically active proteins that were previously retained within the extracellular network. Antibodies directed against the galectin-10 crystallization interface have dissolved pre-existing Charcot–Leyden crystals in patient-derived mucus and reduced crystal-induced type 2 inflammation in a humanized mouse model [242]. These findings support combination strategies in which DNase-mediated DNA degradation is accompanied by mucin reduction, ROS modulation, histone neutralization, or removal of galectin-10 crystals and eosinophil granule proteins.

Biological therapies targeting IL-5 or the IL-5 receptor may indirectly reduce EET-generating capacity by decreasing eosinophil priming, survival, recruitment, and tissue accumulation [136]. IL-5 antagonism reverses several features of eosinophil priming and activation in severe eosinophilic asthma, but no clinical study has demonstrated that the therapeutic benefit of anti-IL-5 treatment is specifically mediated through a reduction in EET burden [136]. Eosinophil depletion should also not be considered equivalent to the clearance of extracellular chromatin, free granules, galectin-10 crystals, or other EET-associated material already deposited in airway tissues and secretions [136]. Future trials of mepolizumab, reslizumab, benralizumab, dupilumab, tezepelumab, and related therapies should therefore include direct measurements of EET-associated biomarkers before and after treatment.

Future clinical studies should employ precision-treatment designs and prioritize patients with objectively demonstrated EET-rich disease rather than unselected asthma populations. Candidate enrichment biomarkers include sputum or secretion extracellular DNA, DNA–eosinophil peroxidase, DNA–eosinophil cationic protein or DNA–major basic protein complexes, citrullinated histone–DNA complexes, galectin-10, Charcot–Leyden crystals, and microscopy-confirmed EET structures. In eosinophilic airway disease, these markers should be assessed together with sputum viscoelasticity, mucociliary transportability, computed tomography-defined mucus-plug scores, FEV_1_, small-airway function, symptoms, exacerbations, and rescue medication use. Future proof-of-concept trials could compare rhDNase alone with rhDNase combined with mucin-reducing agents, antioxidants, or locally delivered EET-scavenging nanoplatforms. Microbial clearance, infection recurrence, mucosal toxicity, nanoparticle retention, systemic exposure, and preservation of beneficial eosinophil functions should be included as predefined safety outcomes because extracellular traps may contribute to antibacterial and antifungal defense. Overall, EET-directed treatment remains an experimental precision-medicine concept, with the strongest clinical evidence showing that rhDNase can reduce extracellular DNA without consistently improving clinical outcomes and the strongest preclinical evidence supporting simultaneous targeting of extracellular DNA, mucin cross-linking, ROS generation, histone citrullination, and persistent eosinophil-derived extracellular material.

## 8. Conclusions

Eosinophil extracellular traps represent an important extension of eosinophil effector biology, linking host defense, barrier immunity, and tissue pathology. EETs may support protective responses by immobilizing pathogens and concentrating eosinophil granule proteins at sites where classical phagocytosis is inefficient, particularly at mucosal and epithelial barriers. However, when trap formation becomes excessive, persistent, or insufficiently cleared, the same extracellular DNA, histones, granule proteins, and cell-free granules may contribute to epithelial injury, mucus viscosity, thromboinflammation, fibroinflammatory remodeling, and chronic eosinophilic inflammation. Current evidence supports a dual role for EETs across infectious, allergic, airway, otologic, systemic, cutaneous, gastrointestinal, and malignant diseases, but their biological meaning depends strongly on tissue context, disease stage, local stimuli, and the balance between trap formation and clearance. Future studies should move beyond descriptive detection of extracellular DNA and define standardized criteria for EET identification, including markers that distinguish eosinophil traps from neutrophil traps and nuclear from mitochondrial EETosis. Larger human tissue studies, longitudinal clinical cohorts, and functional experiments are also needed to determine whether EETs are reliable biomarkers, active drivers of disease, or therapeutic targets. Clarifying these issues will be essential for understanding when EETs contribute to protection and when they become mediators of persistent inflammation and tissue damage.

## Figures and Tables

**Table 1:** Biological and clinical relevance of eosinophils and eosinophil granule release mechanisms.

Aspect of Eosinophil Biology	Main Biological Concept	Relevance in Health and Disease	Clinical Significance/Therapeutic Implication	Key Take-Home Message	References
Development and survival	Eosinophils develop in the bone marrow under the influence of IL-3, IL-5, and GM-CSF, with IL-5 playing a central role in their maturation and persistence.	These cytokine signals determine how many eosinophils enter the circulation and how strongly they respond once they reach inflamed tissues.	Targeting IL-5 or its receptor can reduce eosinophil survival and tissue accumulation in eosinophil-driven diseases.	Eosinophils are already biologically primed before they arrive at sites of inflammation.	[3,4,5,6]
Tissue distribution	Although eosinophils are few in the blood, they are strategically positioned in several tissues, especially at mucosal and barrier surfaces.	Their tissue localization allows them to participate in host defense, immune regulation, repair, and maintenance of local homeostasis.	Treatment approaches should consider that eosinophils may have protective roles in some tissues, not only pathogenic effects.	Eosinophils are best understood as tissue-active immune cells rather than simple circulating granulocytes.	[7,8,9,10,11,12,13,14,15,16,17]
Granule biology	Eosinophil granules contain powerful mediators such as MBP, ECP, EDN, and EPX/EPO, together with cytokines, chemokines, enzymes, and growth factors.	These mediators can help control pathogens, but when released excessively, they may damage epithelium and promote chronic inflammation.	Granule proteins may be useful as markers of eosinophil activation and as potential targets for limiting tissue injury.	The same granule machinery that supports host defense can also drive disease.	[20,22,23,24,25,26,27,28]
Pathological activation	Eosinophils become harmful when their recruitment, survival, or activation persists beyond the needs of tissue protection.	In allergic and chronic inflammatory diseases, they contribute to epithelial injury, mucus production, neuroimmune activation, remodeling, and fibrosis.	Therapies that reduce eosinophil activation or mediator release may help prevent long-term structural tissue damage.	Eosinophils become pathogenic when protective responses are amplified or fail to resolve.	[18,19,20,21,29,30,31,32,33]
Granule release mechanisms	Eosinophils release granule mediators through several pathways, including classical exocytosis, compound exocytosis, piecemeal degranulation, and cytolysis.	Some pathways allow controlled secretion by viable cells, whereas cytolysis releases intact extracellular granules that may remain active after cell disruption.	Recognizing the type of granule release may improve interpretation of tissue findings and help distinguish controlled secretion from destructive inflammation.	Extracellular granule proteins do not always reflect the same biological event.	[2,14,25,34,36,37,38,39,40,41,42,43,44]

**Table 2:** EET-inducing stimuli, experimental conditions, molecular requirements, and principal readouts.

EET-Inducing Stimulus	Species/Eosinophil Source	Priming Conditions	DNA Origin	ROS Dependence	PAD4 Dependence	Key Readouts and Principal Findings	References
LPS	Human peripheral-blood eosinophils	IL-5 or IFN-γ priming before stimulation	Mitochondrial	ROS-dependent; associated with NADPH oxidase activity	Not evaluated	Rapid extracellular mtDNA release with initially preserved plasma membrane integrity; extracellular DNA associated with eosinophil granule proteins; antibacterial trapping activity	[59,62]
C5a	Human peripheral-blood eosinophils	IL-5 or IFN-γ priming	Mitochondrial	ROS-dependent	Not evaluated	Rapid mtDNA extrusion; viable eosinophils retained nuclear morphology during the early phase; extracellular DNA nets contributed to bacterial immobilization	[59]
CCL11/eotaxin-1	Human peripheral-blood eosinophils	IL-5 or IFN-γ priming	Mitochondrial	ROS-dependent	Not evaluated	Rapid non-cytolytic mtDNA release and formation of extracellular DNA networks containing eosinophil granule proteins	[59]
Immobilized IgG	Human peripheral-blood eosinophils	No obligatory cytokine priming clearly established in the cited study	Predominantly nuclear	ROS/NADPH oxidase-dependent in the experimental settings examined	Not established	Nuclear delobulation, chromatin decondensation, plasma membrane rupture, extracellular chromatin fibers, and release of intact secretion-competent eosinophil granules	[34,61,62]
Immobilized IgA	Human peripheral-blood eosinophils	No obligatory priming clearly established	Predominantly nuclear	ROS/NADPH oxidase-dependent	Not established	Cytolytic EETosis with extracellular DNA, histones, eosinophil granule proteins, and free extracellular granules; particularly relevant to mucosal antibody-mediated activation	[34,61,62]
PAF with IL-5 or GM-CSF	Human peripheral-blood eosinophils	IL-5 or GM-CSF priming	Nuclear	Generally ROS-dependent	Not established	Chromatin decondensation, nuclear-envelope disruption, membrane rupture, and extracellular DNA trap formation	[34,40,61,62]
PMA	Human peripheral-blood eosinophils	Usually no additional priming required; protocols vary	Nuclear	ROS/NADPH oxidase-dependent	Variable or incompletely defined	Extensive chromatin decondensation and cytolytic extracellular DNA release; commonly used as a strong pharmacological positive control	[34,54,61,62]
Calcium ionophore A23187	Human peripheral-blood eosinophils	Usually no cytokine priming required	Nuclear	May proceed through calcium-dominant signaling; ROS requirement varies by protocol	Possible, but not consistently demonstrated	Increased intracellular Ca^2+^, chromatin remodeling, histone modification, and extracellular DNA release	[40,61,62]
*Staphylococcus aureus*	Human airway tissue and peripheral-blood eosinophils	Tissue inflammatory environment; cytokine priming may facilitate the response	Mitochondrial and/or nuclear origin not uniformly distinguished in tissue studies	Not conclusively defined	Not evaluated	Extracellular eosinophilic DNA structures colocalized with eosinophil proteins and were found adjacent to bacteria at epithelial barrier defects; bacterial entrapment was observed	[59,64]
*Aspergillus fumigatus* conidia	Human peripheral-blood eosinophils; bronchial mucus from patients with ABPA	No obligatory exogenous cytokine priming in the principal in vitro studies	Predominantly nuclear	ROS- and NOX-independent	PAD4-independent	Cytolytic EETosis, LDH release, loss of membrane integrity, extracellular DNA colocalization with histones, fungal-conidium entrapment, and increased mucus viscosity; signaling involved Mac-1/CD11b, Syk, SFKs, Akt, Ca^2+^, and p38 MAPK	[11,65,66,67,77]
Respiratory syncytial virus	Murine eosinophilic asthma model	OVA sensitization and airway challenge created an eosinophilic inflammatory environment	Not directly established	Not directly established in the cited RSV study	Not evaluated	Increased eosinophil extracellular DNA structures in the lungs, accompanied by enhanced eosinophilic airway inflammation and mucus production	[78]
OVA-induced allergic airway inflammation	Mouse asthma model	OVA sensitization and challenge	Predominantly nuclear, based on cytolytic morphology; molecular origin not always directly verified	ROS-dependent; reduced by DPI and NAC	Not established	Increased extracellular DNA structures, airway eosinophilia, inflammatory-cell recruitment, mucus production, and tissue inflammation; antioxidant or NOX inhibition reduced EET formation	[79]
TSLP	Human peripheral-blood eosinophils and eosinophils in allergic skin disease	Direct stimulation through the TSLPR/IL-7Rα receptor complex; no separate priming required	Mitochondrial	ROS-dependent	Not evaluated	Rapid mtDNA release, extracellular DNA networks containing eosinophil granule proteins, and increased EET formation in allergic inflammatory conditions	[80]
MSU crystals	Human peripheral-blood eosinophils	No cytokine priming required in the reported experimental system	Nuclear	Not clearly established	Not established	Extracellular DNA trap formation after crystal exposure, with chromatin-based networks surrounding MSU aggregates; response also observed in neutrophils and basophils	[81]
Long-chain unsaturated fatty acids	Human eosinophils and experimental airway-inflammation models	Metabolic exposure within an eosinophilic airway environment; no conventional cytokine priming required	Nuclear	ROS involvement reported, but the precise requirement depends on the fatty acid and experimental condition	Not fully established	Filamentous extracellular DNA release, eosinophil cytolysis, airway inflammation, and association with reduced corticosteroid responsiveness	[82]
LysoPS	Human peripheral-blood eosinophils, including cells from patients with severe asthma	No conventional cytokine priming required	Nuclear	ROS-independent	PAD4-dependent	Extensive extracellular DNA release, histone citrullination, cytolysis, degranulation, and increased responses in severe-asthma eosinophils; signaling mediated through P2Y10	[83]
Autoantibodies and immune complexes	Human peripheral-blood eosinophils and eosinophil-rich inflammatory tissues	Dependent on Fc-receptor engagement; priming conditions vary	Nuclear	Generally linked to ROS generation, but stimulus-specific evidence is limited	Not established	Cytolytic morphology, extracellular chromatin, eosinophil granule deposition, and free extracellular granules in autoimmune and eosinophilic inflammatory lesions	[34,40,61,62]

**Table 3:** Protective and pathogenic implications of eosinophil extracellular traps in infectious settings.

Infectious Context	How EETs Are Involved	Why This Matters Biologically	Clinical Significance/Therapeutic Implication	Key Take-Home Message	References
Bacterial infections	Cytokine-primed eosinophils may release extracellular DNA networks after contact with bacteria, especially when bacteria are extracellular, opsonized, or attached to damaged epithelial surfaces.	EETs may help keep bacteria locally contained and expose them to concentrated eosinophil granule mediators, but their effect is difficult to separate from degranulation, ROS, complement, antibodies, and other innate immune responses.	Bacterial EETosis may support mucosal defense, but it should not be interpreted as an isolated or universally dominant antibacterial mechanism.	EETs probably help increase local antimicrobial pressure rather than acting as a stand-alone bacterial killing system.	[34,54,59,64,70,87,94]
*Staphylococcus aureus* infection	*S. aureus* can strongly induce EET formation in eosinophil-rich airway disease, particularly when bacteria reach damaged epithelial areas.	Local EET formation may help trap *S. aureus* at epithelial defects, but persistent DNA-rich traps may also increase mucus viscosity, epithelial injury, and chronic inflammation.	In chronic rhinosinusitis with nasal polyps, EETs may be both protective and harmful, depending on whether the response remains localized or becomes persistent.	*S. aureus*-induced EETosis shows how the same response can support barrier defense and also maintain tissue inflammation.	[59,60,64,67,95,96,97,98]
*Staphylococcus epidermidis*-related barrier responses	*S. epidermidis* is usually a commensal organism, but after barrier disruption it may activate eosinophils together with epithelial-derived signals such as TSLP.	This response may help protect injured skin or mucosa from bacterial spread, although excessive eosinophil activity could disturb the balance of the local microbiome.	In type 2 skin inflammation, eosinophil responses to commensal bacteria should be viewed carefully because protection and dysbiosis may overlap.	EET-related responses to S. epidermidis may be useful after barrier injury, but only when they remain well controlled.	[80,99,100]
*Escherichia coli* infection	Activated eosinophils can respond to opsonized *E. coli* by releasing extracellular DNA that contributes to antibacterial activity.	The marked reduction in antibacterial activity after DNase treatment suggests that extracellular DNA has a functional role, not merely a bystander presence.	These findings support the relevance of EETs in antibacterial defense, although their in vivo importance probably depends on tissue context and cooperation with other immune mechanisms.	Against *E. coli*, EETs may contribute to defense through DNA-dependent extracellular activity rather than classical phagocytosis.	[58,59,101]
Parasitic infections	Helminths and larval forms are often too large for conventional phagocytosis, making extracellular eosinophil responses biologically plausible.	EETs may immobilize parasite forms and concentrate toxic eosinophil mediators near their surface, but direct parasite killing remains less certain.	Parasite-induced EETosis may be part of antihelminth immunity, although stronger in vivo evidence is still needed.	In parasitic infection, EETs are best viewed as a way to restrain large extracellular targets and shape local inflammation.	[91,92,102,103]
Fungal infections and *Aspergillus*-related disease	Fungal structures may activate eosinophils through cell-wall recognition pathways, and EETs can be detected in eosinophil-rich fungal airway disease.	EETs may bind and immobilize fungal material, especially within thick mucus, but current evidence is stronger for containment and inflammatory amplification than for direct fungal killing.	In ABPA and related airway disorders, EET-rich mucus may help retain fungal material, but it may also worsen obstruction, epithelial irritation, and chronic eosinophilic inflammation.	In fungal disease, EETs may contain fungal elements, but persistent trap formation can also become part of the pathology.	[54,65,66,77,90,104,105,106,107,108]

**Table 4:** Pathological significance of eosinophil extracellular traps across eosinophil-associated diseases.

Disease Setting	Pathogenic EET-Related Pattern	Main Tissue Consequence	Clinical Significance/Therapeutic Implication	Key Take-Home Message	References
Eosinophilic chronic rhinosinusitis with nasal polyps	In eosinophilic CRS, activated tissue eosinophils release extracellular DNA, granule proteins, and galectin-10-rich material into sinonasal mucosa and secretions.	This material can thicken mucus, impair clearance, damage epithelium, favor bacterial retention, and contribute to refractory polypoid inflammation.	EETs may help identify patients with more severe, recurrent, mucus-dominant, and treatment-resistant disease, in whom conventional anti-inflammatory therapy may be insufficient.	In eosinophilic CRS, EETs help turn eosinophil activation into persistent mucus obstruction and chronic mucosal injury.	[54,64,67,82,113,114,120,121,122,123,124,125,126]
Otitis media with effusion	Middle-ear effusions may contain extracellular DNA, citrullinated histone H3–DNA complexes, and eosinophil-associated ECP–DNA complexes.	DNA–protein networks may increase effusion viscosity, retain inflammatory mediators, and sustain mucosal irritation through danger-sensing pathways.	Targeting extracellular DNA or trap-associated inflammation could become relevant for persistent, viscous, or refractory effusions, although clinical validation is still needed.	In OME, extracellular traps may help explain why some effusions remain thick, inflammatory, and difficult to clear.	[54,128,129,130,131]
Severe eosinophilic asthma	Airway eosinophils may release EETs under the influence of IL-5, IL-13, epithelial alarmins, oxidative stress, and microbial or inflammatory signals.	EETs can increase mucus viscosity, injure epithelium, impair mucociliary clearance, and reinforce type 2 inflammation through epithelial–eosinophil feedback loops.	EETs are promising but still unvalidated biomarkers; mechanistically, they may help connect eosinophil activation with mucus plugging, airflow limitation, and severe disease activity.	In severe asthma, EETs may bridge eosinophilic inflammation, epithelial injury, and mucus-driven airway obstruction.	[20,54,69,76,79,85,87,135,136,137,138,139,140,141,142,143]
Eosinophilic COPD and chronic eosinophilic pneumonia	In eosinophil-rich pulmonary disease, cytolytic EETosis may deposit chromatin-rich material, extracellular DNA, and granule proteins into airway secretions or alveolar spaces.	These structures may increase the total extracellular DNA burden, worsen mucus obstruction, and potentially interact with neutrophil extracellular trap pathways.	Recognizing EETosis may help define eosinophil-dominant pulmonary phenotypes, although evidence is stronger in eosinophilic COPD than in chronic eosinophilic pneumonia.	In selected pulmonary disorders, EETs may contribute to obstruction and inflammation by adding eosinophil-derived DNA and cytotoxic material to the airway lumen.	[58,132,133,147,148]
EGPA and hypereosinophilic cardiac disease	Systemic eosinophil activation may drive cytolytic EETosis with extracellular chromatin, CitH3-positive traps, cell-free granules, and galectin-10-related deposits.	EET-derived material may amplify endothelial injury, granulomatous inflammation, thromboinflammation, fibroblast activation, myocardial remodeling, and organ dysfunction.	Anti-IL-5 therapy, EET-related biomarkers, and imaging markers of fibroblast activation may help link eosinophil activation with vascular or cardiac tissue damage.	In systemic eosinophilic disease, EETs may connect eosinophil cytolysis with vascular injury, thrombosis, and fibrosis.	[34,40,72,86,149,150,151,152,153,154,155,156,157,158,159,160]
Inflammatory skin and ocular surface diseases	EETs may appear in acute or exacerbated eosinophil-rich skin lesions, autoimmune blistering disease, Wells syndrome, and allergic ocular surface inflammation.	Extracellular DNA and granule proteins may contribute to epidermal or ocular barrier injury, flame figure formation, blister inflammation, and local cytokine amplification.	EETs may help explain severe or acute eosinophil-rich tissue injury, but better eosinophil-specific markers are needed to separate true EETosis from nonspecific eosinophil breakdown.	In skin and ocular disease, EETs are most relevant when eosinophil activation becomes intense enough to leave persistent extracellular inflammatory deposits.	[58,109,165,166,167,168,169,170,171,172,173,174,175,176,177,178,179,180,181,182,183,184]
Gastrointestinal mucosal inflammation	In EoE and Crohn’s disease, EETs may form where barrier dysfunction exposes eosinophils to microbial products, epithelial stress signals, and type 2 cytokines.	Persistent extracellular DNA and granule proteins may retain inflammatory material, aggravate epithelial irritation, and sustain chronic mucosal immune activation.	EETs may represent a link between epithelial barrier failure and eosinophil-driven mucosal pathology, but their causal role in disease progression remains incompletely defined.	In gastrointestinal inflammation, EETs should be seen as part of a wider barrier-stress response rather than as an isolated eosinophil event.	[21,58,59,80,110,185,186,187,188,189,190,191]
Sterile vascular and IgG4-related fibroinflammatory disease	EETs may deposit DNA, chromatin, MBP-positive material, cell-free granules, galectin-10, and Charcot–Leyden crystal-related deposits in vascular or fibroinflammatory lesions.	These deposits may promote endothelial activation, platelet interaction, thromboinflammation, immune persistence, and tissue remodeling.	EETosis is a plausible pathogenic link, but its independent causal contribution in atherosclerosis and IgG4-related disease still requires tissue-based validation.	In sterile inflammatory disease, EETs may act as durable eosinophil-derived material that sustains inflammation and remodeling after eosinophil activation.	[34,157,192,193,194,195,196,197,198,199,200,201,202,203]

**Table 5:** Antitumor and tumor-supportive roles of eosinophil extracellular traps in cancer.

Tumor Context	How EETs May Contribute	Main Pathological Consequence	Clinical Significance/Therapeutic Implication	Key Take-Home Message
General tumor microenvironment	Tumors may recruit eosinophils through cytokines and chemokines such as IL-33, HMGB1, CCL11, CCL24, CCL5, and CCL3. Once present, eosinophils may release granule proteins, cytokines, lipid mediators, and possibly EETs.	EETosis may create an additional pathway of communication between eosinophils, malignant cells, stromal cells, and other immune populations within the tumor microenvironment.	Eosinophil-rich tumors should be interpreted carefully, because eosinophils may support antitumor immunity in some settings but contribute to tumor-supportive inflammation in others.	Eosinophils in cancer are biologically flexible, and their meaning depends strongly on the local tumor environment.
Dual antitumor and tumor-promoting potential	EETs may concentrate cytotoxic eosinophil proteins close to malignant cells, but they may also support chronic inflammation, matrix remodeling, angiogenesis, immune suppression, and tumor-cell migration.	The same extracellular DNA–protein network may damage tumor cells in one context while helping tumor progression in another.	Therapies targeting eosinophils or trap-related inflammation will need to consider tumor type, immune context, and whether eosinophils are acting protectively or pathologically.	EETs should not be described as simply good or bad in cancer; their effect is likely tumor-specific.
Pan-cancer transcriptomic evidence	EET-related gene signatures are higher in some tumors with poorer outcomes and are linked with immune suppression, stromal remodeling, checkpoint expression, and reduced cytotoxic immune-cell abundance.	These signatures may reflect eosinophil activation or broader inflammatory remodeling, but they do not prove that organized EETs are present in tumor tissue.	EET-related scores may become useful only after validation by tissue studies showing extracellular DNA together with eosinophil granule proteins in the same tumor areas.	Transcriptomic signatures are useful clues, but they remain indirect evidence of EET biology.
Head and neck squamous cell carcinoma	In HNSCC, eosinophil-related signals and EET-associated molecular patterns may overlap with epithelial barrier injury, SPINK5/LEKTI changes, protease imbalance, and stromal remodeling.	This may reflect a tumor environment in which mucosal injury, eosinophil activation, epithelial remodeling, and invasion are biologically connected.	Direct tissue validation is still needed before EETosis can be considered a confirmed mechanism in HNSCC progression or immune regulation.	In HNSCC, current data suggest a possible link between eosinophil activity and tumor remodeling, but not yet definitive EET formation.
Endometrial cancer	EET-related regulatory genes are altered in endometrial cancer, and S100A9 appears to be associated with inflammatory signaling, immune infiltration, progression, and poorer outcome.	These findings may indicate a broader tumor-promoting inflammatory program rather than direct extracellular trap formation by eosinophils.	S100A9 and related inflammatory markers may help identify aggressive immune-remodeling patterns, but they should not be treated as specific markers of EETosis.	In endometrial cancer, EET-related biology currently points more to inflammatory remodeling than to proven EET formation.
Gastric cancer	Limited case-level evidence has shown tumor-associated eosinophils at different stages compatible with EETosis, including chromatin decondensation, membrane disruption, granule release, and extracellular trap formation.	Local EETosis may occur within the gastric tumor microenvironment, although its biological role remains uncertain.	Larger tissue studies using eosinophil-specific EET markers are needed to determine whether EETosis contributes to antitumor activity, tumor progression, or simply reflects intense eosinophil activation.	Gastric cancer provides one of the clearest morphological examples of possible EETosis, but its functional meaning remains unresolved.
Classic Hodgkin lymphoma	In eosinophil-rich classic Hodgkin lymphoma, extracellular trap-like structures have been described in areas with dense eosinophil infiltration.	These structures may reflect local eosinophil activation and deposition of extracellular DNA and eosinophil-derived products within the inflammatory tumor stroma.	Because evidence is mainly case-based and histological, EET-like material should be interpreted as suggestive, not as proof of an active tumor-modifying EET pathway.	In classic Hodgkin lymphoma, EET-like structures may mark intense eosinophil-rich inflammation, but their impact on tumor behavior remains uncertain.

## Data Availability

No new data were created or analyzed in this study. Data sharing is not applicable to this article.

## Abbreviations

The following abbreviations are used in this manuscript:

A23187	Calcium ionophore A23187
AAV	ANCA-associated vasculitis
ABPA	Allergic bronchopulmonary aspergillosis
AKC	Atopic keratoconjunctivitis
Akt/PKB	Akt/protein kinase B
ALI	Air–liquid interface
ANCA	Antineutrophil cytoplasmic antibody
BALF	Bronchoalveolar lavage fluid
BDNF	Brain-derived neurotrophic factor
BP	Bullous pemphigoid
BP180	Bullous pemphigoid antigen 180
BP230	Bullous pemphigoid antigen 230
C5a	Complement component 5a
Ca^2+^	Calcium
CCDC25	Coiled-coil domain-containing protein 25
CCL3	C-C motif chemokine ligand 3
CCL5	C-C motif chemokine ligand 5
CCL11	C-C motif chemokine ligand 11/eotaxin-1
CCL24	C-C motif chemokine ligand 24/eotaxin-2
CCL26	C-C motif chemokine ligand 26/eotaxin-3
CD8	Cluster of differentiation 8
CD11b	Cluster of differentiation 11b
CD18	Cluster of differentiation 18
CD69	Cluster of differentiation 69
cHL	Classic Hodgkin lymphoma
CitH3	Citrullinated histone H3
CK18	Cytokeratin 18
COPD	Chronic obstructive pulmonary disease
CRS	Chronic rhinosinusitis
CRSsNP	Chronic rhinosinusitis without nasal polyps
CRSwNP	Chronic rhinosinusitis with nasal polyps
CT	Computed tomography
DNA	Deoxyribonucleic acid
DNase I	Deoxyribonuclease I
DPI	Diphenyleneiodonium
dsDNA	Double-stranded DNA
ECM	Extracellular matrix
ECP	Eosinophil cationic protein
eCRS	Eosinophilic chronic rhinosinusitis
EDN	Eosinophil-derived neurotoxin
eDNA	Extracellular DNA
EETosis	Eosinophil extracellular trap formation
EETs	Eosinophil extracellular traps
EGPA	Eosinophilic granulomatosis with polyangiitis
EoE	Eosinophilic esophagitis
EoSVs	Eosinophil sombrero vesicles
EPO	Eosinophil peroxidase
EPX	Eosinophil peroxidase
ETosis	Extracellular trap formation
FAPI	Fibroblast activation protein inhibitor
Fc	Fragment crystallizable region
FEV1	Forced expiratory volume in 1 s
GI	Gastrointestinal
GM-CSF	Granulocyte–macrophage colony-stimulating factor
HER2	Human epidermal growth factor receptor 2
HES	Hypereosinophilic syndrome
HMGB1	High-mobility group box 1
HNSCC	Head and neck squamous cell carcinoma
ICAM-1/CD54	Intercellular adhesion molecule-1/cluster of differentiation 54
IFN-γ	Interferon-γ
IgA	Immunoglobulin A
IgG	Immunoglobulin G
IgG4	Immunoglobulin G4
IL	Interleukin
IL-1β	Interleukin-1β
IL-2	Interleukin-2
IL-3	Interleukin-3
IL-4	Interleukin-4
IL-5	Interleukin-5
IL-6	Interleukin-6
IL-7Rα	Interleukin-7 receptor alpha
IL-8	Interleukin-8
IL-13	Interleukin-13
IL-17	Interleukin-17
IL-25	Interleukin-25
IL-31	Interleukin-31
IL-32	Interleukin-32
IL-33	Interleukin-33
ILC2s	Group 2 innate lymphoid cells
IRE1α	Inositol-requiring enzyme 1α
JAK	Janus kinase
LDH	Lactate dehydrogenase
LEKTI	Lymphoepithelial Kazal-type-related inhibitor
LL-37	Cathelicidin LL-37
LPS	Lipopolysaccharide
LysoPS	Lysophosphatidylserine
Mac-1	Macrophage-1 antigen
MBP	Major basic protein
MSU	Monosodium urate
mtDNA	Mitochondrial DNA
NAC	N-acetylcysteine
NADPH	Nicotinamide adenine dinucleotide phosphate
NETosis	Neutrophil extracellular trap formation
NETs	Neutrophil extracellular traps
NF-κB	Nuclear factor kappa B
NGF	Nerve growth factor
NOX	NADPH oxidase
OME	Otitis media with effusion
OVA	Ovalbumin
P2Y10	P2Y purinoceptor 10
p38 MAPK	p38 mitogen-activated protein kinase
PAD4	Peptidyl arginine deiminase 4
PAF	Platelet-activating factor
PET/CT	Positron emission tomography/computed tomography
PMA	Phorbol 12-myristate 13-acetate
RNASE2	Ribonuclease A family member 2
RNASE3	Ribonuclease A family member 3
ROS	Reactive oxygen species
SFKs	Src-family kinases
SPINK5	Serine peptidase inhibitor Kazal type 5
STAT3	Signal transducer and activator of transcription 3
STAT5	Signal transducer and activator of transcription 5
Syk	Spleen tyrosine kinase
TGF-β	Transforming growth factor-β
Th2	T helper 2 cell
TLR9	Toll-like receptor 9
TLRs	Toll-like receptors
TME	Tumor microenvironment
TNF-α	Tumor necrosis factor-α
Treg	Regulatory T-cell
TSLP	Thymic stromal lymphopoietin
TSLPR	Thymic stromal lymphopoietin receptor
VEGF	Vascular endothelial growth factor
VKC	Vernal keratoconjunctivitis
XBP1s	Spliced X-box binding protein 1

## References

[1] Klion A.D., Ackerman S.J., Bochner B.S. (2020). Contributions of Eosinophils to Human Health and Disease. Annu. Rev. Pathol. Mech. Dis..

[2] Sanchez Santos A., Socorro Avila I., Galvan Fernandez H., Cazorla Rivero S., Lemes Castellano A., Cabrera Lopez C. (2024). Eosinophils: Old cells, new directions. Front. Med..

[3] Rodrigo-Muñoz J.M., Gil-Martínez M., Sastre B., Del Pozo V. (2021). Emerging Evidence for Pleiotropism of Eosinophils. Int. J. Mol. Sci..

[4] Jorssen J., Van Hulst G., Mollers K., Pujol J., Petrellis G., Baptista A.P., Schetters S., Baron F., Caers J., Lambrecht B.N. (2024). Single-cell proteomics and transcriptomics capture eosinophil development and identify the role of IL-5 in their lineage transit amplification. Immunity.

[5] Whetstone C.E., Amer R., Maqbool S., Javed T., Gauvreau G.M. (2025). Pathobiology and Regulation of Eosinophils, Mast Cells, and Basophils in Allergic Asthma. Immunol. Rev..

[6] Fulkerson P.C., Schollaert K.L., Bouffi C., Rothenberg M.E. (2014). IL-5 triggers a cooperative cytokine network that promotes eosinophil precursor maturation. J. Immunol..

[7] Gurtner A., Crepaz D., Arnold I.C. (2023). Emerging functions of tissue-resident eosinophils. J. Exp. Med..

[8] Gurtner A., Borrelli C., Gonzalez-Perez I., Bach K., Acar I.E., Núñez N.G., Crepaz D., Handler K., Vu V.P., Lafzi A. (2023). Active eosinophils regulate host defence and immune responses in colitis. Nature.

[9] Gazzinelli-Guimaraes P.H., Jones S.M., Voehringer D., Mayer-Barber K.D., Samarasinghe A.E. (2024). Eosinophils as modulators of host defense during parasitic, fungal, bacterial, and viral infections. J. Leukoc. Biol..

[10] Siddiqui S., Bachert C., Bjermer L., Buchheit K.M., Castro M., Qin Y., Rupani H., Sagara H., Howarth P., Taillé C. (2023). Eosinophils and tissue remodeling: Relevance to airway disease. J. Allergy Clin. Immunol..

[11] Arias-González L., Rodríguez-Alcolado L., Laserna-Mendieta E.J., Navarro P., Lucendo A.J., Grueso-Navarro E. (2024). Fibrous Remodeling in Eosinophilic Esophagitis: Clinical Facts and Pathophysiological Uncertainties. Int. J. Mol. Sci..

[12] Gurtner A., Gonzalez-Perez I., Arnold I.C. (2021). Intestinal eosinophils, homeostasis and response to bacterial intrusion. Semin. Immunopathol..

[13] Ruzic A., Trus M., Sehmi R., Mukherjee M. (2026). Understanding Eosinophil Heterogeneity: The Known and Unknown. Cells.

[14] Erjefält J.S. (2025). Spatial Eosinophil Phenotypes as Immunopathogenic Determinants in Inflammatory Diseases. Cells.

[15] Noble S.L., Mules T.C., Le Gros G., Inns S. (2024). The immunoregulatory potential of eosinophil subsets. Immunol. Cell Biol..

[16] Arnold I.C., Munitz A. (2024). Spatial adaptation of eosinophils and their emerging roles in homeostasis, infection and disease. Nat. Rev. Immunol..

[17] Brüggemann T.R., Peh H.Y., Tavares L.P., Nijmeh J., Shay A.E., Rezende R.M., Lanser T.B., Serhan C.N., Levy B.D. (2023). Eosinophil Phenotypes Are Functionally Regulated by Resolvin D2 during Allergic Lung Inflammation. Am. J. Respir. Cell Mol. Biol..

[18] Lombardi C., Berti A., Cottini M. (2022). The emerging roles of eosinophils: Implications for the targeted treatment of eosinophilic-associated inflammatory conditions. Curr. Res. Immunol..

[19] Mormile M., Mormile I., Fuschillo S., Rossi F.W., Lamagna L., Ambrosino P., de Paulis A., Maniscalco M. (2023). Eosinophilic Airway Diseases: From Pathophysiological Mechanisms to Clinical Practice. Int. J. Mol. Sci..

[20] Acharya K.R., Ackerman S.J. (2014). Eosinophil granule proteins: Form and function. J. Biol. Chem..

[21] Tomii T., Kano G. (2025). Eosinophils in inflammatory bowel disease pathogenesis: An ROS-centric view. Front. Allergy.

[22] Melo R.C.N., Rothenberg M.E. (2025). Imaging eosinophil secretory granules: From storage containers to active, immune responder organelles. J. Allergy Clin. Immunol..

[23] Miyabe Y., Kobayashi Y., Fukuchi M., Saga A., Moritoki Y., Saga T., Akuthota P., Ueki S. (2021). Eosinophil-mediated inflammation in the absence of eosinophilia. Asia Pac. Allergy.

[24] Davoine F., Lacy P. (2014). Eosinophil cytokines, chemokines, and growth factors: Emerging roles in immunity. Front. Immunol..

[25] Fettrelet T., Gigon L., Karaulov A., Yousefi S., Simon H.U. (2021). The Enigma of Eosinophil Degranulation. Int. J. Mol. Sci..

[26] Melo R.C.N., Weller P.F. (2018). Contemporary understanding of the secretory granules in human eosinophils. J. Leukoc. Biol..

[27] Soragni A., Yousefi S., Stoeckle C., Soriaga A.B., Sawaya M.R., Kozlowski E., Schmid I., Radonjic-Hoesli S., Boutet S., Williams G.J. (2015). Toxicity of eosinophil MBP is repressed by intracellular crystallization and promoted by extracellular aggregation. Mol. Cell.

[28] Yang D., Rosenberg H.F., Chen Q., Dyer K.D., Kurosaka K., Oppenheim J.J. (2003). Eosinophil-derived neurotoxin (EDN), an antimicrobial protein with chemotactic activities for dendritic cells. Blood.

[29] Kim C.K., Callaway Z., Pawankar R. (2023). Eosinophil granule proteins as a biomarker in managing asthma and allergies. Asia Pac. Allergy.

[30] Peen E., Hahn P., Lauwers G., Williams R.C., Gleich G., Kephart G.M. (2000). Churg-Strauss syndrome: Localization of eosinophil major basic protein in damaged tissues. Arthritis Rheum..

[31] Guilpain P., Auclair J.F., Tamby M.C., Servettaz A., Mahr A., Weill B., Guillevin L., Mouthon L. (2007). Serum eosinophil cationic protein: A marker of disease activity in Churg-Strauss syndrome. Ann. N. Y. Acad. Sci..

[32] Furuta G.T., Nieuwenhuis E.E., Karhausen J., Gleich G., Blumberg R.S., Lee J.J., Ackerman S.J. (2005). Eosinophils alter colonic epithelial barrier function: Role for major basic protein. Am. J. Physiol. Gastrointest. Liver Physiol..

[33] Adamko D.J., Wu Y., Ajamian F., Ilarraza R., Moqbel R., Gleich G.J. (2008). The effect of cationic charge on release of eosinophil mediators. J. Allergy Clin. Immunol..

[34] Ueki S., Melo R.C., Ghiran I., Spencer L.A., Dvorak A.M., Weller P.F. (2013). Eosinophil extracellular DNA trap cell death mediates lytic release of free secretion-competent eosinophil granules in humans. Blood.

[35] Novosad J., Krčmová I., Souček O., Drahošová M., Sedlák V., Kulířová M., Králíčková P. (2023). Subsets of Eosinophils in Asthma, a Challenge for Precise Treatment. Int. J. Mol. Sci..

[36] Spencer L.A., Bonjour K., Melo R.C., Weller P.F. (2014). Eosinophil secretion of granule-derived cytokines. Front. Immunol..

[37] Melo R.C.N., Weller P.F. (2010). Piecemeal degranulation in human eosinophils: A distinct secretion mechanism underlying inflammatory responses. Histol. Histopathol..

[38] Melo R.C., Perez S.A., Spencer L.A., Dvorak A.M., Weller P.F. (2005). Intragranular vesiculotubular compartments are involved in piecemeal degranulation by activated human eosinophils. Traffic.

[39] Neves V.H., Palazzi C., Malta K.K., Bonjour K., Kneip F., Dias F.F., Neves J.S., Weller P.F., Melo R.C.N. (2024). Extracellular sombrero vesicles are hallmarks of eosinophilic cytolytic degranulation in tissue sites of human diseases. J. Leukoc. Biol..

[40] Neves V.H., Palazzi C., Bonjour K., Ueki S., Weller P.F., Melo R.C.N. (2022). In Vivo ETosis of Human Eosinophils: The Ultrastructural Signature Captured by TEM in Eosinophilic Diseases. Front. Immunol..

[41] Neves J.S., Perez S.A., Spencer L.A., Melo R.C., Reynolds L., Ghiran I., Mahmudi-Azer S., Odemuyiwa S.O., Dvorak A.M., Moqbel R. (2008). Eosinophil granules function extracellularly as receptor-mediated secretory organelles. Proc. Natl. Acad. Sci. USA.

[42] Erjefält J.S., Andersson M., Greiff L., Korsgren M., Gizycki M., Jeffery P.K., Persson G.A. (1998). Cytolysis and piecemeal degranulation as distinct modes of activation of airway mucosal eosinophils. J. Allergy Clin. Immunol..

[43] Uller L., Andersson M., Greiff L., Persson C.G., Erjefält J.S. (2004). Occurrence of apoptosis, secondary necrosis, and cytolysis in eosinophilic nasal polyps. Am. J. Respir. Crit. Care Med..

[44] Saffari H., Hoffman L.H., Peterson K.A., Fang J.C., Leiferman K.M., Pease L.F., Gleich G.J. (2014). Electron microscopy elucidates eosinophil degranulation patterns in patients with eosinophilic esophagitis. J. Allergy Clin. Immunol..

[45] Tonello S., Vercellino N., D’Onghia D., Fracchia A., Caria G., Sola D., Tillio P.A., Sainaghi P.P., Colangelo D. (2025). Extracellular Traps in Inflammation: Pathways and Therapeutic Targets. Life.

[46] Wang Y., Du C., Zhang Y., Zhu L. (2024). Composition and Function of Neutrophil Extracellular Traps. Biomolecules.

[47] Conceição-Silva F., Reis C.S.M., De Luca P.M., Leite-Silva J., Santiago M.A., Morrot A., Morgado F.N. (2021). The Immune System Throws Its Traps: Cells and Their Extracellular Traps in Disease and Protection. Cells.

[48] Liu M.L., Lyu X., Werth V.P. (2022). Recent progress in the mechanistic understanding of NET formation in neutrophils. FEBS J..

[49] Fuchs T.A., Abed U., Goosmann C., Hurwitz R., Schulze I., Wahn V., Weinrauch Y., Brinkmann V., Zychlinsky A. (2007). Novel cell death program leads to neutrophil extracellular traps. J. Cell Biol..

[50] Parker H.A., Jones H.M., Kaldor C.D., Hampton M.B., Winterbourn C.C. (2021). Neutrophil NET Formation with Microbial Stimuli Requires Late Stage NADPH Oxidase Activity. Antioxidants.

[51] Stoiber W., Obermayer A., Steinbacher P., Krautgartner W.D. (2015). The Role of Reactive Oxygen Species (ROS) in the Formation of Extracellular Traps (ETs) in Humans. Biomolecules.

[52] Brinkmann V., Reichard U., Goosmann C., Fauler B., Uhlemann Y., Weiss D.S., Weinrauch Y., Zychlinsky A. (2004). Neutrophil extracellular traps kill bacteria. Science.

[53] Wang H., Kim S.J., Lei Y., Wang S., Wang H., Huang H., Zhang H., Tsung A. (2024). Neutrophil extracellular traps in homeostasis and disease. Signal Transduct. Target. Ther..

[54] Ueki S., Konno Y., Takeda M., Moritoki Y., Hirokawa M., Matsuwaki Y., Honda K., Ohta N., Yamamoto S., Takagi Y. (2016). Eosinophil extracellular trap cell death-derived DNA traps: Their presence in secretions and functional attributes. J. Allergy Clin. Immunol..

[55] Robb C.T., Dyrynda E.A., Gray R.D., Rossi A.G., Smith V.J. (2014). Invertebrate extracellular phagocyte traps show that chromatin is an ancient defence weapon. Nat. Commun..

[56] Herre M., Cedervall J., Mackman N., Olsson A.K. (2023). Neutrophil extracellular traps in the pathology of cancer and other inflammatory diseases. Physiol. Rev..

[57] Boettcher M., Esser M., Trah J., Klohs S., Mokhaberi N., Wenskus J., Trochimiuk M., Appl B., Reinshagen K., Raluy L.P. (2020). Markers of neutrophil activation and extracellular traps formation are predictive of appendicitis in mice and humans: A pilot study. Sci. Rep..

[58] Mukherjee M., Lacy P., Ueki S. (2018). Eosinophil Extracellular Traps and Inflammatory Pathologies-Untangling the Web!. Front. Immunol..

[59] Yousefi S., Gold J.A., Andina N., Lee J.J., Kelly A.M., Kozlowski E., Schmid I., Straumann A., Reichenbach J., Gleich G.J. (2008). Catapult-like release of mitochondrial DNA by eosinophils contributes to antibacterial defense. Nat. Med..

[60] Zhang S., Wang Z. (2023). An Emerging Role of Extracellular Traps in Chronic Rhinosinusitis. Curr. Allergy Asthma Rep..

[61] Tomizawa H., Arima M., Miyabe Y., Furutani C., Kodama S., Ito K., Watanabe K., Hasegawa R., Nishiyama S., Hizuka K. (2025). Characteristics and Regulation of Human Eosinophil ETosis In Vitro. Am. J. Respir. Cell Mol. Biol..

[62] Fukuchi M., Miyabe Y., Furutani C., Saga T., Moritoki Y., Yamada T., Weller P.F., Ueki S. (2021). How to detect eosinophil ETosis (EETosis) and extracellular traps. Allergol. Int..

[63] Silva J.D.C., Thompson-Souza G.A., Barroso M.V., Neves J.S., Figueiredo R.T. (2021). Neutrophil and Eosinophil DNA Extracellular Trap Formation: Lessons From Pathogenic Fungi. Front. Microbiol..

[64] Gevaert E., Zhang N., Krysko O., Lan F., Holtappels G., De Ruyck N., Nauwynck H., Yousefi S., Simon H.U., Bachert C. (2017). Extracellular eosinophilic traps in association with *Staphylococcus aureus* at the site of epithelial barrier defects in patients with severe airway inflammation. J. Allergy Clin. Immunol..

[65] Ueki S., Hebisawa A., Kitani M., Asano K., Neves J.S. (2018). Allergic Bronchopulmonary Aspergillosis-A Luminal Hypereosinophilic Disease with Extracellular Trap Cell Death. Front. Immunol..

[66] Omokawa A., Ueki S., Kikuchi Y., Takeda M., Asano M., Sato K., Sano M., Ito H., Hirokawa M. (2018). Mucus plugging in allergic bronchopulmonary aspergillosis: Implication of the eosinophil DNA traps. Allergol. Int..

[67] Hwang C.S., Park S.C., Cho H.J., Park D.J., Yoon J.H., Kim C.H. (2019). Eosinophil extracellular trap formation is closely associated with disease severity in chronic rhinosinusitis regardless of nasal polyp status. Sci. Rep..

[68] Zhang S., Wang Z. (2024). Eosinophil extracellular traps in eosinophilic chronic rhinosinusitis induce Charcot-Leyden crystal formation and eosinophil recruitment. Biosci. Rep..

[69] Choi Y., Kim Y.M., Lee H.R., Mun J., Sim S., Lee D.H., Pham D.L., Kim S.H., Shin Y.S., Lee S.W. (2020). Eosinophil extracellular traps activate type 2 innate lymphoid cells through stimulating airway epithelium in severe asthma. Allergy.

[70] Zhang J., Liu X., Shi G.P., Chen W., Zhang S. (2026). Eosinophil extracellular traps: Heterogeneity of their stimuli, components, and functions. Trends Cell Biol..

[71] Yousefi S., Simon D., Simon H.U. (2012). Eosinophil extracellular DNA traps: Molecular mechanisms and potential roles in disease. Curr. Opin. Immunol..

[72] Fukuchi M., Kamide Y., Ueki S., Miyabe Y., Konno Y., Oka N., Takeuchi H., Koyota S., Hirokawa M., Yamada T. (2021). Eosinophil ETosis-Mediated Release of Galectin-10 in Eosinophilic Granulomatosis with Polyangiitis. Arthritis Rheumatol..

[73] Hoeksema M., van Eijk M., Haagsman H.P., Hartshorn K.L. (2016). Histones as mediators of host defense, inflammation and thrombosis. Future Microbiol..

[74] Kumar V. (2020). The Trinity of cGAS, TLR9, and ALRs Guardians of the Cellular Galaxy Against Host-Derived Self-DNA. Front. Immunol..

[75] Neves J.S., Weller P.F. (2009). Functional extracellular eosinophil granules: Novel implications in eosinophil immunobiology. Curr. Opin. Immunol..

[76] Dworski R., Simon H.U., Hoskins A., Yousefi S. (2011). Eosinophil and neutrophil extracellular DNA traps in human allergic asthmatic airways. J. Allergy Clin. Immunol..

[77] Muniz V.S., Silva J.C., Braga Y.A.V., Melo R.C.N., Ueki S., Takeda M., Hebisawa A., Asano K., Figueiredo R.T., Neves J.S. (2018). Eosinophils release extracellular DNA traps in response to *Aspergillus fumigatus*. J. Allergy Clin. Immunol..

[78] Silveira J.S., Antunes G.L., Gassen R.B., Breda R.V., Stein R.T., Pitrez P.M., da Cunha A.A. (2019). Respiratory syncytial virus increases eosinophil extracellular traps in a murine model of asthma. Asia Pac. Allergy.

[79] Silveira J.S., Antunes G.L., Kaiber D.B., da Costa M.S., Marques E.P., Ferreira F.S., Gassen R.B., Breda R.V., Wyse A.T.S., Pitrez P. (2019). Reactive oxygen species are involved in eosinophil extracellular traps release and in airway inflammation in asthma. J. Cell Physiol..

[80] Morshed M., Yousefi S., Stöckle C., Simon H.U., Simon D. (2012). Thymic stromal lymphopoietin stimulates the formation of eosinophil extracellular traps. Allergy.

[81] Schorn C., Janko C., Latzko M., Chaurio R., Schett G., Herrmann M. (2012). Monosodium urate crystals induce extracellular DNA traps in neutrophils, eosinophils, and basophils but not in mononuclear cells. Front. Immunol..

[82] Bai Y., Fang P., Li S., Xiao Z., Chen W., Li W., Wang X., Chen J., Li Y., Chen J. (2025). Accumulation of long-chain unsaturated fatty acids in the airway inflammatory microenvironment drives eosinophil etosis and corticosteroid resistance. Cell Commun. Signal.

[83] Kim H.J., Sim M.S., Lee D.H., Kim C., Choi Y., Park H.S., Chung I.Y. (2020). Lysophosphatidylserine induces eosinophil extracellular trap formation and degranulation: Implications in severe asthma. Allergy.

[84] Thiam H.R., Wong S.L., Qiu R., Kittisopikul M., Vahabikashi A., Goldman A.E., Goldman R.D., Wagner D.D., Waterman C.M. (2020). NETosis proceeds by cytoskeleton and endomembrane disassembly and PAD4-mediated chromatin decondensation and nuclear envelope rupture. Proc. Natl. Acad. Sci. USA.

[85] Choi Y., Le Pham D., Lee D.H., Lee S.H., Kim S.H., Park H.S. (2018). Biological function of eosinophil extracellular traps in patients with severe eosinophilic asthma. Exp. Mol. Med..

[86] Zwiers E., Montizaan D., Kip A., Waaijenberg K., Fichtinger P.S., Mathur S.K., Fujioka Y., Ueki S., van Es H., Chirivi R.G.S. (2025). Inhibition of EETosis with an anti-citrullinated histone antibody: A novel therapeutic approach for eosinophilic inflammatory disorders. Front. Immunol..

[87] Shen K., Zhang M., Zhao R., Li Y., Li C., Hou X., Sun B., Liu B., Xiang M., Lin J. (2023). Eosinophil extracellular traps in asthma: Implications for pathogenesis and therapy. Respir. Res..

[88] Martinod K., Denorme F., Meyers S., Crescente M., Van Bruggen S., Stroobants M., Siegel P.M., Grandhi R., Glatz K., Witsch T. (2024). Involvement of peptidylarginine deiminase 4 in eosinophil extracellular trap formation and contribution to citrullinated histone signal in thrombi. J. Thromb. Haemost..

[89] Germic N., Fettrelet T., Stojkov D., Hosseini A., Horn M.P., Karaulov A., Simon D., Yousefi S., Simon H.U. (2021). The Release Kinetics of Eosinophil Peroxidase and Mitochondrial DNA Is Different in Association with Eosinophil Extracellular Trap Formation. Cells.

[90] Yoon J., Ponikau J.U., Lawrence C.B., Kita H. (2008). Innate antifungal immunity of human eosinophils mediated by a beta 2 integrin, CD11b. J. Immunol..

[91] Huang L., Appleton J.A. (2016). Eosinophils in Helminth Infection: Defenders and Dupes. Trends Parasitol..

[92] Ehrens A., Lenz B., Neumann A.L., Giarrizzo S., Reichwald J.J., Frohberger S.J., Stamminger W., Buerfent B.C., Fercoq F., Martin C. (2021). Microfilariae Trigger Eosinophil Extracellular DNA Traps in a Dectin-1-Dependent Manner. Cell Rep..

[93] Hosoki K., Nakamura A., Nagao M., Hiraguchi Y., Tanida H., Tokuda R., Wada H., Nobori T., Suga S., Fujisawa T. (2012). *Staphylococcus aureus* directly activates eosinophils via platelet-activating factor receptor. J. Leukoc. Biol..

[94] Persson T., Andersson P., Bodelsson M., Laurell M., Malm J., Egesten A. (2001). Bactericidal activity of human eosinophilic granulocytes against *Escherichia coli*. Infect. Immun..

[95] Shin S.-H., Ye M.-K., Park J., Geum S.-Y. (2022). Immunopathologic Role of Eosinophils in Eosinophilic Chronic Rhinosinusitis. Int. J. Mol. Sci..

[96] Kline S.N., Orlando N.A., Lee A.J., Wu M.J., Zhang J., Youn C., Feller L.E., Pontaza C., Dikeman D., Limjunyawong N. (2024). *Staphylococcus aureus* proteases trigger eosinophil-mediated skin inflammation. Proc. Natl. Acad. Sci. USA.

[97] Wang Z., Hülpüsch C., Traidl-Hoffmann C., Reiger M., Schloter M. (2024). Understanding the role of *Staphylococcus aureus* in atopic dermatitis: Strain diversity, microevolution, and prophage influences. Front. Med..

[98] Krishack P.A., Louviere T.J., Decker T.S., Kuzel T.G., Greenberg J.A., Camacho D.F., Hrusch C.L., Sperling A.I., Verhoef P.A. (2019). Protection against *Staphylococcus aureus* bacteremia-induced mortality depends on ILC2s and eosinophils. JCI Insight.

[99] Severn M.M., Horswill A.R. (2023). *Staphylococcus epidermidis* and its dual lifestyle in skin health and infection. Nat. Rev. Microbiol..

[100] Kim H.B., Alexander H., Um J.Y., Chung B.Y., Park C.W., Flohr C., Kim H.O. (2025). Skin Microbiome Dynamics in Atopic Dermatitis: Understanding Host-Microbiome Interactions. Allergy Asthma Immunol. Res..

[101] Mueller M., Rausch-Phung E.A., Tainter C.R. (2026). *Escherichia coli* Infection. StatPearls.

[102] Lekki-Jóźwiak J., Bąska P. (2023). The Roles of Various Immune Cell Populations in Immune Response against Helminths. Int. J. Mol. Sci..

[103] Muñoz-Caro T., Rubio R.M., Silva L.M., Magdowski G., Gärtner U., McNeilly T.N., Taubert A., Hermosilla C. (2015). Leucocyte-derived extracellular trap formation significantly contributes to *Haemonchus contortus* larval entrapment. Parasit. Vectors.

[104] Shin S.H., Ye M.K., Lee D.W., Geum S.Y. (2023). Immunopathologic Role of Fungi in Chronic Rhinosinusitis. Int. J. Mol. Sci..

[105] Figueiredo R.T., Neves J.S. (2018). Eosinophils in fungal diseases: An overview. J. Leukoc. Biol..

[106] Inoue Y., Matsuwaki Y., Shin S.H., Ponikau J.U., Kita H. (2005). Nonpathogenic, environmental fungi induce activation and degranulation of human eosinophils. J. Immunol..

[107] Chaudhary N., Marr K.A. (2011). Impact of *Aspergillus fumigatus* in allergic airway diseases. Clin. Transl. Allergy.

[108] Barroso M.V., Gropillo I., Detoni M.A.A., Thompson-Souza G.A., Muniz V.S., Vasconcelos C.R.I., Figueiredo R.T., Melo R.C.N., Neves J.S. (2021). Structural and Signaling Events Driving *Aspergillus fumigatus*-Induced Human Eosinophil Extracellular Trap Release. Front. Microbiol..

[109] Simon D., Hoesli S., Roth N., Staedler S., Yousefi S., Simon H.U. (2011). Eosinophil extracellular DNA traps in skin diseases. J. Allergy Clin. Immunol..

[110] Simon D., Radonjic-Hösli S., Straumann A., Yousefi S., Simon H.U. (2015). Active eosinophilic esophagitis is characterized by epithelial barrier defects and eosinophil extracellular trap formation. Allergy.

[111] Ma R., Ortiz Serrano T.P., Davis J., Prigge A.D., Ridge K.M. (2020). The cGAS-STING pathway: The role of self-DNA sensing in inflammatory lung disease. FASEB J..

[112] Campoccia D., Montanaro L., Arciola C.R. (2021). Extracellular DNA (eDNA). A Major Ubiquitous Element of the Bacterial Biofilm Architecture. Int. J. Mol. Sci..

[113] Miyabe Y., Fukuchi M., Tomizawa H., Nakamura Y., Jikei M., Matsuwaki Y., Arima M., Konno Y., Moritoki Y., Takeda M. (2024). Aggregated eosinophils and neutrophils characterize the properties of mucus in chronic rhinosinusitis. J. Allergy Clin. Immunol..

[114] Cha H., Lim H.S., Park J.A., Jo A., Ryu H.T., Kim D.W., Kim J.K., Hong S.N., Shin H.W., Kim D.W. (2023). Effects of Neutrophil and Eosinophil Extracellular Trap Formation on Refractoriness in Chronic Rhinosinusitis with Nasal Polyps. Allergy Asthma Immunol. Res..

[115] Ohta N., Ueki S., Konno Y., Hirokawa M., Kubota T., Tomioka-Matsutani S., Suzuki T., Ishida Y., Kawano T., Miyasaka T. (2018). ETosis-derived DNA trap production in middle ear effusion is a common feature of eosinophilic otitis media. Allergol. Int..

[116] Ueki S., Ohta N., Takeda M., Konno Y., Hirokawa M. (2017). Eosinophilic Otitis Media: The Aftermath of Eosinophil Extracellular Trap Cell Death. Curr. Allergy Asthma Rep..

[117] Vlaminck S., Acke F., Scadding G.K., Lambrecht B.N., Gevaert P. (2021). Pathophysiological and Clinical Aspects of Chronic Rhinosinusitis: Current Concepts. Front. Allergy.

[118] Fokkens W.J., Lund V.J., Hopkins C., Hellings P.W., Kern R., Reitsma S., Toppila-Salmi S., Bernal-Sprekelsen M., Mullol J., Alobid I. (2020). European Position Paper on Rhinosinusitis and Nasal Polyps 2020. Rhinology.

[119] Bachert C., Hicks A., Gane S., Peters A.T., Gevaert P., Nash S., Horowitz J.E., Sacks H., Jacob-Nara J.A. (2024). The interleukin-4/interleukin-13 pathway in type 2 inflammation in chronic rhinosinusitis with nasal polyps. Front. Immunol..

[120] Gevaert P., Han J.K., Smith S.G., Sousa A.R., Howarth P.H., Yancey S.W., Chan R., Bachert C. (2022). The roles of eosinophils and interleukin-5 in the pathophysiology of chronic rhinosinusitis with nasal polyps. Int. Forum Allergy Rhinol..

[121] Zielińska-Bliźniewska H., Paprocka-Zjawiona M., Merecz-Sadowska A., Zajdel R., Bliźniewska-Kowalska K., Malinowska K. (2022). Serum IL-5, POSTN and IL-33 levels in chronic rhinosinusitis with nasal polyposis correlate with clinical severity. BMC Immunol..

[122] Yun Y., Kanda A., Kobayashi Y., Van Bui D., Suzuki K., Sawada S., Baba K., Yagi M., Asako M., Okazaki H. (2020). Increased CD69 expression on activated eosinophils in eosinophilic chronic rhinosinusitis correlates with clinical findings. Allergol. Int..

[123] Bochner B.S., Stevens W.W. (2021). Biology and Function of Eosinophils in Chronic Rhinosinusitis with or Without Nasal Polyps. Allergy Asthma Immunol. Res..

[124] Idler B.M., Iijima K., Ochkur S.I., Jacobsen E.A., Rank M.A., Kita H., Lal D. (2024). Eosinophil Peroxidase: A Biomarker for Eosinophilic Chronic Rhinosinusitis Agnostic of Polyp Status. Laryngoscope.

[125] Miyata J., Fukunaga K., Kawashima Y., Watanabe T., Saitoh A., Hirosaki T., Araki Y., Kikawada T., Betsuyaku T., Ohara O. (2019). Dysregulated fatty acid metabolism in nasal polyp-derived eosinophils from patients with chronic rhinosinusitis. Allergy.

[126] Ibrahim S., Mahmoud K.G.M., Sosa A.S.A., Sakr A.A.M., El-Naby A.A.H., Nawito M.F. (2020). Establishment a protocol for total RNA isolation from buffalo fresh and frozen semen for molecular applications. Andrologia.

[127] Liu W., Shi L., Feng Y. (2024). Advance in the pathogenesis of otitis media with effusion induced by platelet-activating factor. Sci. Prog..

[128] Guo L., Li T., Duan Q., Duan Y., Liu W., Ma N., Liu S., Wang X., Zhang S., Tian E. (2025). Higher levels of neutrophil extracellular traps in mucoid middle ear effusion obtained from otitis media with effusion in children. Ann. Med..

[129] Wright E.D., Hurst D., Miotto D., Giguere C., Hamid Q. (2000). Increased expression of major basic protein (MBP) and interleukin-5(IL-5) in middle ear biopsy specimens from atopic patients with persistent otitis media with effusion. Otolaryngol. Head. Neck Surg..

[130] Xiao Y., Wang X., Liu M., Fu X., Zhang N., Yang W., Ge J., Li Y., Ma D., Ma J. (2025). Targeting Neutrophil/Eosinophil Extracellular Traps by Aptamer-Functionalized Nanosheets to Overcome Recalcitrant Inflammatory Disorders. Adv. Sci..

[131] Suikkila A., Lyly A., Savinko T., Vento S.I., Saarinen R., Hafrén L. (2024). Inflammatory Cytokines in Middle Ear Effusion of Patients with Asthma, Chronic Rhinosinusitis with Nasal Polyps with or Without NSAID Intolerance. Otol. Neurotol..

[132] Uribe Echevarría L., Leimgruber C., García González J., Nevado A., Álvarez R., García L.N., Quintar A.A., Maldonado C.A. (2017). Evidence of eosinophil extracellular trap cell death in COPD: Does it represent the trigger that switches on the disease?. Int. J. Chronic Obstr. Pulm. Dis..

[133] Takeda M., Sakamoto S., Ueki S., Miyabe Y., Fukuchi M., Okuda Y., Asano M., Sato K., Nakayama K. (2021). Eosinophil extracellular traps in a patient with chronic eosinophilic pneumonia. Asia Pac. Allergy.

[134] Reddel H.K., Bacharier L.B., Bateman E.D., Brightling C.E., Brusselle G.G., Buhl R., Cruz A.A., Duijts L., Drazen J.M., FitzGerald J.M. (2022). Global Initiative for Asthma Strategy 2021: Executive Summary and Rationale for Key Changes. Am. J. Respir. Crit. Care Med..

[135] Gu W., Huang C., Chen G., Kong W., Zhao L., Jie H., Zhen G. (2024). The role of extracellular traps released by neutrophils, eosinophils, and macrophages in asthma. Respir. Res..

[136] Luo J., Chen W., Liu W., Jiang S., Ye Y., Shrimanker R., Hynes G., Klenerman P., Pavord I.D., Xue L. (2024). IL-5 antagonism reverses priming and activation of eosinophils in severe eosinophilic asthma. Mucosal Immunol..

[137] Rael E.L., Lockey R.F. (2011). Interleukin-13 signaling and its role in asthma. World Allergy Organ. J..

[138] Cunha A.A., Porto B.N., Nuñez N.K., Souza R.G., Vargas M.H., Silveira J.S., Souza T.T., Jaeger N., Pitrez P.M. (2014). Extracellular DNA traps in bronchoalveolar fluid from a murine eosinophilic pulmonary response. Allergy.

[139] Silveira J.S., Antunes G.L., Kaiber D.B., da Costa M.S., Ferreira F.S., Marques E.P., Schmitz F., Gassen R.B., Breda R.V., Wyse A.T.S. (2020). Autophagy induces eosinophil extracellular traps formation and allergic airway inflammation in a murine asthma model. J. Cell Physiol..

[140] Lu Y., Huang Y., Li J., Huang J., Zhang L., Feng J., Li J., Xia Q., Zhao Q., Huang L. (2021). Eosinophil extracellular traps drive asthma progression through neuro-immune signals. Nat. Cell Biol..

[141] Granger V., Taillé C., Roach D., Letuvé S., Dupin C., Hamidi F., Noël B., Neukirch C., Aubier M., Pretolani M. (2020). Circulating neutrophil and eosinophil extracellular traps are markers of severe asthma. Allergy.

[142] da Cunha A.A., Nuñez N.K., de Souza R.G., Moraes Vargas M.H., Silveira J.S., Antunes G.L., Durante Lda S., Porto B.N., Marczak E.S., Jones M.H. (2016). Recombinant human deoxyribonuclease therapy improves airway resistance and reduces DNA extracellular traps in a murine acute asthma model. Exp. Lung Res..

[143] Dunican E.M., Elicker B.M., Gierada D.S., Nagle S.K., Schiebler M.L., Newell J.D., Raymond W.W., Lachowicz-Scroggins M.E., Di Maio S., Hoffman E.A. (2018). Mucus plugs in patients with asthma linked to eosinophilia and airflow obstruction. J. Clin. Investig..

[144] Lee D.H., Jang J.H., Sim S., Choi Y., Park H.S. (2022). Epithelial Autoantigen-Specific IgG Antibody Enhances Eosinophil Extracellular Trap Formation in Severe Asthma. Allergy Asthma Immunol. Res..

[145] Cazzola M., Bajpai J., Calzetta L., Matera M.G., Rogliani P. (2026). GOLD 2026: Transforming COPD Management with Early Intervention, Multi-dimensional Assessment, and Personalized Care. Drugs.

[146] Papaporfyriou A., Bakakos P., Hillas G., Papaioannou A.I., Loukides S. (2022). Blood eosinophils in COPD: Friend or foe?. Expert. Rev. Respir. Med..

[147] Bafadhel M., McKenna S., Terry S., Mistry V., Pancholi M., Venge P., Lomas D.A., Barer M.R., Johnston S.L., Pavord I.D. (2012). Blood eosinophils to direct corticosteroid treatment of exacerbations of chronic obstructive pulmonary disease: A randomized placebo-controlled trial. Am. J. Respir. Crit. Care Med..

[148] Xuan N., Zhao J., Kang Z., Cui W., Tian B.-p. (2024). Neutrophil extracellular traps and their implications in airway inflammatory diseases. Front. Med..

[149] Carbone R.G., Puppo F., Mattar E., Roden A.C., Hirani N. (2024). Acute and chronic eosinophilic pneumonia: An overview. Front. Med..

[150] Fijolek J., Radzikowska E. (2023). Eosinophilic granulomatosis with polyangiitis—Advances in pathogenesis, diagnosis, and treatment. Front. Med..

[151] Hashimoto T., Ueki S., Kamide Y., Miyabe Y., Fukuchi M., Yokoyama Y., Furukawa T., Azuma N., Oka N., Takeuchi H. (2021). Increased Circulating Cell-Free DNA in Eosinophilic Granulomatosis with Polyangiitis: Implications for Eosinophil Extracellular Traps and Immunothrombosis. Front. Immunol..

[152] Hellmark T., Ohlsson S., Pettersson Å., Hansson M., Johansson Å.C.M. (2019). Eosinophils in anti-neutrophil cytoplasmic antibody associated vasculitis. BMC Rheumatol..

[153] Emmi G., Bettiol A., Gelain E., Bajema I.M., Berti A., Burns S., Cid M.C., Cohen Tervaert J.W., Cottin V., Durante E. (2023). Evidence-Based Guideline for the diagnosis and management of eosinophilic granulomatosis with polyangiitis. Nat. Rev. Rheumatol..

[154] Lazzeroni M., Longoni V., Schiavo P., Bizzi E., Brucato A., Gramellini G., Borin M., Dragonetti A., Gaffuri M., Lentini M. (2025). Anti-IL5/IL-5 receptor therapies for eosinophilic granulomatosis with polyangiitis: An updated Systematic Review. Front. Immunol..

[155] Watanabe R., Hashimoto M. (2023). Eosinophilic Granulomatosis with Polyangiitis: Latest Findings and Updated Treatment Recommendations. J. Clin. Med..

[156] Nolasco S., Portacci A., Campisi R., Buonamico E., Pelaia C., Benfante A., Triggiani M., Spadaro G., Caiaffa M.F., Scioscia G. (2023). Effectiveness and safety of anti-IL-5/Rα biologics in eosinophilic granulomatosis with polyangiitis: A two-year multicenter observational study. Front. Immunol..

[157] Huang S.U., O’Sullivan K.M. (2022). The Expanding Role of Extracellular Traps in Inflammation and Autoimmunity: The New Players in Casting Dark Webs. Int. J. Mol. Sci..

[158] Tomizawa H., Yamada Y., Arima M., Miyabe Y., Fukuchi M., Hikichi H., Melo R.C.N., Yamada T., Ueki S. (2022). Galectin-10 as a Potential Biomarker for Eosinophilic Diseases. Biomolecules.

[159] Ferreira J., Gonçalves S., Duarte T., Quintal J., Coelho R., Costa C. (2024). The three stages of eosinophilic cardiac damage: A series of case reports. J. Cardiol. Cases.

[160] Liu X., Liu Y., Qiu Y., Zhang J., Xu K., Guo T., Wang Y., Yang X., Wang S., Wang Y. (2026). Al^18^F-FAPI PET/CT in hypereosinophilic syndrome with cardiac involvement and its potential association with eosinophil extracellular traps. Eur. J. Nucl. Med. Mol. Imaging.

[161] Siebermair J., Köhler M.I., Kupusovic J., Nekolla S.G., Kessler L., Ferdinandus J., Guberina N., Stuschke M., Grafe H., Siveke J.T. (2021). Cardiac fibroblast activation detected by Ga-68 FAPI PET imaging as a potential novel biomarker of cardiac injury/remodeling. J. Nucl. Cardiol..

[162] Linch S.N., Danielson E.T., Kelly A.M., Tamakawa R.A., Lee J.J., Gold J.A. (2012). Interleukin 5 is protective during sepsis in an eosinophil-independent manner. Am. J. Respir. Crit. Care Med..

[163] Retter A., Singer M., Annane D. (2025). “The NET effect”: Neutrophil extracellular traps-a potential key component of the dysregulated host immune response in sepsis. Crit. Care.

[164] Li Y., Wan D., Luo X., Song T., Wang Y., Yu Q., Jiang L., Liao R., Zhao W., Su B. (2021). Circulating Histones in Sepsis: Potential Outcome Predictors and Therapeutic Targets. Front. Immunol..

[165] Radonjic-Hoesli S., Brüggen M.C., Feldmeyer L., Simon H.U., Simon D. (2021). Eosinophils in skin diseases. Semin. Immunopathol..

[166] Weihrauch T., Melo R.C.N., Gray N., Voehringer D., Weller P.F., Raap U. (2024). Eosinophil extracellular vesicles and DNA traps in allergic inflammation. Front. Allergy.

[167] Ahmed A., Cahn B., Haber R. (2025). Wells syndrome: Emerging triggers and treatments- an updated systematic review. Arch. Dermatol. Res..

[168] Brehmer-Andersson E., Kaaman T., Skog E., Frithz A. (1986). The histopathogenesis of the flame figure in Wells’ syndrome based on five cases. Acta Derm. Venereol..

[169] Remijsen Q., Kuijpers T.W., Wirawan E., Lippens S., Vandenabeele P., Vanden Berghe T. (2011). Dying for a cause: NETosis, mechanisms behind an antimicrobial cell death modality. Cell Death Differ..

[170] Xu J., Zhang X., Pelayo R., Monestier M., Ammollo C.T., Semeraro F., Taylor F.B., Esmon N.L., Lupu F., Esmon C.T. (2009). Extracellular histones are major mediators of death in sepsis. Nat. Med..

[171] Ueki S., Tokunaga T., Fujieda S., Honda K., Hirokawa M., Spencer L.A., Weller P.F. (2016). Eosinophil ETosis and DNA Traps: A New Look at Eosinophilic Inflammation. Curr. Allergy Asthma Rep..

[172] Deotto M.L., Spiller A., Sernicola A., Alaibac M. (2022). Bullous pemphigoid: An immune disorder related to aging (Review). Exp. Ther. Med..

[173] Ramcke T., Vicari E., Bolduan V., Enk A., Hadaschik E. (2022). Bullous pemphigoid (BP) patients with selective IgG autoreactivity against BP230: Review of a rare but valuable cohort with impact on the comprehension of the pathogenesis of BP. J. Dermatol. Sci..

[174] Limberg M.M., Weihrauch T., Gray N., Ernst N., Hartmann K., Raap U. (2023). Eosinophils, Basophils, and Neutrophils in Bullous Pemphigoid. Biomolecules.

[175] Jones V.A., Patel P.M., Amber K.T. (2021). Eosinophils in bullous pemphigoid. Panminerva Med..

[176] Kowalski E.H., Kneibner D., Kridin K., Amber K.T. (2019). Serum and blister fluid levels of cytokines and chemokines in pemphigus and bullous pemphigoid. Autoimmun. Rev..

[177] Lu M., DiBernardo E., Parks E., Fox H., Zheng S.Y., Wayne E. (2021). The Role of Extracellular Vesicles in the Pathogenesis and Treatment of Autoimmune Disorders. Front. Immunol..

[178] de Graauw E., Sitaru C., Horn M., Borradori L., Yousefi S., Simon H.U., Simon D. (2017). Evidence for a role of eosinophils in blister formation in bullous pemphigoid. Allergy.

[179] Fang H., Shao S., Jiang M., Dang E., Shen S., Zhang J., Qiao P., Li C., Wang G. (2018). Proinflammatory role of blister fluid-derived exosomes in bullous pemphigoid. J. Pathol..

[180] Hehar N.K., Chigbu D.I. (2024). Vernal Keratoconjunctivitis: Immunopathological Insights and Therapeutic Applications of Immunomodulators. Life.

[181] Shiraki Y., Shoji J., Inada N. (2016). Clinical Usefulness of Monitoring Expression Levels of CCL24 (Eotaxin-2) mRNA on the Ocular Surface in Patients with Vernal Keratoconjunctivitis and Atopic Keratoconjunctivitis. J. Ophthalmol..

[182] Provost V., Larose M.C., Langlois A., Rola-Pleszczynski M., Flamand N., Laviolette M. (2013). CCL26/eotaxin-3 is more effective to induce the migration of eosinophils of asthmatics than CCL11/eotaxin-1 and CCL24/eotaxin-2. J. Leukoc. Biol..

[183] Fernandez A., Asbell P., Roy N. (2022). Emerging therapies targeting eosinophil-mediated inflammation in chronic allergic conjunctivitis. Ocul. Surf..

[184] Bao J., Wen Y., Tian L., Jie Y. (2025). Decoding Allergic Conjunctivitis: Latest Perspectives on Etiological Drivers and Immunopathological Mechanisms. Clin. Rev. Allergy Immunol..

[185] Onoue M., Saga A., Adachi K., Asada Y., Hirakata T., Iwamoto S., Ueki S., Ebihara N., Matsuda A. (2024). Eosinophil extracellular trap formation in the giant papillae of atopic keratoconjunctivitis and vernal keratoconjunctivitis. Allergol. Int..

[186] Melhem H., Niess J.H. (2024). Eosinophilic Esophagitis and Inflammatory Bowel Disease: What Are the Differences?. Int. J. Mol. Sci..

[187] Jacobs I., Ceulemans M., Wauters L., Breynaert C., Vermeire S., Verstockt B., Vanuytsel T. (2021). Role of Eosinophils in Intestinal Inflammation and Fibrosis in Inflammatory Bowel Disease: An Overlooked Villain?. Front. Immunol..

[188] Dellon E.S., Muir A.B., Katzka D.A., Shah S.C., Sauer B.G., Aceves S.S., Furuta G.T., Gonsalves N., Hirano I. (2025). ACG Clinical Guideline: Diagnosis and Management of Eosinophilic Esophagitis. Am. J. Gastroenterol..

[189] Wu L., Oshima T., Li M., Tomita T., Fukui H., Watari J., Miwa H. (2018). Filaggrin and tight junction proteins are crucial for IL-13-mediated esophageal barrier dysfunction. Am. J. Physiol. Gastrointest. Liver Physiol..

[190] McGowan E.C., Singh R., Katzka D.A. (2023). Barrier Dysfunction in Eosinophilic Esophagitis. Curr. Gastroenterol. Rep..

[191] Racca F., Pellegatta G., Cataldo G., Vespa E., Carlani E., Pelaia C., Paoletti G., Messina M.R., Nappi E., Canonica G.W. (2021). Type 2 Inflammation in Eosinophilic Esophagitis: From Pathophysiology to Therapeutic Targets. Front. Physiol..

[192] Akkoç T. (2025). Epithelial barrier dysfunction and microbial dysbiosis: Exploring the pathogenesis and therapeutic strategies for Crohn’s disease. Tissue Barriers.

[193] Xu J., Guo J., Liu T., Yang C., Meng Z., Libby P., Zhang J., Shi G.P. (2025). Differential roles of eosinophils in cardiovascular disease. Nat. Rev. Cardiol..

[194] Marx C., Novotny J., Salbeck D., Zellner K.R., Nicolai L., Pekayvaz K., Kilani B., Stockhausen S., Bürgener N., Kupka D. (2019). Eosinophil-platelet interactions promote atherosclerosis and stabilize thrombosis with eosinophil extracellular traps. Blood.

[195] Kong P., Cui Z.Y., Huang X.F., Zhang D.D., Guo R.J., Han M. (2022). Inflammation and atherosclerosis: Signaling pathways and therapeutic intervention. Signal Transduct. Target. Ther..

[196] Mackman N. (2019). Eosinophils, atherosclerosis, and thrombosis. Blood.

[197] Niccoli G., Ferrante G., Cosentino N., Conte M., Belloni F., Marino M., Bacà M., Montone R.A., Sabato V., Schiavino D. (2010). Eosinophil cationic protein: A new biomarker of coronary atherosclerosis. Atherosclerosis.

[198] Sundström J., Söderholm M., Borné Y., Nilsson J., Persson M., Östling G., Melander O., Orho-Melander M., Engström G. (2017). Eosinophil Cationic Protein, Carotid Plaque, and Incidence of Stroke. Stroke.

[199] Meng Z., Zhang S., Li W., Wang Y., Wang M., Liu X., Liu C.L., Liao S., Liu T., Yang C. (2023). Cationic proteins from eosinophils bind bone morphogenetic protein receptors promoting vascular calcification and atherogenesis. Eur. Heart J..

[200] Shah S.A., Page C.P., Pitchford S.C. (2017). Platelet-Eosinophil Interactions As a Potential Therapeutic Target in Allergic Inflammation and Asthma. Front. Med..

[201] Ming B., Zhong J., Dong L. (2022). Role of eosinophilia in IgG4-related disease. Clin. Exp. Rheumatol..

[202] Arias-Intriago M., Gomolin T., Jaramillo F., Cruz-Enríquez A.C., Lara-Arteaga A.L., Tello-De-la-Torre A., Ortiz-Prado E., Izquierdo-Condoy J.S. (2025). IgG4-Related Disease: Current and Future Insights into Pathological Diagnosis. Int. J. Mol. Sci..

[203] Amano S., Nishiguchi S., Mochida Y., Teshima S., Ueki S. (2025). IgG4-related disease with eosinophil extracellular traps. QJM Int. J. Med..

[204] Zhang X., Zhang P., Li J., He Y., Fei Y., Peng L., Shi Q., Zhang W., Zhao Y. (2019). Different clinical patterns of IgG4-RD patients with and without eosinophilia. Sci. Rep..

[205] Grisaru-Tal S., Jacobsen E.A., Munitz A. (2025). Evolving role for eosinophils in cancer: From bench to bedside. Trends Cancer.

[206] Hu G., Wang S., Zhong K., Xu F., Huang L., Chen W., Cheng P. (2020). Tumor-associated tissue eosinophilia predicts favorable clinical outcome in solid tumors: A meta-analysis. BMC Cancer.

[207] Caruso R., Caruso V., Rigoli L. (2025). Eosinophil ETosis and Cancer: Ultrastructural Evidence and Oncological Implications. Cancers.

[208] Lopez-Perez D., Prados-Lopez B., Galvez J., Leon J., Carazo A. (2024). Eosinophils in Colorectal Cancer: Emerging Insights into Anti-Tumoral Mechanisms and Clinical Implications. Int. J. Mol. Sci..

[209] Ammann N.L., Schwietzer Y.F., Mess C., Stadler J.C., Geidel G., Kött J., Pantel K., Schneider S.W., Utikal J., Bauer A.T. (2022). Activated Eosinophils Predict Longer Progression-Free Survival under Immune Checkpoint Inhibition in Melanoma. Cancers.

[210] Lee T.L., Chen T.H., Kuo Y.J., Lan H.Y., Yang M.H., Chu P.Y. (2023). Tumor-associated tissue eosinophilia promotes angiogenesis and metastasis in head and neck squamous cell carcinoma. Neoplasia.

[211] Zhang J., Qiu Y., Liu Y., Mo S., Nie M., Zhang H., Liu X., Chen W. (2025). Eosinophil extracellular traps formation is correlated with cancer prognosis by tumor microenvironment remodeling. Sci. Rep..

[212] Gilon M., Boulet D., Bours V., Jerusalem G., Josse C., Gennigens C. (2026). Eosinophils in solid cancers: Sentinels, predictors, and therapeutic allies. J. Exp. Clin. Cancer Res..

[213] Francischetti I.M.B., Alejo J.C., Sivanandham R., Davies-Hill T., Fetsch P., Pandrea I., Jaffe E.S., Pittaluga S. (2021). Neutrophil and Eosinophil Extracellular Traps in Hodgkin Lymphoma. Hemasphere.

[214] Caruso R., Caruso V., Rigoli L. (2025). Eosinophil cytolysis with or without ETosis in four cases of human gastric cancer: A comparative ultrastructural study. Explor. Target. Antitumor Ther..

[215] Yang L., Liu Q., Zhang X., Liu X., Zhou B., Chen J., Huang D., Li J., Li H., Chen F. (2020). DNA of neutrophil extracellular traps promotes cancer metastasis via CCDC25. Nature.

[216] Liu R., Zhao E., Wang F., Cui H. (2020). CCDC25: Precise navigator for neutrophil extracellular traps on the prometastatic road. Signal Transduct. Target. Ther..

[217] Hennigan K., Conroy P.J., Walsh M.T., Amin M., O’Kennedy R., Ramasamy P., Gleich G.J., Siddiqui Z., Glynn S., McCabe O. (2016). Eosinophil peroxidase activates cells by HER2 receptor engagement and β1-integrin clustering with downstream MAPK cell signaling. Clin. Immunol..

[218] Elmusrati A., Wang J., Wang C.Y. (2021). Tumor microenvironment and immune evasion in head and neck squamous cell carcinoma. Int. J. Oral. Sci..

[219] Zhao C., Zhu H., Tian Y., Sun Y., Zhang Z. (2024). SPINK5 is a key regulator of eosinophil extracellular traps in head and neck squamous cell carcinoma. Discov. Oncol..

[220] Dorta R.G., Landman G., Kowalski L.P., Lauris J.R., Latorre M.R., Oliveira D.T. (2002). Tumour-associated tissue eosinophilia as a prognostic factor in oral squamous cell carcinomas. Histopathology.

[221] Ghaffari S., Rezaei N. (2023). Eosinophils in the tumor microenvironment: Implications for cancer immunotherapy. J. Transl. Med..

[222] Wali Z., Neha, Shamsi A., Tasqeruddin S., Anwar S. (2025). The SPINK Protein Family in Cancer: Emerging Roles in Tumor Progression, Therapeutic Resistance, and Precision Oncology. Pharmaceuticals.

[223] Lv Z., Wu K., Qin X., Yuan J., Yan M., Zhang J., Wang L., Ji T., Cao W., Chen W. (2020). A Novel Tumor Suppressor SPINK5 Serves as an Independent Prognostic Predictor for Patients with Head and Neck Squamous Cell Carcinoma. Cancer Manag. Res..

[224] Gordhandas S., Zammarrelli W.A., Rios-Doria E.V., Green A.K., Makker V. (2023). Current Evidence-Based Systemic Therapy for Advanced and Recurrent Endometrial Cancer. J. Natl. Compr. Canc Netw..

[225] Tan Z., Sheng B., Chen L., Dong H., Deng Y., Li Y., Liu C., Wang H., Yang Z., Xie T. (2025). Inflammation-driven mechanisms in endometrial cancer: Pathways from inflammatory microenvironment remodeling to immune escape. Front. Immunol..

[226] Ni J., Mao Y., Ma Q., Wu Z., Su M. (2025). S100A9 regulates eosinophil extracellular trap and activates NF-κB signaling in endometrial cancer: A machine learning-based biomarker discovery. Cancer Cell Int..

[227] Song D.H., Kim M.H., Yang J., Jo H.C., Park J.E., Baek J.C. (2025). Differential Expression of S100A8 in Tumor and Immune Compartments of Endometrial Carcinoma and Its Clinical Relevance. Medicina.

[228] Markowitz J., Carson W.E. (2013). Review of S100A9 biology and its role in cancer. Biochim. Biophys. Acta.

[229] Caruso R., Caruso V., Rigoli L. (2023). Ultrastructural Evidence of Eosinophil Clustering and ETosis in Association with Damage to Single Tumour Cells in a Case of Poorly Cohesive NOS Gastric Carcinoma. Eur. J. Case Rep. Intern. Med..

[230] Cellini A., Scarmozzino F., Angotzi F., Ruggeri E., Dei Tos A.P., Trentin L., Pizzi M., Visentin A. (2023). Tackling the dysregulated immune-checkpoints in classical *Hodgkin lymphoma*: Bidirectional regulations between the microenvironment and Hodgkin/Reed-Sternberg cells. Front. Oncol..

[231] Danielson D.T., Aguilera N.S., Auerbach A. (2025). Head and Neck Classic Hodgkin, T and NK Lymphomas with Eosinophilia. Head. Neck Pathol..

[232] Boogaard R., Smit F., Schornagel R., Vaessen-Verberne A.A., Kouwenberg J.M., Hekkelaan M., Hendriks T., Feith S.W., Hop W.C., de Jongste J.C. (2008). Recombinant human deoxyribonuclease for the treatment of acute asthma in children. Thorax.

[233] Silverman R.A., Foley F., Dalipi R., Kline M., Lesser M. (2012). The use of rhDNAse in severely ill, non-intubated adult asthmatics refractory to bronchodilators: A pilot study. Respir. Med..

[234] Negewo N.A., Niessen N.M., Baines P.J., Williams E.J., Fibbens N., Simpson J.L., McDonald V.M., Berthon B.S., Gibson P.G., Baines K.J. (2025). Targeted DNase treatment of obstructive lung disease: A pilot randomised controlled trial. ERJ Open Res..

[235] Liegeois M.A., Hsieh A., Al-Fouadi M., Charbit A.R., Yang C.X., Hackett T.L., Fahy J.V. (2025). Cellular and molecular features of asthma mucus plugs provide clues about their formation and persistence. J. Clin. Investig..

[236] Dasgupta B., King M. (1996). Reduction in viscoelasticity in cystic fibrosis sputum in vitro using combined treatment with nacystelyn and rhDNase. Pediatr. Pulmonol..

[237] Sun F., Tai S., Lim T., Baumann U., King M. (2002). Additive effect of dornase alfa and Nacystelyn on transportability and viscoelasticity of cystic fibrosis sputum. Can. Respir. J..

[238] Addante A., Raymond W., Gitlin I., Charbit A., Orain X., Scheffler A.W., Kuppe A., Duerr J., Daniltchenko M., Drescher M. (2023). A novel thiol-saccharide mucolytic for the treatment of muco-obstructive lung diseases. Eur. Respir. J..

[239] Du Y., Chen Y., Li F., Mao Z., Ding Y., Wang W. (2023). Genetically Engineered Cellular Nanovesicle as Targeted DNase I Delivery System for the Clearance of Neutrophil Extracellular Traps in Acute Lung Injury. Adv. Sci..

[240] Dich H., Abou Rjeily R., Rath G., Berthet M., Dayde-Cazals B., Berret J.F., Angles-Cano E. (2025). Dual-functional cerium oxide nanoparticles with antioxidant and DNase I activities to prevent and degrade neutrophil extracellular traps. Front. Immunol..

[241] Tu Z., Liu M., Xu C., Wei Y., Lu T., Xiao Y., Li H., Zhang S., Xie X., Li J. (2024). Functional 2D Nanoplatforms Alleviate Eosinophilic Chronic Rhinosinusitis by Modulating Eosinophil Extracellular Trap Formation. Adv. Sci..

[242] Persson E.K., Verstraete K., Heyndrickx I., Gevaert E., Aegerter H., Percier J.M., Deswarte K., Verschueren K.H.G., Dansercoer A., Gras D. (2019). Protein crystallization promotes type 2 immunity and is reversible by antibody treatment. Science.

